# Isolation of arylhalodiphosphenes: periodic trends in R–P=P–X bonding (X = Cl, Br, I)

**DOI:** 10.1039/d6sc00723f

**Published:** 2026-02-25

**Authors:** John S. Wenger, Nina Gaschik, William J. Rowe, Agamemnon E. Crumpton, Bono van IJzendoorn, Meera Mehta

**Affiliations:** a Department of Chemistry, University of Oxford 12 Mansfield Road Oxford OX1 3QR UK john.wenger@chem.ox.ac.uk meera.mehta@chem.ox.ac.uk; b Department of Chemistry, Ludwig-Maximilians-Universität München Butenandtstrasse 5-13 81377 München Germany

## Abstract

For over a century, aryldiazonium halides have served as widely used building blocks within synthetic chemistry. They are vital intermediates in converting simple anilines to high-value products, including those needed to prepare pharmaceuticals, dyes, and functional materials. Despite the prevalence of these nitrogen-based organic salts in laboratories, structurally related phosphorus-based salts remain scarce. Herein, we report the isolation and structural characterization of a monomeric arylchlorodiphosphene, (M^s^FluInd*)P=PCl·(Et_2_O)_2_ (where M^s^FluInd* is a sterically demanding hydrindacene substituent), for the first time. The structure and reactivity of (M^s^FluInd*)P=PCl were explored to compare the novel arylhalodiphosphene with compositionally related aryldiazonium chlorides, [RNN][Cl], and chloroiminophosphanes, RN=PCl. The P–P bond of (M^s^FluInd*)P=PCl was cleaved *via* protonolysis to afford the parent phosphine, (M^s^FluInd*)PH_2_. Halogen-exchange reactions between (M^s^FluInd*)P=PCl and TMSX (TMS = trimethylsilyl, X = Br, I) afforded the related monomeric arylhalodiphosphenes, (M^s^FluInd*)P=PX (X = Br, I). Finally, the coordination complex, [(M^s^FluInd*)P=PCl·Ag][CF_3_SO_3_], was isolated by treatment of (M^s^FluInd*)P=PCl with AgCF_3_SO_3_. Periodic trends in the structure and bonding of (M^s^FluInd*)P=PX (X = Cl, Br, I) were investigated with spectroscopic, crystallographic, and computational methods. These studies confirm that the {PPX} moeity consists of a formal P–P double bond, and polar covalent P–X (X = Cl, Br, I) single bonds. (M^s^FluInd*)P=PX (X = Cl, Br, I) represent the first fully characterized, crystalline arylhalodiphosphenes and serve to advance the state of low-coordinate phosphorus chemistry.

## Abstract 2

Aryldiazonium halides have served as important synthetic intermediates for over a century. Herein, we report the synthesis of compositionally related monomeric arylhalodiphosphenes.

## Graphical abstract

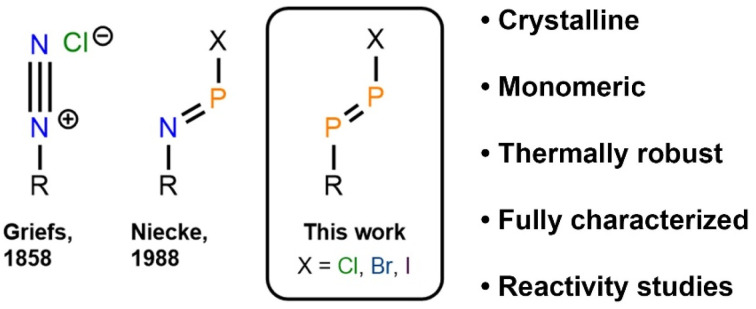

## Introduction

The isolation of heavy element analogues of common organic functional groups remains central to advancing our understanding of periodic trends and developing new precursors.^1,2^ The inherent challenges in stabilizing molecular species bearing multiple bonds between main-group elements heavier than those of the second period of the periodic table is encapsulated by the so-called “double bond rule”.^3–5^ Such molecules often form self-associated oligomers rather than retaining the heavy element–element multiple bond.^6^ Chemists may overcome this challenge by invoking thermodynamic stabilization whereby a Lewis acid and/or base is used to perturb the frontier molecular orbitals of the reactive unsaturated fragment,^7^ and/or *via* kinetic stabilization where the reactive fragment is sterically protected.^8,9^

**Fig. 1:**
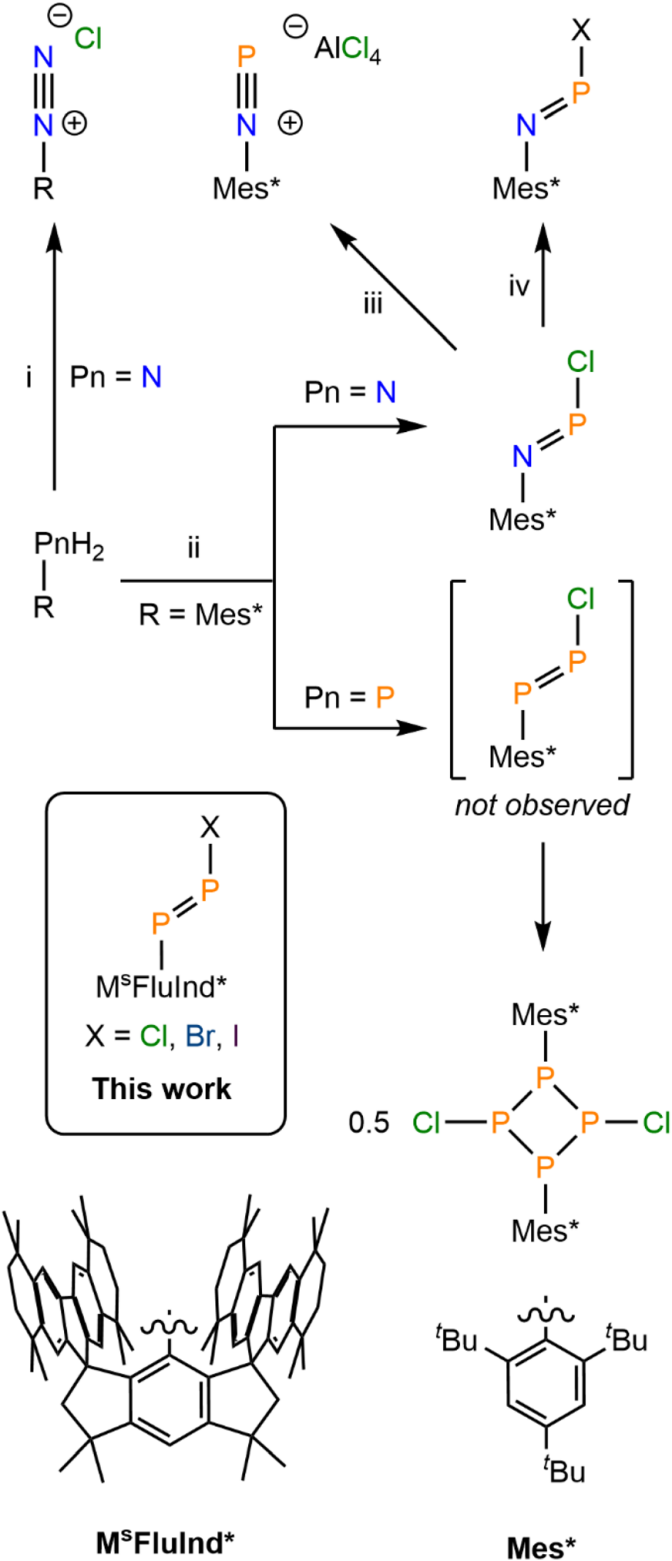
General synthesis of [RNN][Cl] (i = excess HCl, excess NaNO_2_). Synthesis of (Mes*)NPCl and [ClP(µ-PMes*)]_2_ (ii = excess PCl_3_, excess Et_3_N). Synthesis of [(Mes*)NP][AlCl_4_] (iii = AlCl_3_). Synthesis of (Mes*)NPX (iv = TMSX; X = Br, I). Depiction of (M^s^FluInd*)PPX (X = Cl, Br, I) reported herein. Structural diagrams of ligands M^s^FluInd* and Mes*.

Initially reported in 1858, aryldiazonium chlorides are conveniently prepared by treatment of anilines with HCl and NaNO_2_ (Fig. 1).^10^ Aryldiazonium salts have served as important reagents and intermediates in numerous key named reactions, including the Sandmeyer reaction,^11^ Pschorr reaction,^12^ Gomberg–Bachmann reaction,^13^ Balz–Schiemann reaction,^14^ and Meerwein arylations.^15^ These reagents continue to be under intense investigation for their powerful utility in converting simple anilines to value-added products *via* diverse synthetic pathways.^16^ Despite the prevalence of diazonium salts across synthetic chemistry, analogous species in which one or both of the diazonium N atoms are replaced by a heavier pnictogen remains rare. In an early report, (Mes*)N=PCl (Mes* = 2,4,6-tri-*tert*-butylphenyl) was isolated by treating the corresponding aniline with an excess of PCl_3_ and Et_3_N (Fig. 1).^17^ (Mes*)N=PCl may be viewed as a “monophosphadiazonium chloride” in which the Cl atom directly binds the terminal P atom, in contrast to diazonium chlorides which do not feature a similar N–Cl bond and instead exist as separated ion pairs even in the solid-state.^18^ Coordination of the Cl anion to the terminal P-atom disrupts multiple-bonding between the N and P atoms, and (Mes*)N=PCl features an N–P double bond. (Mes*)N=PCl may undergo halogen-exchange reactions to form (Mes*)N=PX (X = Br, I) by treating (Mes*)N=PCl with the corresponding trimethylsilyl (TMS) halide (Fig. 1).

(Mes*)N=PCl has served as a seminal precursor to phosphadiazonium compounds. For example, treatment of (Mes*)N=PCl with AlCl_3_ resulted in chloride abstraction to form the iminophosphenium tetrachloroaluminate, [(Mes*)NP][AlCl_4_] (Fig. 1). The same group later isolated a range of oxy-substituted iminophosphanes by treatment of (Mes*)N=PCl with silver or lithium salts.^19^ Further, iminophosphenium species may also be generated *in situ* by reacting with GaCl_3_.^20^ Recently, the chloroiminophosphane, (Ter*)N=PCl [Ter* = bis(*ortho-m*-hexaisopropylterphenyl)phenyl] was prepared from the bulky aniline (Ter*)NH_2_ and employed as a precursor in the isolation of a two-coordinate phosphindene oxide, (Ter*)NBnPO.^21^ Further, (M^s^FluInd*)N=PCl was recently prepared from (M^s^FluInd*)NH_2_ and served as a precursor for an aryliminophosphinyl radical, (M^s^FluInd*)NP.^22^

Numerous diphosphenes of the form RP=PR’ have been reported since the seminal discovery of the diaryldiphosphene, (Mes*)P=P(Mes*).^23^ The stability of diphosphenes is largely attributed to the presence of bulky substituents at each P atom that form a sterically protected cavity for the P–P double-bonded core to reside.^24^ Compounds featuring {P_2_} fragments stabilized by either carbene ligands or transition metals have also been isolated.^25–30^

Asymmetric diphosphenes that feature an alkyl, alkoxy, or amino group in addition to an aryl substituent have also been reported and, in some cases, have served as precursors to donor-stabilized diphosphadiazonium species.^31–34^ Treatment of the aminoaryldiphosphenes, (Mes*)P=PNR_2_ (NR_2_ = N(^*i*^Pr)_2_, N(cyclohexyl)_2_, or 2,2,6,6-tetramethylpiperidine) with one equivalent of HCF_3_SO_3_ affords ammonium salts, [(Mes*)P=PNHR_2_][CF_3_SO_3_], which are stable in solution at −50 °C.^35^ Remarkably, treatment of a 1 : 1 mixture of (Mes*)P=PNR_2_ and Ph_3_P with two equivalents of HCF_3_SO_3_ affords [(Mes*)P=P(PPh_3_)][CF_3_SO_3_], which was initially reported as a donor-stabilized diphosphadiazonium cation. However, crystallographic and computational data indicate that [(Mes*)P=P(PPh_3_)][CF_3_SO_3_] is best described as a diphosphene with an adjacent triphenylphosphonium center.^36^

Treatment of (Mes*)P=PN^*i*^Pr_2_ with HCl was reported to form monomeric (Mes*)P=PCl *via* the loss of HN^*i*^Pr_2_.^37^ However, (Mes*)P=PCl was reported to exist only transiently at −50 °C in solution, and its structure has only been inferred by ^31^P NMR analysis and follow-on reactivity studies with organolithium reagents to form asymmetric diphosphenes.^38^ Monomeric (Mes*)P=PX (X = Br, I) were reported to be prepared by treatment of (Mes*)P=PN^*i*^Pr_2_ with HX (X = Br, I), or by treatment of [(Mes*)P=P(PPh_3_)][CF_3_SO_3_] with [Et_3_NH][X] (X = Br, I) at −78 °C, but again these compounds were only characterized by ^31^P NMR spectroscopy and it is unclear at which temperature these spectra were collected.^35,39^ The existence of monomeric (Mes*)P=PX (X = Cl, Br, I) as isolable reagents has recently been called into question in the absence of conclusive analytical evidence and crystallographic characterization; treatment of the primary phosphine, (Mes*)PH_2_ with an excess of PCl_3_ and NEt_3_, does not afford monomeric (Mes*)P=PCl, but rather affords the dimeric form [ClP(µ-PMes*)]_2_, which was characterized in both solution and solid-state (Fig. 1).^36,40,41^

Herein, we report the isolation of a crystalline, monomeric arylchlorodiphosphene, (M^s^FluInd*)P=PCl (8) (Fig. 1). Spectroscopic, crystallographic, and computational characterization of compound 8 confirms the presence of a formal P–P double bond and a polar, covalent P–Cl single bond, in contrast to compositionally analogous aryldiazonium chlorides of the form [RNN][Cl]. The terminal {PPCl} unit in 8 is kinetically stabilized by the sterically demanding hydrindacene substituent, M^s^FluInd*.^42^ The unsaturated P–P bond in 8 may be cleaved *via* protonolysis to form the primary phosphine, (M^s^FluInd*)PH_2_ (4). Treatment of compound 8 with TMSBr or TMSI affords (M^s^FluInd*)PPBr (9), and (M^s^FluInd*)PPI (10), respectively, highlighting the synthetic utility of the terminal P–Cl bond in 8. Attempts to abstract the Cl atom from 8 with AlCl_3_ or GaCl_3_ afforded complex reaction mixtures, while treatment of 8 with AgCF_3_SO_3_ afforded the coordination complex, [(M^s^FluInd*)PPCl·Ag][CF_3_SO_3_] (11). Periodic trends in structure and bonding between the novel catalogue of isolable arylhalodiphosphenes 8, 9, and 10 were explored with spectroscopic, crystallographic, and computational methods.

## Results and discussion

### Synthesis of novel phosphine precursors

**Scheme 1:**
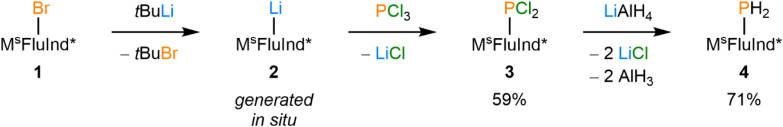
Synthesis of compounds 2, 3, and 4.

**Fig. 2:**
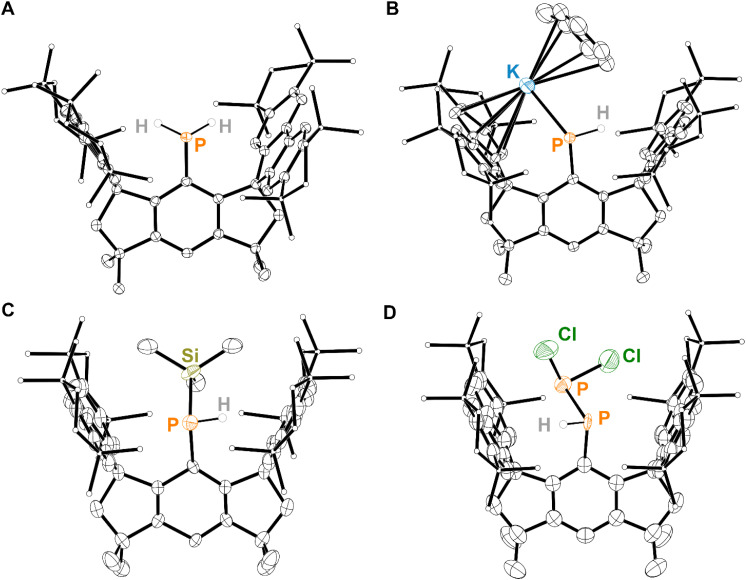
Thermal ellipsoid plot (50% probability) of (A) 4·(hexane), (B) 5·(toluene)_2.5_, (C) 6·(Et_2_O)_2_, and (D) 7·(Et_2_O)_2_. Solvent molecules, C-bound H atoms, and disordered components are omitted for clarity. Only the major component of disorder is displayed in all cases. Select C atoms and H atoms are shown as spheres of arbitrary radius for clarity. Color code: P orange, Cl dark green, Si dark yellow, K sky blue, C black, H grey.

Inspired by the success of bulky hydrindacene-based ligands in stabilizing reactive molecular fragments,^22,42–59^ literature known (M^s^FluInd*)Br (1) was treated with an excess of *tert*-butyl lithium to form (M^s^FluInd*)Li (2) *in situ*,^42^ which was subsequently treated with PCl_3_ to afford (M^s^FluInd*)PCl_2_ (3), characterized as the hexane solvate (Scheme 1). Compound 3 was then reduced with an excess of LiAlH_4_ to afford (M^s^FluInd*)PH_2_ (4) (Scheme 1). However, we found it was most efficient to synthesize 4 directly from 1 without fully isolating 3. Like other primary phosphines bearing bulky aryl substituents, 4 is air-stable in both the solid-state and in solution.^60,61^ Compound 4 could be crystallized from a −30 °C solution of hexane to afford colorless blocks of 4·(hexane). Structural characterization of 4·(hexane) by single-crystal X-ray diffraction (SC-XRD) confirms the presence of a terminal {PH_2_} group within the sterically protected environment created by the flanking fluorenyl substituents of the M^s^FluInd* ligand (Fig. 2A).

**Scheme 2:**
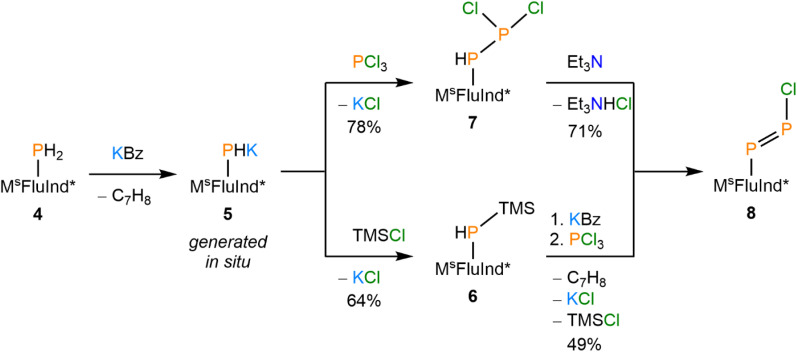
Synthesis of compounds 5, 6, 7, and 8.

Compound 4·(hexane) was reacted with potassium benzylate (KBz) in benzene to form intensely red solutions of (M^s^FluInd*)PHK (5) *in situ* (Scheme 2), which was structurally characterized as a toluene solvate (Fig. 2B). In the solid-state, 5·(toluene)_2.5_ exists as a centrosymmetric dimer in which a disordered toluene molecule resides on the crystallographic inversion center and coordinates the potassium ions. Each potassium ion is further coordinated by a 6-membered ring within the fluorenyl groups and by the anionic P-donor.

We hypothesized that a silylated phosphine substituted with the sterically demanding M^s^FluInd* substituent could serve as an effective precursor for the synthesis of unsaturated main-group species. (M^s^FluInd*)PTMSH (6) was isolated *via* sequential treatment of 4·(hexane) with KBz followed by TMSCl (Scheme 2). Colorless blocks of analytically pure 6·(Et_2_O)_2_ were isolated by crystallization from Et_2_O solutions at −30 °C. 6·(Et_2_O)_2_ crystallizes in the *P*4̄2_1_*m* space-group on a special position distinguished by a two-fold rotation axis and two mirror planes, such that 0.25 of the M^s^FluInd* ligand resides in the asymmetric unit and the central {PTMSH} unit is disordered about these symmetry elements (Fig. 2C).

Sequential treatment of 4·(hexane) with KBz followed by PCl_3_ successfully afforded (M^s^FluInd*)PHPCl_2_ (7) which was obtained as the Et_2_O disolvate from solutions of 7 in Et_2_O at −30 °C (Scheme 2). The ^31^P{^1^H} nuclear magnetic resonance (NMR) spectrum of 7·(Et_2_O)_2_ exhibits a prominent pair of doublets at −41 ppm and 209 ppm with a ^1^*J*_PP_ value of 247 Hz, consistent with the presence of a P–P single bond in 7.^62^ Additionally, the ^31^P and ^1^H NMR spectra confirm a P-bound proton in 7 with ^1^*J*_PH_ = 219 Hz and ^2^*J*_PH_ = 14.5 Hz. Crystals of 7·(Et_2_O)_2_ are crystallographically isomorphic with 6·(Et_2_O)_2_ and feature a terminal {PHPCl_2_} motif disordered about multiple positions (Fig. 2D). Unfortunately, we were unable to isolate 7·(Et_2_O)_2_ as an analytically pure material; we attribute our inability to purify 7 to the high crystallinity and similar solubility of M^s^FluInd*-containing impurities, a common challenge associated with the use of such sterically demanding substituents.^21,42,63^

### Synthesis and reactivity of a monomeric arylchlorodiphosphene

**Fig. 3:**
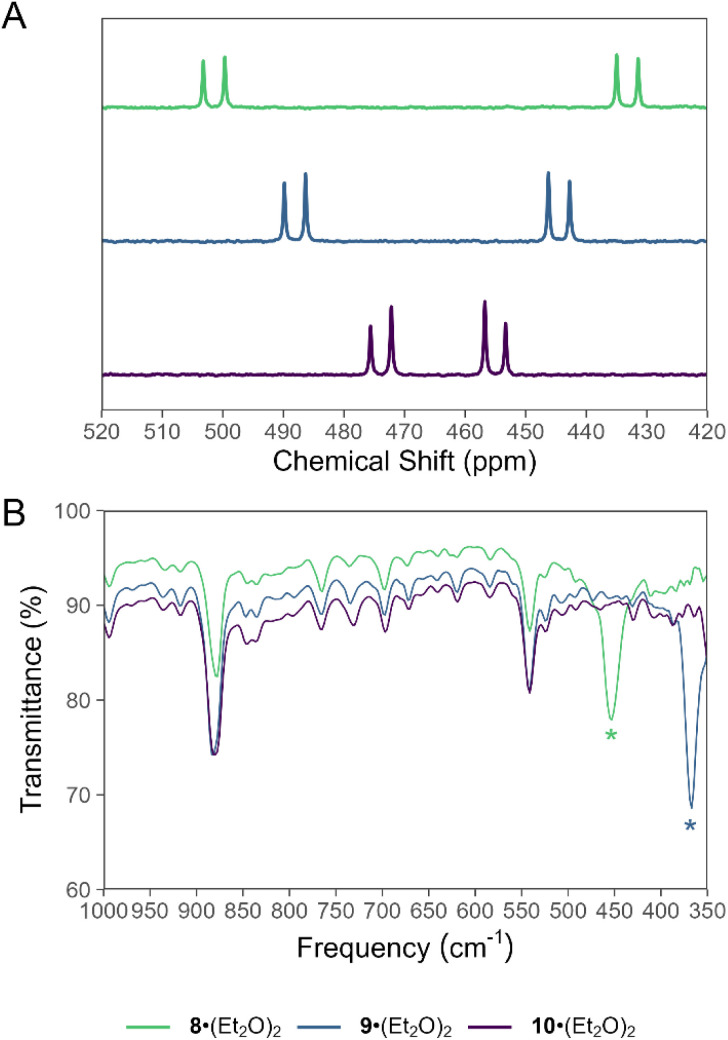
Stacked (A) ^31^P NMR spectra and (B) IR spectra of 8·(Et_2_O)_2_, 9·(Et_2_O)_2_, and 10·(Et_2_O)_2_. Signals in the IR spectrum assigned to a P–X bond stretching mode are denoted with an asterisk.

Treatment of a hexane solution of 7·(Et_2_O)_2_ with triethylamine resulted in the formation of a yellow suspension (Scheme 2). Removal of the solid by-product [Et_3_NH][Cl] and volatiles, followed by recrystallization of the residue from Et_2_O resulted in the isolation of yellow crystals. Analysis of the crystalline product by ^31^P NMR spectroscopy revealed two new doublets in the spectrum, which do not exhibit any ^1^H coupling (Fig. 3A). The ^31^P NMR resonances of the product are shifted strongly downfield with respect to the precursor at 433 ppm and 502 ppm and exhibit a larger ^1^*J*_PP_ coupling constant of 574 Hz, consistent with an asymmetric diphosphene species with a P–P double bond.^31,36,64–66^ Furthermore, the infrared (IR) spectrum of the product features a strong band assigned to the P–Cl stretch that appears at a lower wavenumber (*ν*_P–Cl_ = 451 cm^−1^) relative to that of 7·(Et_2_O)_2_ (*ν*_P–Cl_ = 461 cm^−1^) (Fig. 3B, SI Fig. S34).

**Fig. 4:**
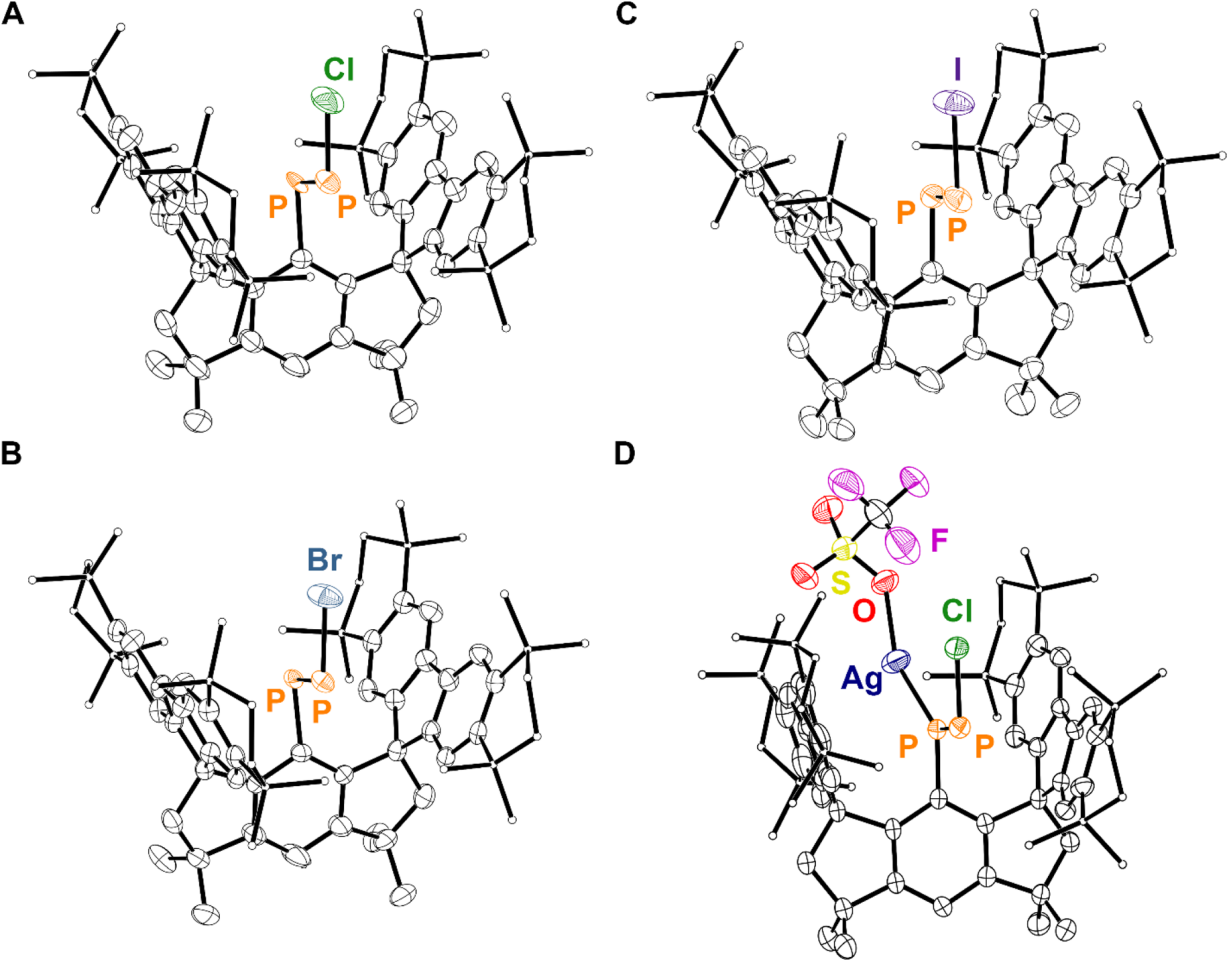
Thermal ellipsoid plots (50% probability) depicting the major *E*-isomer for (A) 8·(Et_2_O)_2_, (B) 9·(Et_2_O)_2_, (C) 10·(Et_2_O)_2_, and (D) 11. The refined *E* : *Z* occupancy ratios in our models are 85 : 15, 78 : 22, 60 : 40, and 67 : 33 for 8·(Et_2_O)_2_, 9·(Et_2_O)_2_, 10·(Et_2_O)_2_, and 11, respectively. Only the major component of disorder for the *E*-isomer of 8·(Et_2_O)_2_ and 9·(Et_2_O)_2_ are depicted. Solvent molecules, H atoms, and disordered components are omitted for clarity. Select C atoms are shown as spheres of arbitrary radius for clarity. Color code: P orange, Cl dark green, Br blue, I purple, Ag navy, F pink, S yellow, O red, C black.

Analysis of the yellow crystals by SC-XRD reveals the sample to be crystallographically isomorphic with those of 6·(Et_2_O)_2_ and 7·(Et_2_O)_2_; the product also crystallizes in the *P*4̄2_1_*m* space group with nearly identical unit cell parameters. However, solution of the solid-state structure confirms a distinct Fourier difference map within the cavity created by the M^s^FluInd* ligand. Indeed, the crystallographic data are fit excellently by 8·(Et_2_O)_2_ (*R*_1_ = 4.62%). Our model features disorder between a major *E*-isomer with an occupancy of 85% (Fig. 4A) and a minor *Z*-isomer with an occupancy of 15% with respect to the asymmetric diphosphene unit. The major *E*-isomer is further disordered about two positions, and the entire {PPCl} motif is disordered about a special position. We note that the connectivity of 8·(Et_2_O)_2_ is unambiguous, but meaningful discussion of structural parameters is precluded by this disorder. Remarkably, compound 8 may also be synthesized by treatment of 6·(Et_2_O)_2_ with KBz followed by PCl_3_*via* the formal elimination of KCl and TMSCl (Scheme 2).

For most monomeric diphosphenes, the synthesis of *Z*-isomers from *E*-isomers requires photolytic conditions and low temperatures, and warming solutions of the resulting *Z*-isomer to room-temperature results in the formation of the more stable *E*-isomer.^31,67,68^ The observed isomerism in the solid-state structure may be the result of a photoisomerization reaction during the diffraction experiment.^69^ The apparent isomerism identified in our diffraction study prompted us to perform variable-temperature NMR (VT NMR) studies. The ^31^P NMR resonances for 8 do indeed broaden at low temperature; however, it is unclear if there is dynamic exchange between the *E*- and *Z*-isomers in solution. Notably, the sterically encumbered arylhydrazinodiphosphene, RP=PMes* (R = (Me_3_Si)_2_N(SiMe_3_)N) can be isolated as either the *E* or *Z* isomer, and isomerizes at room temperature in solution to an equilibrium *E* : *Z* ratio of 11 : 6.^70^

**Scheme 3:**
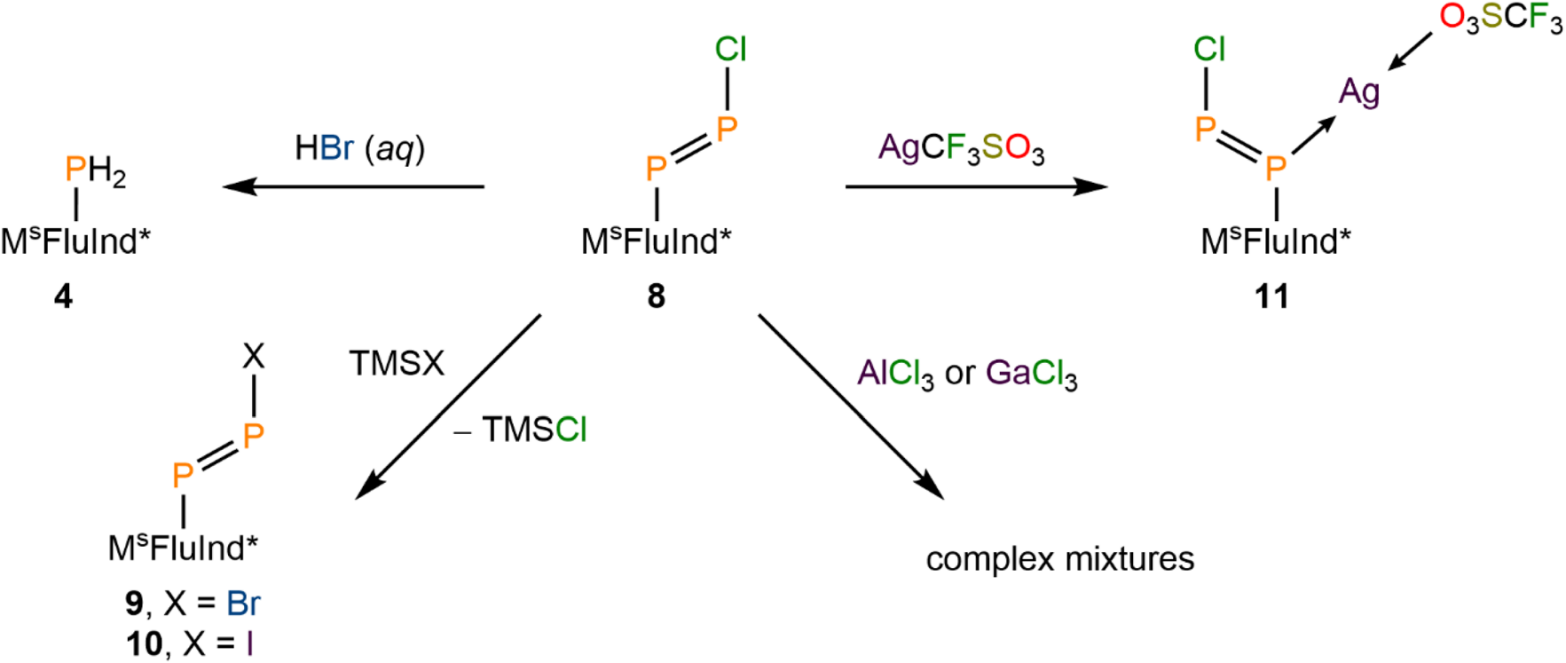
Reactivity of 8: Protonolysis of 8 to form 4. Synthesis of 9 and 10. Treatment of 8 with AlCl_3_ or GaCl_3_. Synthesis of 11.

Aryldiazonium halides are well known to form either phenols or aryl bromides in the presence of aqueous hydrobromic acid, *via* loss of the terminal {N_2_} unit.^16^ In contrast, treatment of a solution of 8·(Et_2_O)_2_ in C_6_D_6_ with an excess of concentrated hydrobromic acid in water results in the formation of compound 4*via* protonolysis of the P–P bond (Scheme 3). Unfortunately, we were unable to characterize the by-product from this protonolysis.

(Mes*)N=PCl was reported to engage in rapid halogen-exchange reactions in the presence of TMSBr or TMSI at 0 °C.^17^ Whilst compound 8 does not undergo similar reactions at 0 °C or room-temperature, heating toluene solutions of 8 in the presence of excess TMSBr or TMSI for 16 h at 100 °C results in the formation of 9 and 10, respectively (Scheme 3). The ^31^P NMR spectrum of 9 exhibits a characteristic pair of doublets at 490 and 445 ppm with a ^1^*J*_PP_ value of 567 Hz (Fig. 3A). Similarly, ^31^P NMR analysis of 10 reveals a pair of doublets at 474 and 457 ppm with a ^1^*J*_PP_ value of 554 Hz (Fig. 3A). With these values in hand, we note that the ^1^*J*_PP_ coupling constant decreases from 8 > 9 > 10, consistent with a systematic weakening of the P–P bond as the terminal halide increases in size. Further, the pair of doublets appears with a lower difference in chemical shift to each other from 8 > 9 > 10. The lower difference in chemical shift is consistent with a reduced polarity of the P–X bond (X = Cl, Br, I) as the X substituent becomes less electronegative from 8 > 9 > 10. Combustion analyses of compounds 8·(Et_2_O)_2_, 9·(Et_2_O)_2_, and 10·(Et_2_O)_2_ are consistent with expected elemental compositions; however, ^31^P{^1^H} NMR data for each of these species reveal the presence of trace impurities which could not be removed by recrystallization.

Comparison of the IR spectra of 8, 9, and 10 finds the expected decrease in wavenumber for the P–X stretch as the X atom becomes heavier (Fig. 3B). The P–Br stretch appears at 367 cm^−1^, while the P–I stretch is not observed in the spectral window. The fingerprint regions of 8, 9, and 10 are, essentially, indistinguishable.

Compounds 9·(Et_2_O)_2_ and 10·(Et_2_O)_2_ were each crystallized from Et_2_O at −30 °C and exhibit similar crystallographic isomorphism to 6·(Et_2_O)_2_, 7·(Et_2_O)_2_, and 8·(Et_2_O)_2_. The solid-state structures determined by SC-XRD for 9 and 10 each feature disorder between a major *E*-isomer and a minor *Z*-isomer (Fig. 4B and C).

After demonstrating the halogen-exchange reactivity of 8·(Et_2_O)_2_, we explored halogen-abstraction reagents in an effort to replace the Cl substituent with a weakly coordinating anion (Scheme 3). Heating solutions of 8·(Et_2_O)_2_ in toluene to 100 °C for 16 h in the presence of either TMS(CF_3_SO_3_), AlCl_3_, or GaCl_3_ resulted in incomplete conversion of 8 to a complex mixture of products (SI Fig. S71). However, if compound 8 is isolated in the absence of Et_2_O then reaction with AlCl_3_ or GaCl_3_ proceeds at room temperature, again to form a complex mixture of products (SI Fig. S72). We hypothesize that Cl-abstraction occurs in these reactions, followed by rapid decomposition of a transient diphosphadiazonium cation. Efforts to capture or detect this transient diphosphadiazonium cation are currently ongoing.

The iminophosphenium triflate, [(Mes*)NP][CF_3_SO_3_], may be prepared *via* treatment of (Mes*)N=PCl with AgCF_3_SO_3_, through the elimination of insoluble AgCl.^19^ In contrast, treatment of 8·(Et_2_O)_2_ with AgCF_3_SO_3_ under similar conditions affords the coordination complex 11, confirmed by SC-XRD studies (Scheme 3). The ^31^P{^1^H} NMR spectrum of 11 features a pair of broadened resonances at 463 and 351 ppm, consistent with interaction of the Ag ion with the diphosphene motif.^71,72^ Compound 8 is found to coordinate Ag^+^ in an η^1^-κ(P) mode that is commonly observed in coordination complexes involving diphosphenes, with the internal C-bound P atom coordinating to Ag^+^ (Fig. 4D).^31^ Remarkably, the solid-state structure of 11 features a two-component disorder arising from the presence of the *E* and the *Z* isomer of the coordinated diphosphene, 8, in a 68 : 32 occupancy ratio, respectively. These results ultimately highlight the divergent reactivity between the chloroiminophosphane, (Mes*)N=PCl, and compound 8.

### Theoretical analysis of monomeric arylhalodiphosphenes

Curious to investigate periodic trends in structure and bonding amongst this newly discovered class of monomeric arylhalodiphosphenes, the theoretical molecules *E*-8*, *E*-9*, *E*-10*, *Z*-8*, *Z*-9*, and *Z*-10* (pictured in the SI Fig. S73–S75) were optimized at the PBE0-D3/def2-TZVPP level of theory. Selected bond metrics are provided in SI Tables S5 and S6.

Frequency calculations predict the enthalpy of formation of *E*-8* to be 2.96 kcal mol^−1^ more favorable than that of *Z*-8*. Similarly, *E*-9* and *E*-10* are predicted to be more stable than *Z*-9* and *Z*-10* by 3.48 kcal mol^−1^ and 3.83 kcal mol^−1^, respectively. Calculated gas-phase ^31^P NMR spectroscopic data (PBE0-D4/pcsseg-2//PBE0-D3/def2-TZVPP) found the ^1^*J*_PP_ coupling constant for *E*-8* to be more consistent with our experimental value than that calculated for *Z*-8* (SI Table S21). Subsequent discussions are limited to the more stable theoretical *E*-isomers. The P–X bond stretching frequencies for *E*-8*, *E*-9*, and *E*-10* were calculated to be 481, 392, and 357 cm^−1^. The associated P–X stretching force constants are 2.10, 1.73, and 1.45 mdyne/Å, respectively, showing the weakening of the P–X bond from *E*-8* > *E*-9* > *E*-10*. The P–P bond stretching frequencies for *E*-8*, *E*-9*, and *E*-10* are 656, 653, and 649 cm^−1^ and predicted to have negligible intensity.

**Fig. 5:**
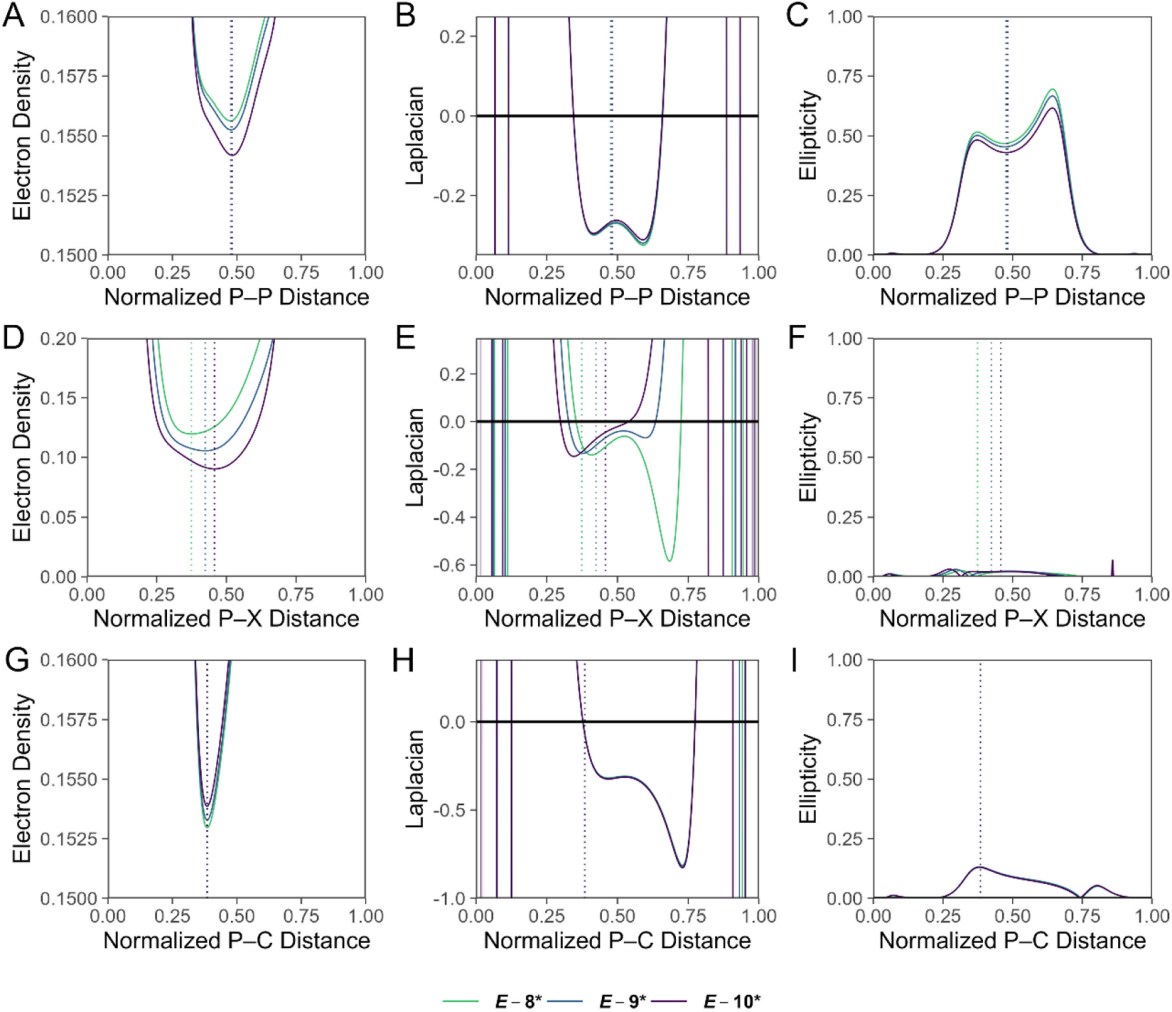
Values of (A) *ρ* (e^−^ Bohr^−3^), (B) ∇^2^*ρ* (e^−^ Bohr^−5^), and (C) *ε* for *E*-8*, *E*-9*, and *E*-10* along the P–P interatomic vector, with the C-bound P atom at 0.00 and the X-bound P atom (X = Cl, Br, I) at 1.00 along the horizontal axis. Values of (D) *ρ* (e^−^ Bohr^−3^), (E) ∇^2^*ρ* (e^−^ Bohr^−5^), and (F) *ε* for *E*-8*, *E*-9*, and *E*-10* along the P–X (X = Cl, Br, I) interatomic vector. Values of (G) *ρ* (e^−^ Bohr^−3^), (H) ∇^2^*ρ* (e^−^ Bohr^−5^), and (I) *ε* for *E*-8*, *E*-9*, and *E*-10* along the P–C interatomic vector. The bond lengths are normalized to 1.00. The location of the (3, −1) critical point is shown with a dashed vertical line. Calculations were performed at the (DKH-PBE0/old-DKH-TZVPP//PBE0-D3/TZVPP) level of theory.

A single point energy calculation at the DKH-PBE0/old-DKH-TZVPP level of theory was performed on the optimized coordinates of *E*-8*, *E*-9*, and *E*-10* for a detailed computational analysis (SI Tables S10–S17), and the summarized results are discussed. Topological analysis of the electron density (*ρ*)^73^ of *E*-8*, *E*-9*, and *E*-10* along the P–P interatomic vector reveals bond critical points, at which *ρ* = 0.156, 0.155, and 0.154 e^−^ Bohr^−3^, respectively (Fig. 5A). The negative Laplacian of *ρ* (∇^2^*ρ*) in the P–P valence region signifies significant charge concentration and covalency of the dipnictene bond of *E*-8*, *E*-9*, and *E*-10* (Fig. 5B).^74^ We also calculate significant ellipticity of *ρ* (*ε*) in the P–P valence region, consistent with formal double bond character of the interaction (Fig. 5C). Along the P–X (X = Cl, Br, I) interatomic vector, *ρ* = 0.120, 0.106, and 0.090 e^−^ Bohr^−3^ at the bond critical point for *E*-8*, *E*-9*, and *E*-10*, respectively, reflecting the weakening of the P–X bond from *E*-8* > *E*-9* > *E*-10* (Fig. 5D). The *∇*^2^*ρ* function in the P–Cl bonding region in *E*-8* is highly asymmetric and signifies a polarization of charge towards the more electronegative Cl atom, reflecting the ionic character of the bond (Fig. 5E). For *E*-9* and *E*-10*, the ∇^2^*ρ* function in the P–X (Br, I) bonding region is dramatically less polarized (Fig. 5E). Topological analysis along the P–C bond path in *E*-8*, *E*-9*, and *E*-10* is reminiscent of that of the P–Cl bond in *E*-8*, but features higher values of *ρ* at the bond critical point, and values of the ∇^2^*ρ* function in the bonding region are less polarized and more negative, consistent with a stronger, more covalent bonding interaction (Fig. 5G and H). Values for *ε* along the P–X (X = Cl, Br, I) and P–C bond paths in *E*-8*, *E*-9*, and *E*-10* are low, but not negligible and likely signify π-type donor–acceptor interactions (Fig. 5F and I).

**Fig. 6:**
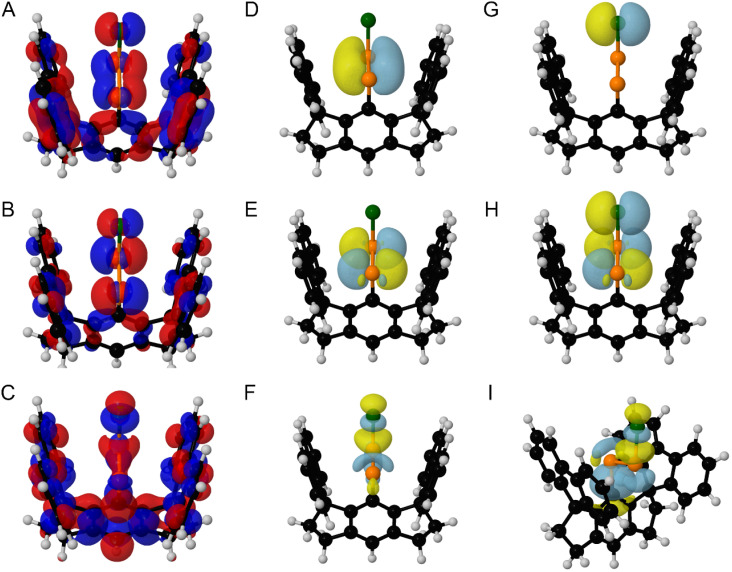
(A) HOMO, (B) LUMO, and (C) LUMO+2 of *E*-8* (isovalue = 0.015). Surface plots (isovalue = 0.06) for *E*-8*, depicting the (D) P–P π bonding NLMO, (E) P–P π* antibonding NLMO, (F) P–Cl σ* antibonding NLMO, (G) a Cl-centered lone pair, (H) overlap between a Cl-centered lone pair and the P–P π* antibonding NLMO, and (I) overlap between the P–C *σ* bonding NLMO and the P–Cl σ* antibonding NLMO. In Fig. 6A–H, the molecule is viewed down the plane defined by the {PPCl} unit. In Fig. 6I, the molecule is oriented differently for clarity of the displayed NLMOs. Color code: P orange, Cl dark green, C black, H grey. Displayed NLMOs are pre-orthogonalized. Calculations were performed at the (DKH-PBE0/old-DKH-TZVPP//PBE0-D3/TZVPP) level of theory.

The highest occupied molecular orbital (HOMO) of *E*-8* is highly delocalized and largely comprised of contribution by the P–P π bond and a Cl-centered lone pair (Fig. 6A). The lowest unoccupied molecular orbital (LUMO) of *E*-8* is largely defined by P–P π* contribution and the LUMO+2 of *E*-8* is largely defined by P–Cl σ* contribution (Fig. 6B and C). Similar results were obtained for *E*-9* and *E*-10*; however, the LUMO+1 is largely comprised of the P–X σ* (X = Br, I) contribution in these cases (SI Fig. S77–S82).

**Fig. 7:**
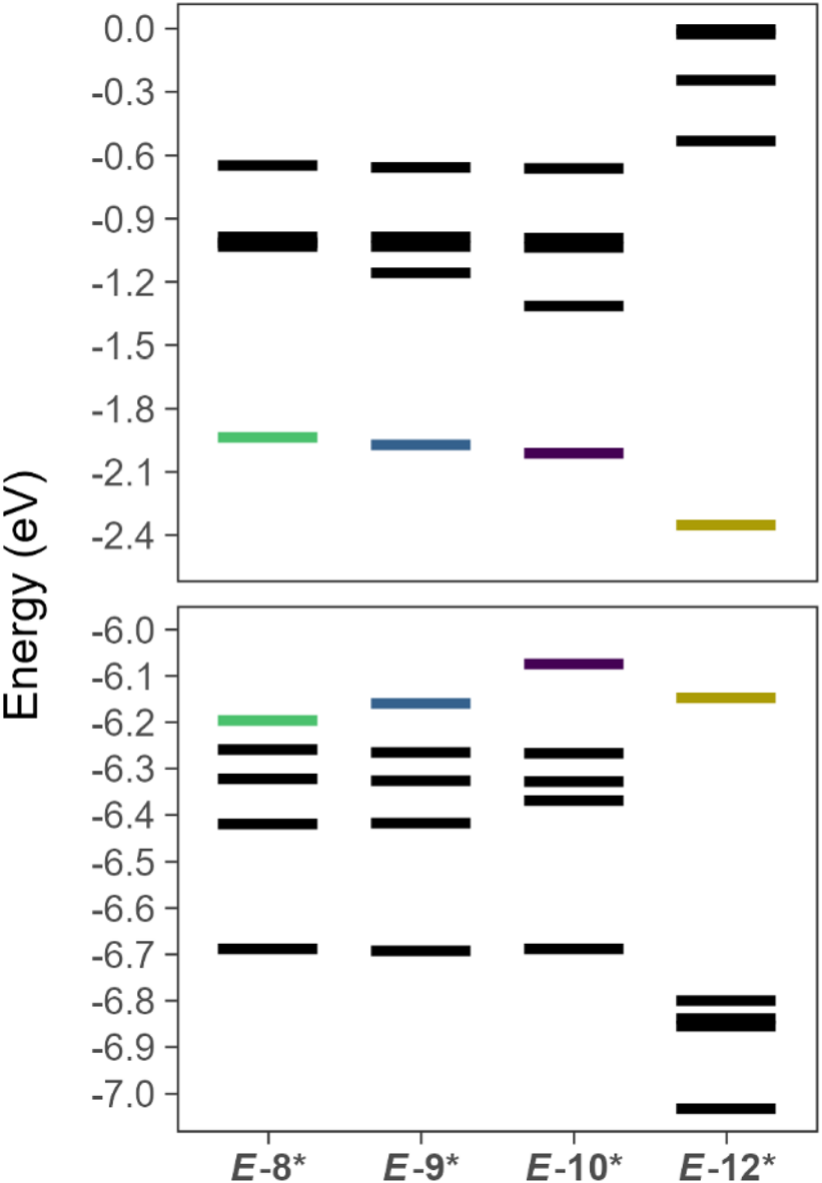
Calculated orbital energies (DKH-PBE0/old-DKH-TZVPP//PBE0/def2-TZVPP) for *E*-8*, *E*-9*, *E*-10*, and *E*-12* with frontier molecular orbitals shown in color. The upper panel contains the values for the LUMO, LUMO+1, LUMO+2, LUMO+3, and LUMO+4. The lower panel contains values for the HOMO, HOMO–1, HOMO–2, HOMO–3, and HOMO–4.

Energetic analysis of the canonical molecular orbitals (CMO) reveals the HOMO increases in energy from *E*-8* < *E*-9* < *E*-10* while the LUMO and LUMO+1 decrease in energy from *E*-8* > *E*-9* > *E*-10* (Fig. 7). These data are consistent with the weakening of the P–P and P–X (X = Cl, Br, I) bonds as the terminal halide becomes heavier. We performed a similar energetic analysis of the simple, theoretical diaryldiphosphene, *E*-MesP=PMes (*E*-12*), for comparison, and *E*-12* features a significantly lower LUMO than the theoretical arylhalodiphosphenes (Fig. 7).

Experimental ultraviolet-visible (UV-Vis) spectra of 8·(Et_2_O)_2_, 9·(Et_2_O)_2_, and 10·(Et_2_O)_2_ feature *λ*_max_ values of 349 nm, 355 nm, and 370 nm, respectively, reflecting the lowering of the LUMO from *E*-8* > *E*-9* > *E*-10*. Time-dependent density functional theory (TD-DFT) calculations suggest that the observed yellow color in these species is predicted to arise from electronic transitions between the HOMO–3/HOMO–2/HOMO–1/HOMO and the LUMO in each case (SI Tables S12–S14).

Natural Population Analysis (NPA) reveals a systematic decrease in negative charge on the halide from *E*-8* > *E*-9* > *E*-10* (SI Fig. S84). Similarly, a decrease in positive charge is calculated for the X-bound P atom from *E*-8* > *E*-9* > *E*-10*. These results are consistent with a less polarized P–X bond as the halide increases in size. The theoretical asymmetric diphosphenes, *E*-MesP=P(^*t*^Bu) (*E*-13*), *E*-MesP=P(SiMe_3_) (*E*-14*), *E*-MesP=P(OMe) (*E*-15*), *E*-MesP=P(NMe_2_) (*E*-16*), [*E*-MesP=P(NMe_3_)]^+^ (*E*-17*), and [*E*-MesP=P(PMe_3_)]^+^ (*E*-18*) were investigated at the same level of theory to compare polarization of the P–P bond in arylhalodiphosphenes with other asymmetric diphosphenes (pictured in the SI Fig. S83). NPA analysis suggests that among the arylhalodiphosphenes, the P–P bond becomes increasingly polarized from *E*-8* < *E*-9* < *E*-10*, as the positive charge on the X-bound (X = Cl, Br, I) P atom decreases. The alkyl-substituted diphosphene, *E*-13* features a relatively unpolarized P–P bond, similar to *E*-8* and *E*-9*. However, the heteroatom-substituted aryldiphosphenes feature relatively large polarization of the P–P bond, which is more akin to *E*-10*, with the exception of the ammonium cation, *E*-17*.

Natural Localized Molecular Orbital (NLMO) analysis of *E*-8* reveals the presence of a P–P π NLMO and a P–P π* NLMO, which closely resemble the nodal structure calculated for the HOMO and LUMO, respectively (Fig. 6D and E). The NLMO analysis further identified a P–Cl σ* NLMO which resembles the LUMO+2 and a filled Cl-centered 3p orbital, which appears prominently in both the HOMO and LUMO (Fig. 6F and G).

Intriguingly, second-order perturbation theory analysis of *E*-8*, *E*-9*, and *E*-10* reveals delocalization from an X-centered (X = Cl, Br, I) lone pair to the P–P π* orbital to afford an energy of stabilization of 9.39, 7.82, and 6.30 kcal mol^−1^, respectively (Fig. 6H). Furthermore, delocalization of electron density from the P–C *σ* orbital and the lone pair of the C-bound P atom to the terminal P–X σ* orbital (X = Cl, Br, I) afford a total energy of stabilization of 4.73, 4.92, and 4.53 kcal mol^−1^ for *E*-8*, *E*-9*, and *E*-10*, respectively (Fig. 6I). In order to more broadly assess the relative strengths of the non-covalent donor–acceptor interactions present in *E*-8*, *E*-9*, and *E*-10*, we performed deletion calculations, in which all non-covalent interactions from the halide to the {P_2_} unit and *vice versa* were deleted. The removal of these non-covalent interactions resulted in the destabilization of *E*-8*, *E*-9*, and *E*-10* by 30.73, 25.73, and 19.96 kcal mol^−1^, respectively. The results of these deletion calculations are in line with the general trend that non-covalent interactions between the halide and the {P_2_} unit become less efficient from *E*-8* > *E*-9* > *E*-10*.

Natural Resonance Theory (NRT) analysis identified leading resonance structures featuring a P–P double bond and a polar, covalent P–X (X = Cl, Br, I) single bond for the simple theoretical molecules *E*-MePPCl, *E*-MePPBr, and *E*-MePPI, respectively (SI Tables S18–S20). The NRT analysis is consistent with increasing ionicity of the P–X bond from *E*-MePPI < *E*-MePPBr < *E*-MePPCl.

## Conclusions

In conclusion, we report the isolation of 8·(Et_2_O)_2_, a thermally robust, monomeric arylhalodiphospene. Compound 8 features a terminal {PPCl} unit and can thus be regarded as a ‘masked’ aryldiphosphadiazonium chloride, marking a significant advancement in the context of decades of diphosphene chemistry. The P–P bond of 8 could be cleaved *via* protonolysis by aqueous hydrobromic acid, and the synthetic utility of the P–Cl bond in 8 was demonstrated in halogen-exchange reactions with TMSBr and TMSI to form the monomeric arylhalodiphosphenes, 9 and 10, respectively. Treatment of 8 with GaCl_3_ or AlCl_3_ resulted in rapid decomposition to form complex reaction mixtures. However, 8 participates in coordination chemistry that is typical of diphosphenes; treatment of 8 with AgCF_3_SO_3_ forms, 11. In 8, 9, 10, and 11, SC-XRD experiments clearly identified the presence of both the *E* and the *Z* isomer with respect to the diphosphene motif in solid state. Our theoretical investigation elucidated trends in bonding amongst the theoretical arylhalodiphosphenes *E*-8*, *E*-9*, and *E*-10*. Notably, the HOMO increases in energy, the LUMO and LUMO+1 decrease in energy, and the P–X (X = Cl, Br, I) bond weakens as the terminal halide increases in size. Further investigations into the reactivity of monomeric arylhalodiphosphenes are currently underway, as are efforts to isolate a genuine diphosphadiazonium salt.

## Author contributions

J. S. W.: conceptualization, data curation, funding acquisition, investigation (chemical synthesis, data acquisition, X-ray crystallography, DFT methods), methodology, visualization, writing – original draft, writing – review and editing. N. G.: investigation (chemical synthesis). W. J. R.: investigation (chemical synthesis). A. E. C.: investigation (X-ray crystallography). B. v. I.: investigation (acquisition of VT-NMR data). M. M.: conceptualization, funding acquisition, project administration, resources, supervision, writing – review and editing.

## Conflicts of interest

There are no conflicts to declare.

## Supplementary Material

SC-OLF-D6SC00723F-s001

SC-OLF-D6SC00723F-s002

SC-OLF-D6SC00723F-s003

## Data Availability

CCDC 2501235–2501241, 2512823, 2512824, and 2523752 contain the supplementary crystallographic data for this paper.^75*a*–*j*^ The data supporting this article have been included as part of the supplementary information (SI). Supplementary information is available. See DOI: https://doi.org/10.1039/d6sc00723f.
