# Supplementary material for: Isolation of arylhalodiphosphenes: periodic trends in R–P[double bond, length as m-dash]P–X bonding (X = Cl, Br, I)
Source: Chem Sci. 2026 Feb 25;17(15):7475–85. doi: 10.1039/d6sc00723f (PMC12934508; doi:10.1039/d6sc00723f)
Supplement: SC-017-D6SC00723F-s001 [file SC-017-D6SC00723F-s001.pdf]

Supplementary Information (SI) for Chemical Science.
This journal is © The Royal Society of Chemistry 2026

Supporting Information for

Isolation of Arylhalodiphosphenes:

Periodic trends in R–P═P–X bonding (X = Cl, Br, I)

John S. Wenger,*[a] *Nina Gaschik,*[b] *William J. Rowe,* [a] *Agamemnon E. Crumpton,* [a]

Bono van IJzendoorn,[a] *and Meera Mehta**[a]

[a] Department of Chemistry, University of Oxford, 12 Mansfield Road, Oxford, OX1 3QR, U.K. john.wenger@chem.ox.ac.uk, meera.mehta@chem.ox.ac.uk

[b] Department of Chemistry, Ludwig-Maximilians-Universität München, Butenandtstrasse 5-13, 81377 München, Germany

Contents

1. Experimental Methods 3

2. Synthesis and characterization of novel compounds. 10

2.1 Synthesis of ($M^sFluInd^*$)PCl_2•(hexane) (3•(hexane)). 10

2.2 Synthesis of ($M^sFluInd^*$)PH_2•(hexane) (4•(hexane)). 14

2.3 Synthesis of ($M^sFluInd^*$)PTMSH•$(Et_2O)_2$ (6•$(Et_2O)_2$) 21

2.4 Synthesis of ($M^sFluInd^*$)$PHPCl_2$•$(Et_2O)_2$ (7•$(Et_2O)_2$) 26

2.5 Synthesis of ($M^sFluInd^*$)PPCl•$(Et_2O)_2$ (8•$(Et_2O)_2$) 32

2.6 Synthesis of ($M^sFluInd^*$)PPBr•$(Et_2O)_2$ (9•$(Et_2O)_2$) 42

2.7 Synthesis of ($M^sFluInd^*$)PPI•$(Et_2O)_2$ (10•$(Et_2O)_2$) 48

2.8 Synthesis of [($M^sFluInd^*$)PPCl•Ag][CF_3SO_3]•(hexane) (11•(hexane)) 55

2.9 Crystal growth of ($M^sFluInd^*$)Li•(Et_2O)•$(toluene)_2$ (2•(Et_2O)•$(toluene)_2$). 64

2.10 Crystal growth ($M^sFluInd^*$)PHK•$(toluene)_{2.5}$ (5•$(toluene)_{2.5}$). 65

2.11 Protonolysis of 8•$(Et_2O)_2$. 67

2.12 Treatment of 8•$(Et_2O)_2$ with halogen-abstraction reagents 69

2.13 Treatment of 8 with halogen-abstraction reagents 70

3. Crystallographic Tables 71

4. Computational Data 76

5. References 112

1. Experimental Methods

General Methods. Dimethyl isophthalate, *tert*-butyl lithium (1.7 M in pentane), PCl_3, $AlCl_3$, BCl_3 (1M in hexane mixed isomers), $GaCl_3$, trimethylsilyl bromide (TMSBr), trimethylsilyl iodide (TMSI), trimethylsilyl trifluoromethanesulfonate ($TMS(CF_3SO_3)$), $AgCF_3SO_3$, $LiAlH_4$, Et_3N, and $Et_4NOH•(H_2O)_5$ were purchased from Sigma-Aldrich. Fluorene, sulfuric acid, hydrochloric acid (aq.), and methylmagnesium bromide (3 M in diethyl ether) were purchased from Thermo Fisher Scientific. 2,5-Dimethyl-2,5-hexanediol was purchased from Fluorochem. Trimethylsilyl chloride (TMSCl) was purchased from ChemCruz. N-Bromosuccinimide was purchased from Alfa Aesar. Reagents purchased from commercial vendors were used as received. ($M^sFluInd$)*Br was synthesized as previously reported;[1] however, we provide the overall synthetic route below with literature references for the synthesis of each precursor employed in this work. Potassium benzylate (KBz) was synthesized as previously reported.[2] All manipulations were performed under an inert atmosphere using standard Schlenk line, and glovebox (MBraun Unilab) techniques, except for the aqueous work-up described for the synthesis of **4**•(hexane) and the protonolysis reaction between **8** and aqueous hydrobromic acid. Glassware was flame dried prior to use. Glass filter papers were oven-dried prior to use. Solvents diethyl ether (Et_2O), benzene, toluene, hexane, dichloromethane (DCM), and acetonitrile were purified using an Innovative Technologies anhydrous engineering solvent purification system and degassed prior to being stored on 3 Å molecular sieves. C_6D_6 and C_7D_8 were degassed and stored on 3 Å molecular sieves. Et_3N was dried over CaH_2 for 24 h before being purified by distillation and stored under inert atmosphere.

NMR Spectroscopy. ^1H, $^{13}C\{^1H\}$, ^{31}P, $^{31}P\{^1H\}$, and $^{19}F\{^1H\}$ were recorded on a Bruker AVIII 400 (operating frequencies: 400.20 MHz, 100.64 MHz, 162.00 MHz, and 376.53 for ^1H, ^{13}C, ^{31}P, and ^{19}F respectively) or Bruker AVIII 500 (operating frequencies: 499.94 and 202.37 MHz for ^1H and ^{31}P respectively) spectrometer. ^1H and $^{13}C\{^1H\}$ NMR spectra were referenced internally to residual solvent signals ^1H δ = 7.16 ppm, $^{13}C\{^1H\}$ δ = 128.02 ppm for C_6D_6 or ^1H δ = 6.96 ppm for C_7D_8. ^{31}P and $^{31}P\{^1H\}$ spectra were referenced externally to H_3PO_4. $^{19}F\{^1H\}$ spectra were referenced externally to $CFCl_3$. Solution phase

NMR samples were prepared under an inert atmosphere in 5 mm J Young NMR tubes. NMR data were analyzed using MestReNova software.

X-ray Crystallography. X-ray diffraction data were collected on a dual wavelength Rigaku FR-X rotating anode diffractometer equipped with an AFC-11 4-circle kappa geometry goniometer, VariMAXTM microfocus optics, a Hypix-6000HE detector and an Oxford Cryosystems Cryostream 800 nitrogen flow gas system. Data were collected and reduced using Rigaku CrysAlisPro (version 43).[3] The structures were solved using SHELXT and refined using SHELXL within the suite of programs provided by Olex2,[4] following established strategies.[5-7] All non-H atoms were refined anisotropically. C-bound H atoms were placed at calculated positions and refined with a riding model and coupled isotropic displacement parameters (1.2 × Ueq for non-methyl C-H atoms and 1.5 × Ueq for methyl groups). In the case of **4**•(hexane) and **5**•(toluene)$_{2.5}$, P-bound H atoms were located in the Fourier difference map, refined with chemically reasonable distance restraints, and treated with coupled isotropic displacement parameters (1.2 × Ueq). In the case of **6**•$(Et_2O)_2$ and **7**•$(Et_2O)_2$, P-bound H atoms could not be reliably located in the Fourier difference map, presumably due to crystallographic disorder, and were placed at calculated positions, refined with chemically reasonable distance restraints, and treated with a coupled isotropic displacement parameters (1.2 × Ueq). Disordered components were modelled with similarity (SIMU), rigid bond (RIGU), and distance (SADI) restraints where appropriate. Non-default restraint values were employed when chemically reasonable and necessary for stable refinement. The terminal {PCl_2} group of **3**•(hexane) is disordered about two-positions. The terminal {PH_2} group of **4**•(hexane) is disordered about two-positions. The terminal {PH} group of **5**•(toluene)$_{2.5}$ is disordered about two-positions, and the K-bound toluene molecule resides on a crystallographic inversion center such that it is disordered about two symmetry-equivalent positions. Compound **7**•$(Et_2O)_2$ was refined as an inversion twin. The diffraction data for **8**•$(Et_2O)_2$, **9**•$(Et_2O)_2$, **10**•$(Et_2O)_2$, and **11** are fit excellently by models which consist of co-crystallized *E*/*Z* isomers, which appear as disordered components within the same crystal structure. The refined *E* : *Z* occupancy ratios in our models are 85 : 15, 78 : 22, 60 : 40, and 68 : 32 for **8**•$(Et_2O)_2$, **9**•$(Et_2O)_2$, **10**•$(Et_2O)_2$, and **11**, respectively. For **8**•$(Et_2O)_2$ and **9**•$(Et_2O)_2$, the terminal {PPX} (X = Cl or Br) unit was modelled in a three-part disorder (two

corresponding to the major *E*-isomer and one corresponding to the minor *Z*-isomer) with the free variables set to sum to 0.25, employing the SUMP command in SHELXL. For **10**•$(Et_2O)_2$, the terminal {PPI} unit was modelled as a two-part disorder which sum to 0.25, with one part corresponding to the *E* isomer and one art corresponding the *Z* isomer. As **8**, **9**, and **10** reside on special positions defined by two mirror planes and a two-fold rotation axis, the {PPX} (X = Cl, Br, I) unit is further disordered into four symmetry-equivalent parts and is modelled as a total 12-part disorder (for X = Cl and Br) or a total eight-part disorder (for X = I), with a total occupancy of one. For **6**•$(Et_2O)_2$, the terminal {PHTMS} group resides on a special position leading to an overall four-part disorder. For **7**•$(Et_2O)_2$, the terminal {$PHPCl_2$} unit was modelled as a two-part disorder that is further disordered about the special position on which it resides leading to an overall eight-part disorder. Furthermore, the solvated Et_2O molecules in **7**•$(Et_2O)_2$, **8**•$(Et_2O)_2$, **9**•$(Et_2O)_2$, and **10**•$(Et_2O)_2$ and methyl groups on the hydrindacene backbone of **8**•$(Et_2O)_2$, **9**•$(Et_2O)_2$, and **10**•$(Et_2O)_2$ are disordered in two parts about special positions. The severe disorder present in **6**•$(Et_2O)_2$, **7**•$(Et_2O)_2$, **8**•$(Et_2O)_2$, **9**•$(Et_2O)_2$, and **10**•$(Et_2O)_2$ preclude much meaningful discussion of bond metrics; however, the connectivity in our models is unambiguous and the data is fit excellently by our models. The crystal structure of **11** contained severely disordered solvent (likely one benzene molecule and half of a hexane molecule per asymmetric unit), which could not be reliably modelled; as such, **11** was refined with a solvent mask in *Olex2*. The solvent mask applied identified two voids per unit cell, each approximating a volume of 836.1 Å^3 and 150.3 electrons, consistent with the presence of highly disordered hydrocarbon solvent molecules in the crystal. The reported chemical formula and properties do not include the disordered solvent that was not modelled. **11** does not reside on a special position, and the terminal {PCl} group is disordered into two parts that sum to a total occupancy of one, which correspond to the co-crystallized *E* and *Z* isomer of Ag-bound **8** identified in solid state. Crystallographic data for **2**-**11** has been deposited *via* the joint CCDC/FIZ Karlsruhe deposition service under 2501235-2501241, 2512823, 2512824, and 2523752, respectively.

Powder X-ray Diffraction. Powder X-Ray diffraction was measured using a PANalytical Empyrean diffractometer operating in the Bragg-Brentano geometry with a Ge(111) monochromator to select only the CuK_alpha1 radiation with a PIXcel1D, low background

silicon strip direct detector. An X-offset of 2θ = 0.25° was applied to the diffractogram of **8**•$(Et_2O)_2$ to correct for instrumental error. Simulated powder diffractograms were calculated by Mercury (version 2024.2.0) from SC-XRD data.

Elemental analysis. Elemental analyses were performed by the analytical service of London Metropolitan University, where samples were weighed using a Mettler Toledo high precision scale and analyzed using a ThermoFlash 2000.

Mass spectrometry. Samples for mass spectrometry were prepared by diluting 100 μL of a 1 mg/mL stock solution of analyte in DCM with 900 μL of acetonitrile under inert atmosphere. The resulting mixture was filtered through glass filter paper before being injected into an electrospray ionization (ESI) equipped Waters RDa bench-top time of flight mass spectrometer provided by the mass spectrometry service of the University of Oxford. Mass spectra were simulated using the online Prot Pi Mass Spectrum Simulator.

Infrared spectroscopy. ATR-IR spectra were recorded on microcrystalline solids using a Bruker Alpha II under a dry N_2 atmosphere.

UV-Vis spectroscopy. Ultraviolet-visible (UV-Vis) electronic absorption spectra were recorded using a Mettler Toledo UV5Bio spectrophotometer. Samples were prepared under inert atmosphere and analyzed in 10 mm path length quartz J Young cuvettes.

Computational Methods. ORCA version 5.0.1 was used for all quantum chemistry calculations.[8] The experimental coordinates obtained for **8**•$(Et_2O)_2$ by SC-XRD were loaded into Mercury (version 2024.2.0) and edited by removing solvent molecules, alkyl groups, and disordered components, and normalizing H atoms, resulting in the truncated theoretical molecules, referred to as ***E*-8*** and ***Z*-8***. Initial coordinates for ***E*-MePPCl** were obtained similarly; all C and H atoms were removed except the P-bound C atom and three sp^3 H atoms were placed at calculated positions. A similar process was conducted to obtain initial coordinates for ***E*-12***, ***E*-13***, ***E*-14***, ***E*-15***, ***E*-16***, ***E*-17***, and ***E*-18*** from experimental coordinates of Mes*PPMes*.[9] Initial coordinates for ***E*-9***, ***Z*-9***, ***E*-MePPBr**, ***E*-MePPI** were obtained by replacing the Cl atom from the respective Cl analogue for a Br or I atom. The coordinates for ***E*-8***, ***E*-9***, ***E*-10***, ***E*-12***, ***E*-13***, ***E*-14***, ***E*-15***, ***E*-16***, ***E*-17***, ***E*-18***, ***Z*-8***, ***Z*-9***, ***Z*-10***, ***E*-MePPCl**, ***E*-MePPBr**, and ***E*-MePPI** were optimized

employing the BP86 pure functional, the def2-TZVP basis set, and Grimme's D3 dispersion correction, with the RI approximation and def2/J auxiliary basis set.[10, 11] The resulting optimized coordinates were then used as input for a second geometry optimization employing the PBE0 hybrid functional, def2-TZVPP basis set, and Grimme's D3 dispersion correction with the RIJCOSX approximation and def2/J auxiliary basis set.[12-16] A frequency calculation was performed on the resulting optimized coordinates (PBE0-D3/def2-TZVPP) at the same level of theory and no imaginary vibrational modes were calculated, suggesting the coordinates had optimized to a local minimum within acceptable convergence criteria, with the exception of ***E*-12***, ***E*-13***, ***E*-14***, ***E*-15***, ***E*-16***, ***E*-17***, and ***E*-18***, which exhibit imaginary vibrational modes arising from methyl rotations. A single point energy calculation was performed on the optimized coordinates using the PBE0 hybrid functional and old-DKH-TZVPP all-electron, relativistically contracted basis set using the Douglas-Kroll Hess formalism, the RIJCOSX approximation, and the SARC/J auxiliary basis set.[17-20] The wavefunctions obtained for ***E*-8***, ***E*-9***, and ***E*-10*** following this single point energy calculation (DKH-PBE0/old-DKH-TZVPP//PBE0-D3/def2-TZVPP) were then subject to topological and orbital-based analyses. A TD-DFT calculation was performed for ***E*-8***, ***E*-9***, and ***E*-10*** (DKH-PBE0/old-DKH-TZVPP//PBE0-D3/def2-TZVPP). We performed topological analyses[21] within MultiWFN (version 3.7). Real space functions for the electron density (ρ), Laplacian of the electron density ($\nabla^2\rho$), and ellipticity of the electron density (ε) along the P–P interatomic vector and in the plane defined by the P1, P2, and X3 (X = Cl, Br, I) atoms were visualized using R (version 4.0.2) through Rstudio (version 1.3.1073). We employed the following R packages for analysis and visualization: ggplot2, tidyverse, gridExtra, ggtext, scales, and grid. NBO analysis was performed with the NBO program (version 7.0.7).[22] Natural Localized Molecular Orbitals (NLMOs) and canonical molecular orbitals (CMOs) were visualized with Jmol (version 14).[23] Natural Resonance Theory (NRT) analysis was performed on ***E*-MePPCl**, ***E*-MePPBr**, and ***E*-MePPI** (DKH-PBE0/old-DKH-TZVPP//PBE0-D3/def2-TZVPP). ^{31}P NMR spectroscopic properties were calculated for ***E*-8*** and ***Z*-8*** in ORCA 6.1.0, employing the PBE0 functional, Grimme's D4 dispersion correction, the pcsseg-2 basis set for all atoms, the TightSCF command to tighten self-consistent field convergence

criteria, DEFGRID3 numerical integration, and the restricted Kohn-Sham formalism (RKS).[16, 24-32].

Synthesis of (M^{s}FluInd*)Br (1). Dimethyl 5-bromoisophthalate,[33] 1-bromo-3,5-bis(1-hydroxy-1-methylethyl)benzene,[34] 1-bromo-3,5-bis(1-chloro-1-methylethyl)benzene,[35] 2,5-dichloro-2,5-dimethyl-hexane[36], octamethyloctahydrodibenzofluorene, the ketonic (M^{s}FluInd*)Br precursor (**a**),[1] the olefinic (M^{s}FluInd*)Br precursor (**b**),[1] and (M^{s}FluInd*)Br (**1**)[1] were prepared following established literature protocols following the overall scheme below (Figure S1).

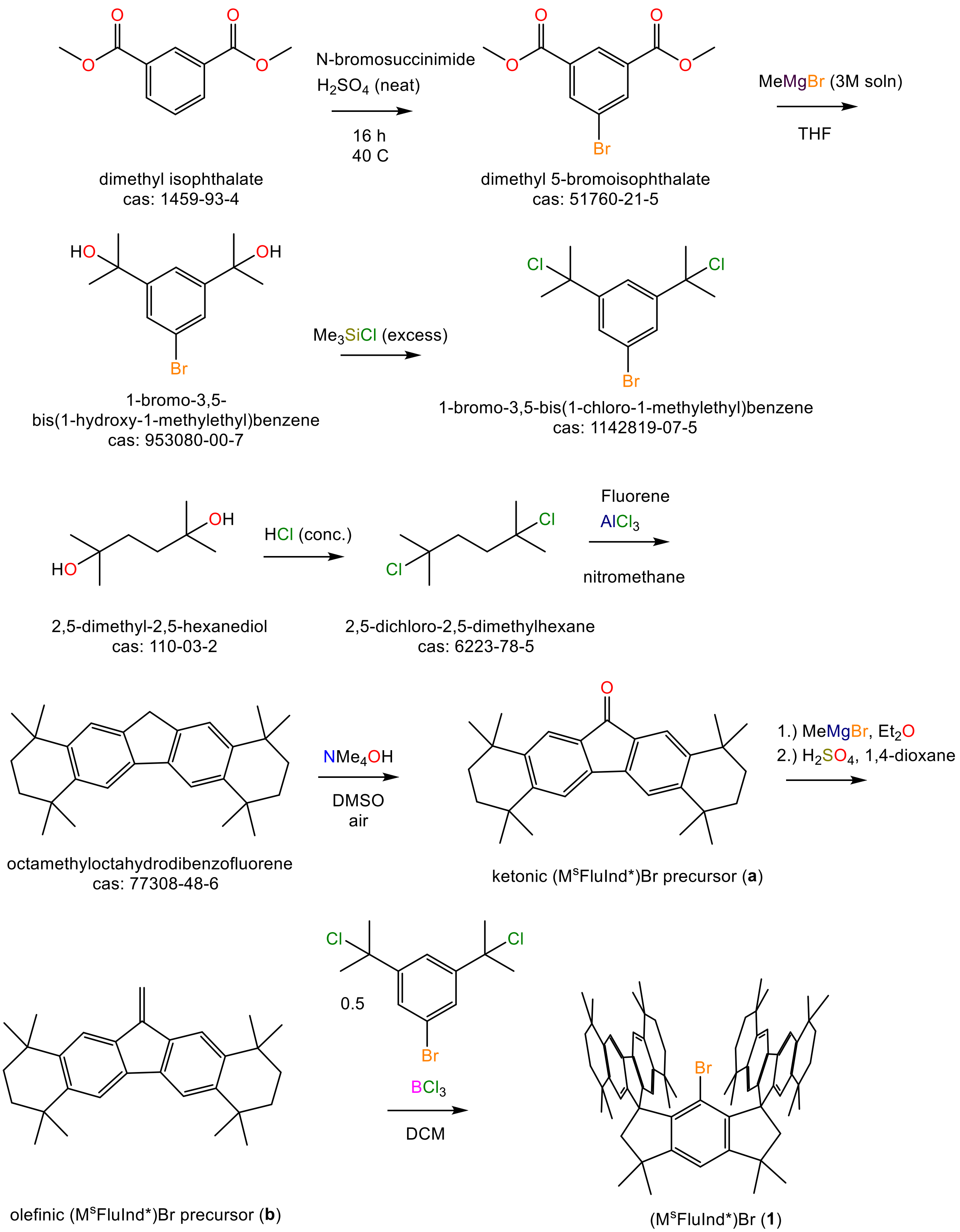

Figure S1. Synthetic route to ($M^sFluInd^*$)Br (**1**).

2. Synthesis and characterization of novel compounds.

2.1 Synthesis of ($M^sFluInd^*$)PCl_2•(hexane) (3•(hexane)).

A solution of *tert*-butyl lithium (1.7 M in pentane, 2.3 mL, 4 mmol) was added to a yellow suspension of **1** (0.800 g, 0.77 mmol) in Et_2O (5 mL) at –78 °C. The yellow solution darkened and was stirred for 15 min before being allowed to warm up to room temperature. As the mixture warmed, the solution reddened and a colorless precipitate formed. The reaction mixture was stirred for 1 h at room temperature before the solvent was removed under reduced pressure to afford a red solid. The solids were washed with Et_2O (3 × 6 mL) before being resuspended in Et_2O (4 mL) and cooled to –78 °C. The colorless suspension was treated with PCl_3 (68 µL, 0.77 mmol) and stirred for 15 min before being warmed to room temperature and stirred for an additional 2 h. The volatiles were then removed under reduced pressure, and the remaining colorless solid was dissolved in hexane, filtered, and recrystallized from hexane before being dried under vacuum. Yield: 519 mg (59%). Crystals suitable for X-ray diffraction were grown by slow evaporation of hexane at room temperature.

Elemental analysis, Found: C, 81.99; H, 9.24%. **Calc.** for $C_{78}H_{103}Cl_2P$: C, 82.00; H, 9.09%.

^{1}H NMR (400 MHz, C_6D_6): δ = 7.87 (s, 4H), 7.54 (s, 1H), 7.36 (s, 4H), 2.54 (s, 4H), 1.71-1.51 (m, 28H), 1.35 (s, 12H), 1.29 (s. 24H), 1.19 (s, 12H) ppm.

^{13}C{^{1}H} NMR (101 MHz, C_6D_6): δ = 144.0, 138.2, 122.9, 122.3, 117.9, 64.7, 60.2, 42.9, 35.7, 34.9, 34.7, 34.5, 32.9, 32.7, 32.5, 32.3 ppm.

^{31}P NMR (162 MHz, C_6D_6): δ = 159.6 (s) ppm.

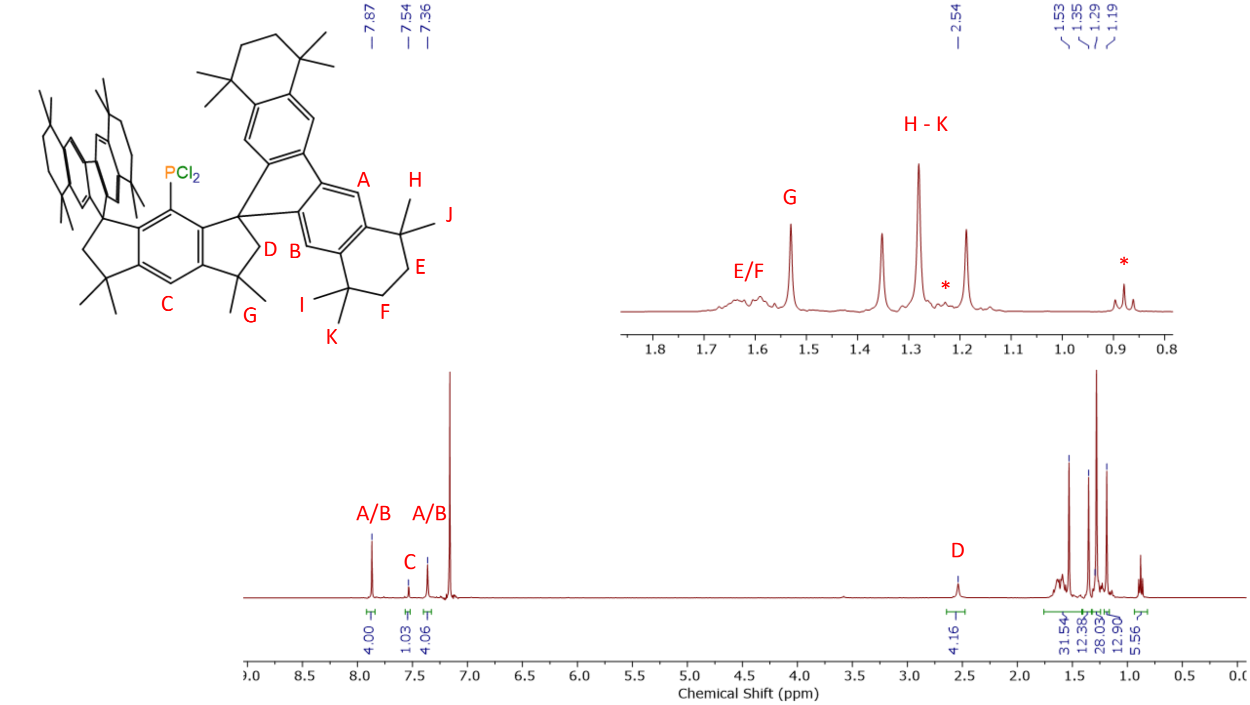

Figure S2. ^{1}H NMR spectrum (C_6D_6, 400 MHz) of **3**•(hexane) at room temperature. The asterisk denotes resonances from solvated hexane.

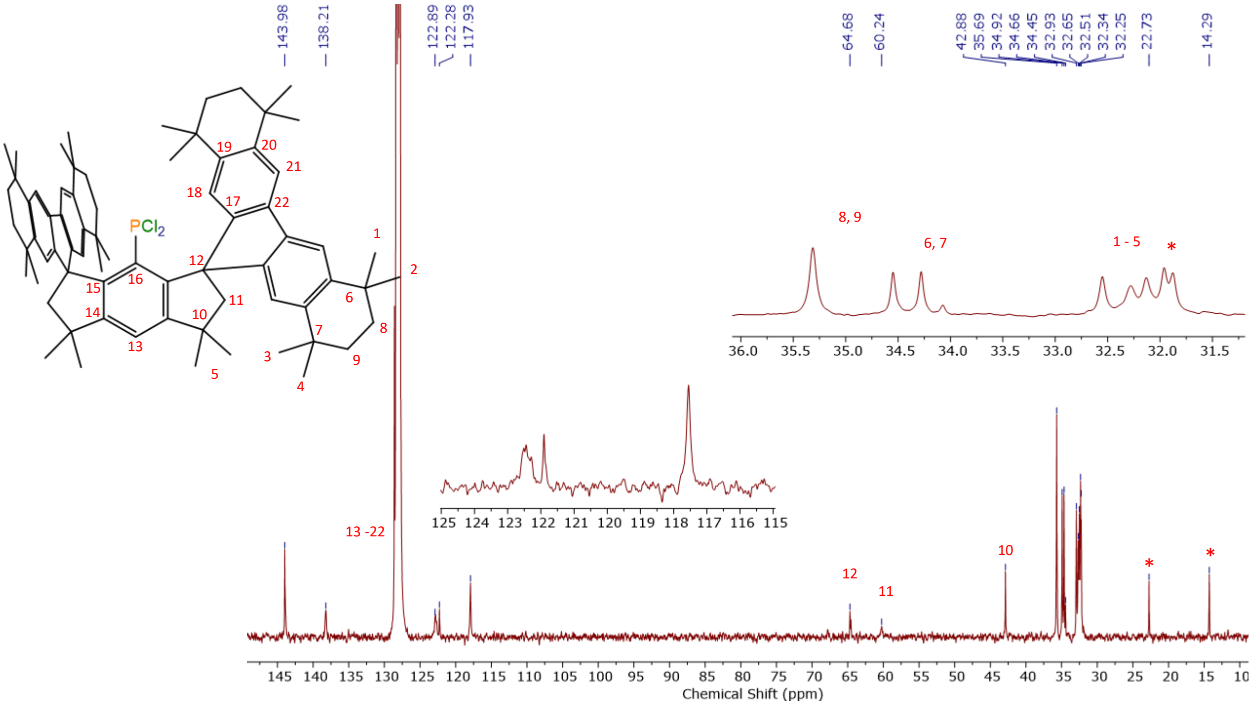

Figure S3. $^{13}C\{^{1}H\}$ NMR spectrum (C_6D_6, 101 MHz) **3**•(hexane) at room temperature. The asterisks denotes a signal from the hexane solvate.

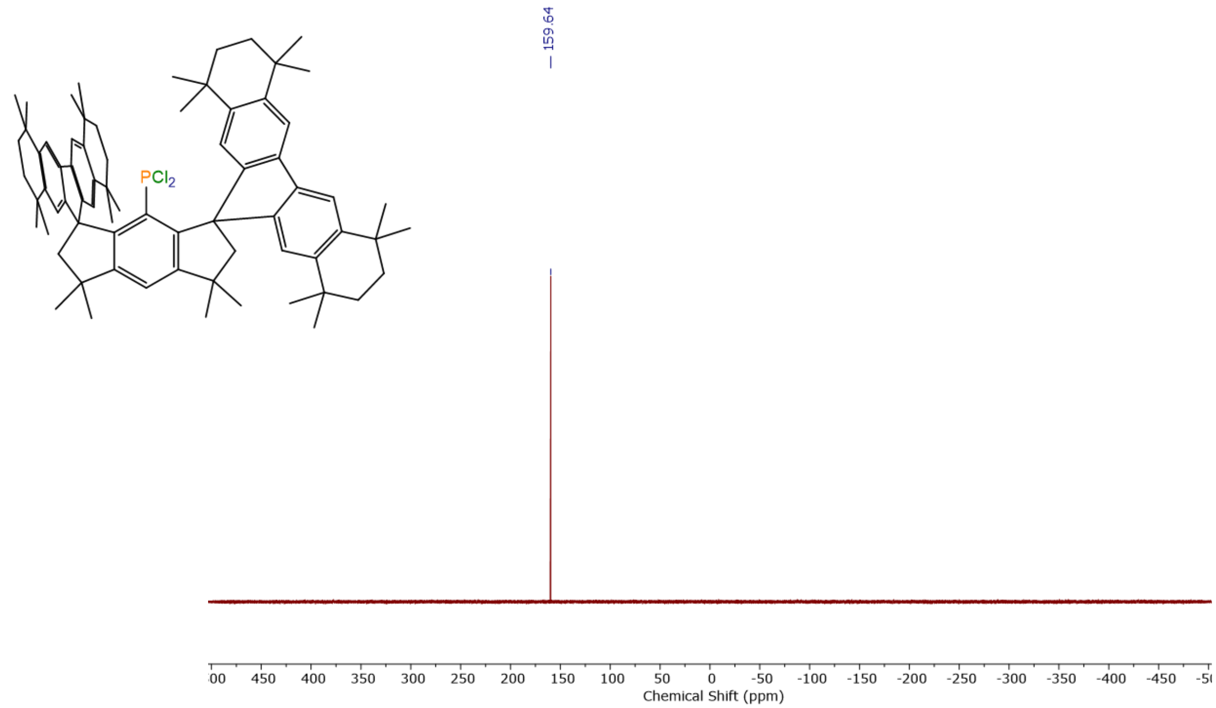

Figure S4. $^{31}P\{^{1}H\}$ NMR spectrum (C_6D_6, 162 MHz) of **3**•(hexane) at room temperature.

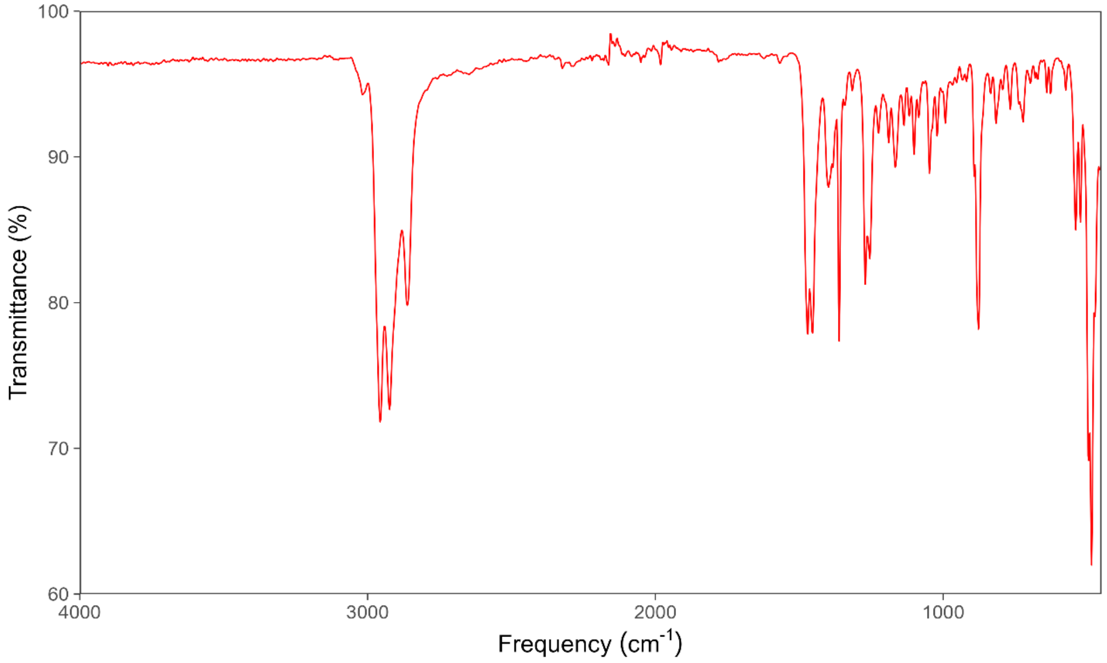

Figure S5. Experimental IR spectrum of **3**•(hexane).

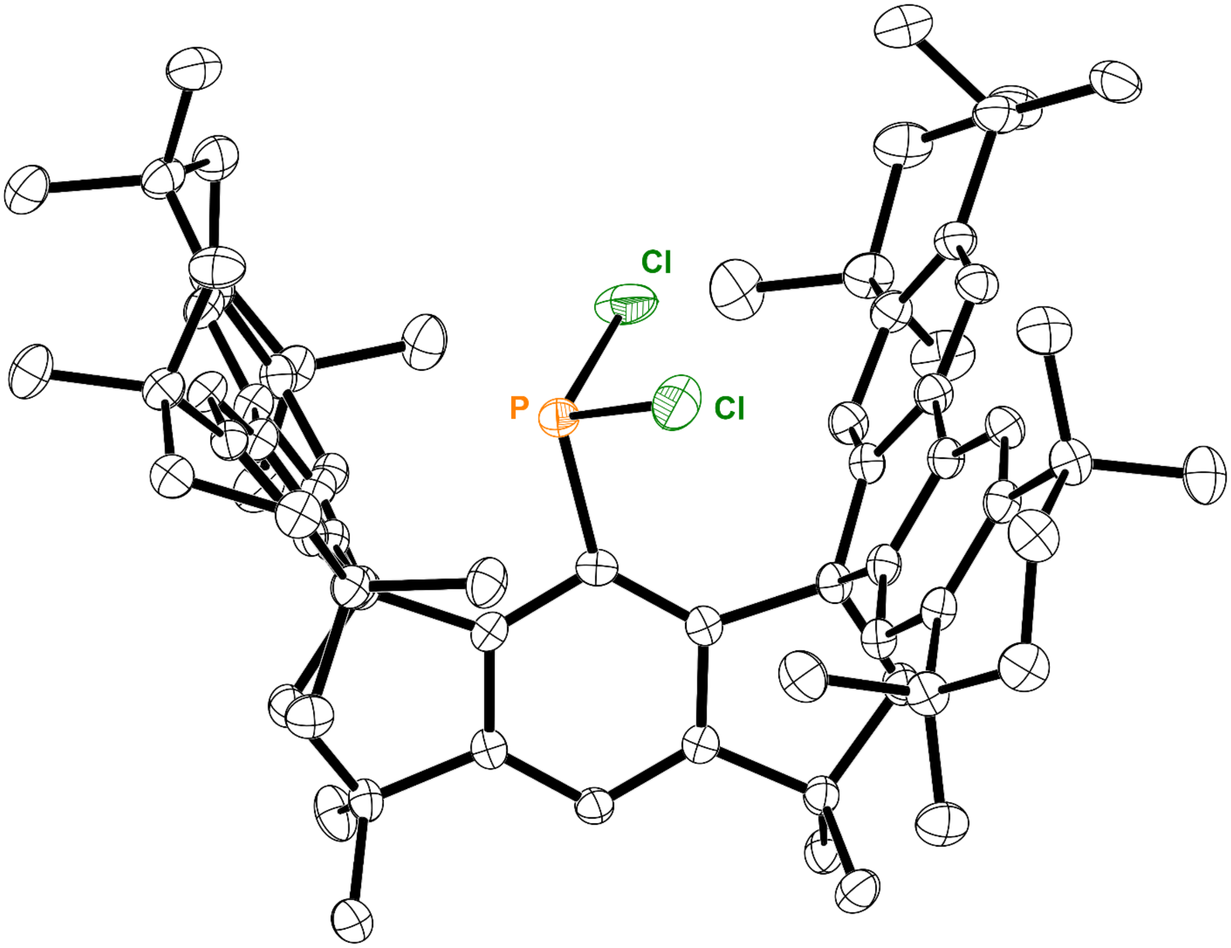

Figure S6. Thermal ellipsoid plot (50% probability) of **3**•(hexane). Solvent molecules, H atoms, and disordered components are omitted for clarity. Color code: P orange, C black, Cl dark green.

2.2 Synthesis of ($M^sFluInd^*$)PH_2•(hexane) (4•(hexane)).

Compound **4** was prepared following an adapted protocol for the preparation of (Mes*)PH_2.[37] A solution of *tert*-butyl lithium (1.7 M in pentane, 8.5 mL, 14 mmol) was added to a yellow suspension of **1** (2.99 g, 2.89 mmol) in Et_2O (20 mL) at –78 °C. The yellow solution darkened and was stirred for 15 min before being allowed to warm up to room temperature. As the mixture warmed, the solution reddened and a colorless precipitate formed. The reaction mixture was stirred for 1 h at room temperature before being cooled to –78 °C and allowed to settle. The deep red supernatant was carefully decanted via PTFE cannula into a dry ampoule under inert atmosphere, and volatiles were removed under reduced pressure. The remaining solid was suspended in fresh Et_2O (7 mL), stirred, and decanted at –78 °C for four iterations to obtain a colorless solid, free of the red impurity. The solid was stripped of volatiles under vacuum before being resuspended in Et_2O (16 mL) and cooled to –78 °C. The colorless suspension was treated with PCl_3 (250 μL, 2.86 mmol) and stirred for 15 min before being warmed to room temperature and stirred for an additional 2 h. The colorless suspension was then stripped of volatiles before being resuspended in Et_2O (20 mL). The suspension was then transferred via PTFE cannula to a suspension of $LiAlH_4$ (3.30 g, 87 mmol) in Et_2O (10 mL) at –78 °C. Additional aliquots of Et_2O (2 × 5 mL) were used for quantitative transfer. The resulting grey suspension was stirred for 1 h before being allowed to warm to room temperature and stirred overnight. The following morning, the reaction mixture was cooled to –78 °C and quenched with an aqueous solution of HCl (3.7% v/v in H_2O, 8 mL) dropwise under a backflow of argon over 2 h. *Caution! This results in the generation of heat and flammable H_2 gas with vigorous bubbling*. The mixture is then diluted in ether (400 mL) and water (300 mL). The insoluble salts were separated by vacuum filtration and extracted with additional Et_2O (3 × 100 mL). The aqueous phase was separated and extracted with additional portions of Et_2O (3 × 50 mL). The combined Et_2O phases were washed with brine (200 mL) before being dried over $MgSO_4$, filtered, and stripped of solvent to afford an oily solid. The solid was washed with MeCN and recrystallized from hexane at –30 °C before being dried under vacuum to obtain **4**•(hexane) as a crystalline solid. Yield: 2.226 g, 71%. Crystals suitable for X-ray diffraction were grown from a solution of hexane at –30 °C.

Note: *We found that it was more efficient to synthesize **4**•(hexane) from **1** without fully isolating the intermediate **3**. However, **4**•(hexane) could be made from **3** by reducing a batch of isolated **3**•(hexane) with $LiAlH_4$ following a similar protocol described above.*

Elemental analysis, Found: C, 86.69; H, 9.65%. **Calc.** for $C_{78}H_{105}P$: C, 87.26; H, 9.86%.

ESI-MS (m/z) $[4+H]^+$ 987.686 (calc. 987.693).

^{1}H NMR (400 MHz, C_6D_6): δ = 7.80 (s, 4H), 7.33 (s, 1H), 7.32 (s, 4H), 2.55 (s, 4H), 2.15 (d, $^1J_{PH}$ = 209 Hz, 2H), 1.73-1.50 (m, 28H), 1.34 (s, 12H), 1.27 (s. 12H), 1.22 (s, 12H), 1.16 (s, 12H) ppm.

^{13}C{^{1}H} NMR (101 MHz, C_6D_6): δ = 155.2, 151.5, 147.1, 147.1, 144.2, 143.8, 138.8, 122.3, 117.4, 116.2, 64.1, 59.0, 42.8, 35.7, 35.7, 34.9, 34.6, 32.9, 32.9, 32.4, 32.4, 32.1 ppm.

^{31}P NMR (162 MHz, C_6D_6): δ = –158.2 (t, $^1J_{PH}$ = 209 Hz) ppm.

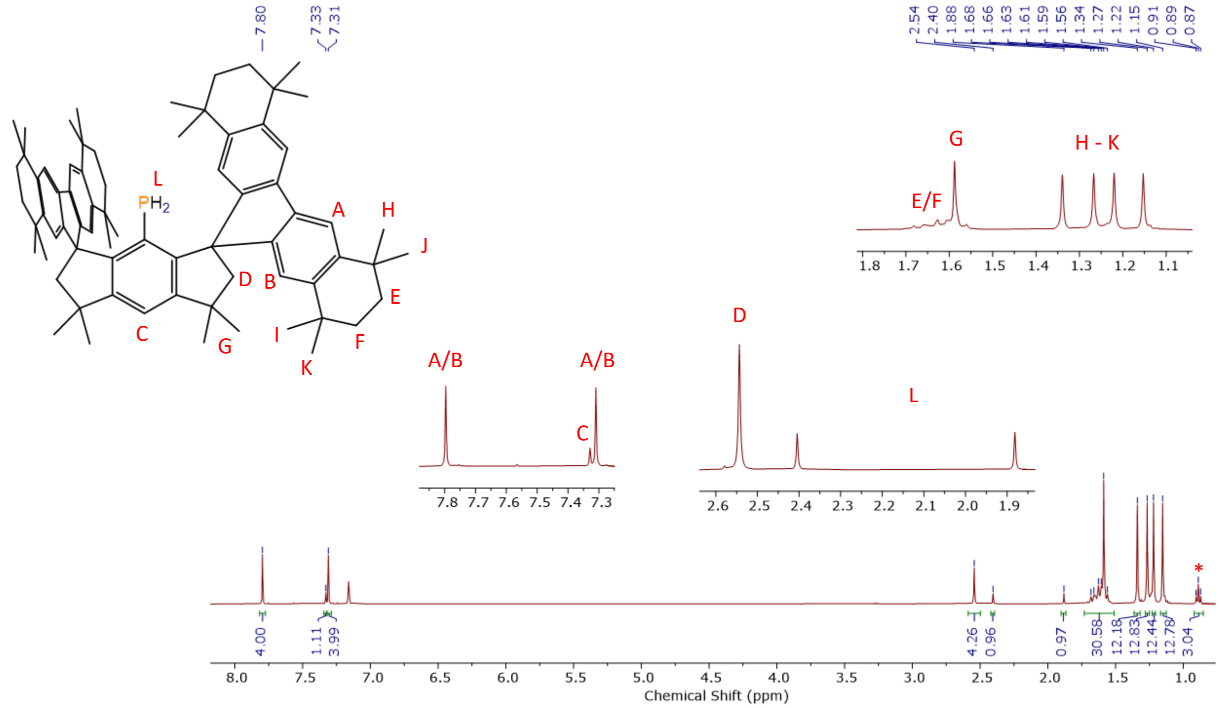

Figure S7. ^{1}H NMR spectrum (C_6D_6, 400 MHz) of **4**•(hexane) at room temperature. The asterisk denotes a signal from the hexane solvate.

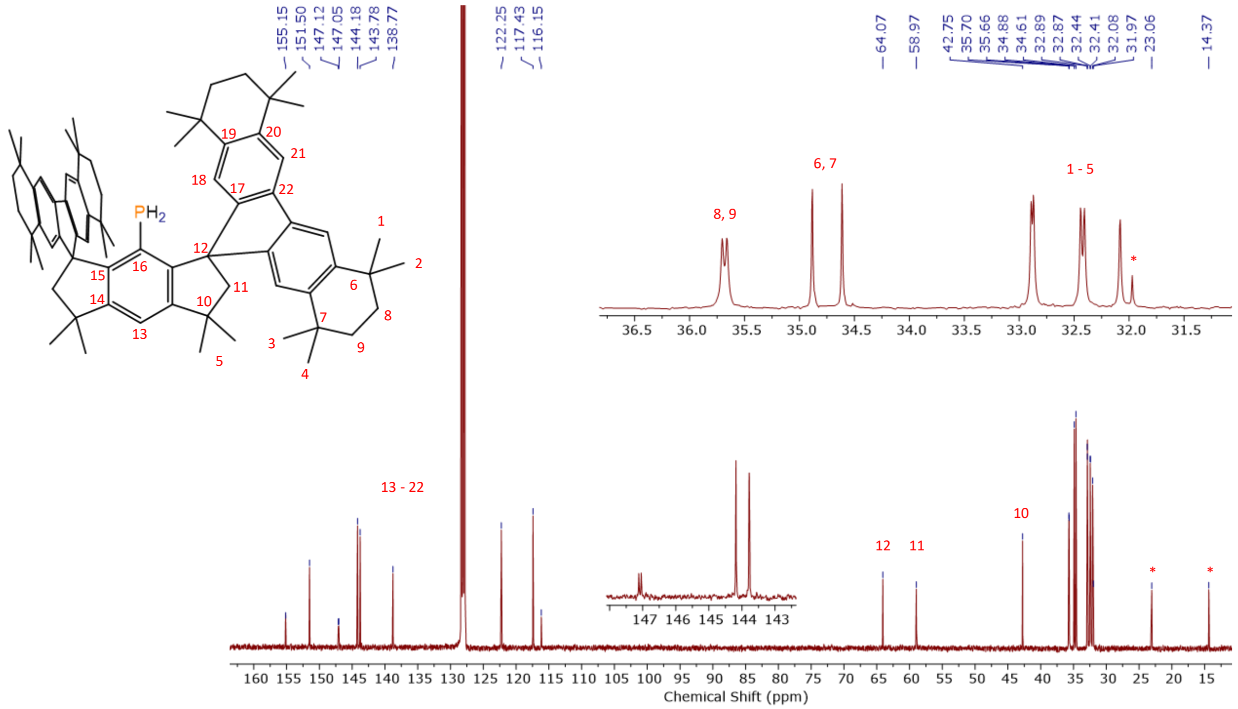

Figure S8. $^{13}C\{^1H\}$ NMR spectrum (C_6D_6, 101 MHz) of **4**•(hexane) at room temperature. The asterisks denotes a signal from the hexane solvate.

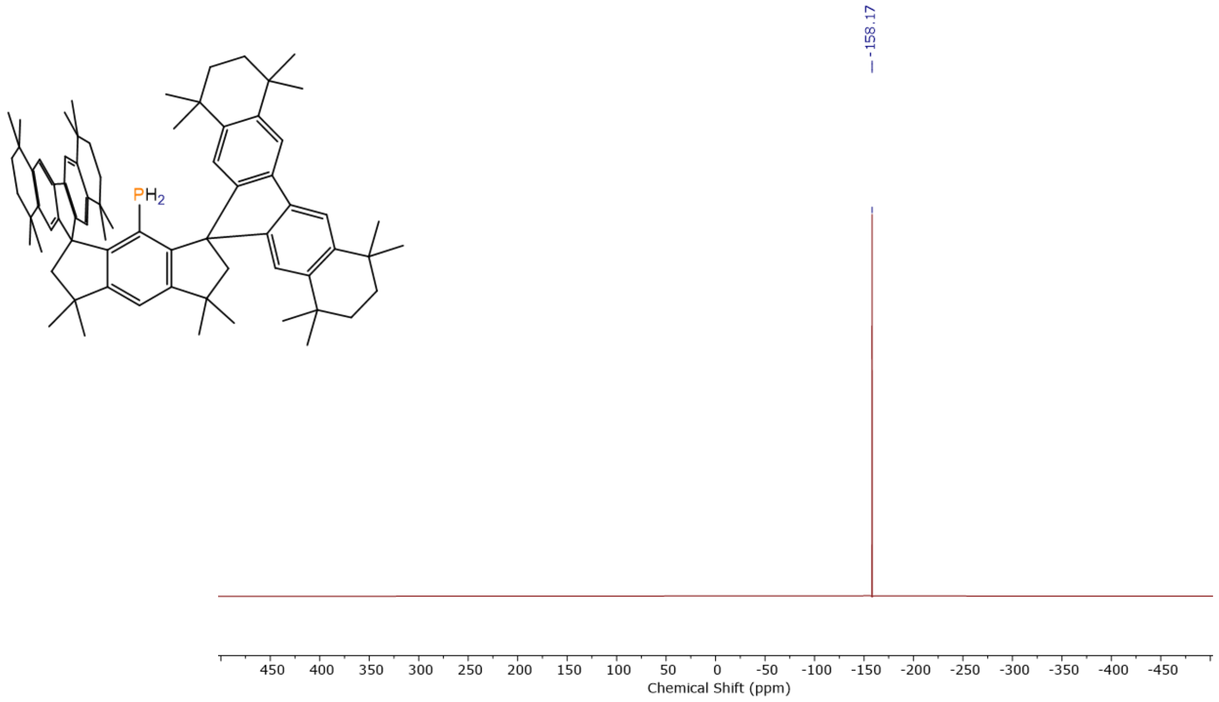

Figure S9. $^{31}P\{^1H\}$ NMR spectrum (C_6D_6, 162 MHz) of **4**•(hexane) at room temperature.

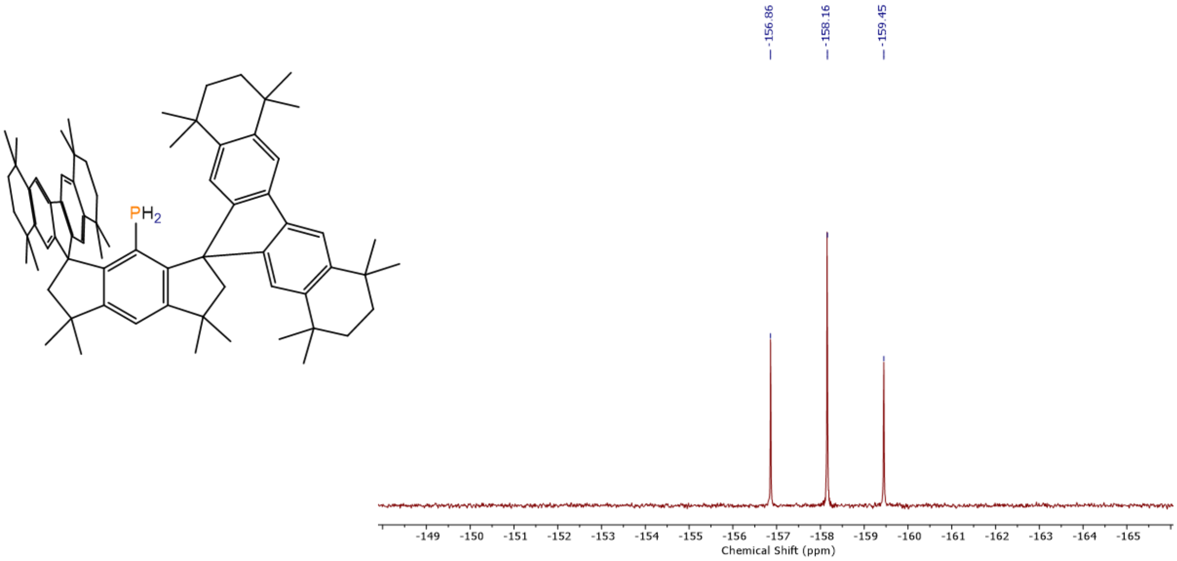

Figure S10. ^{31}P NMR spectrum (C_6D_6, 162 MHz) of **4**•(hexane) at room temperature.

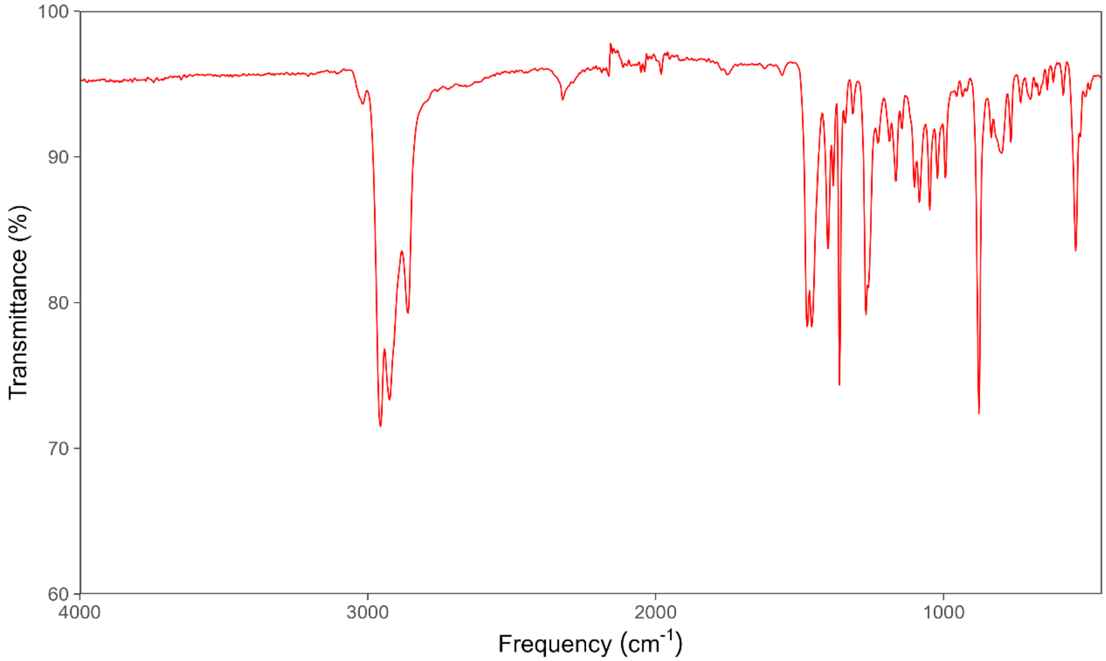

Figure S11. Experimental IR spectrum of **4**•(hexane).

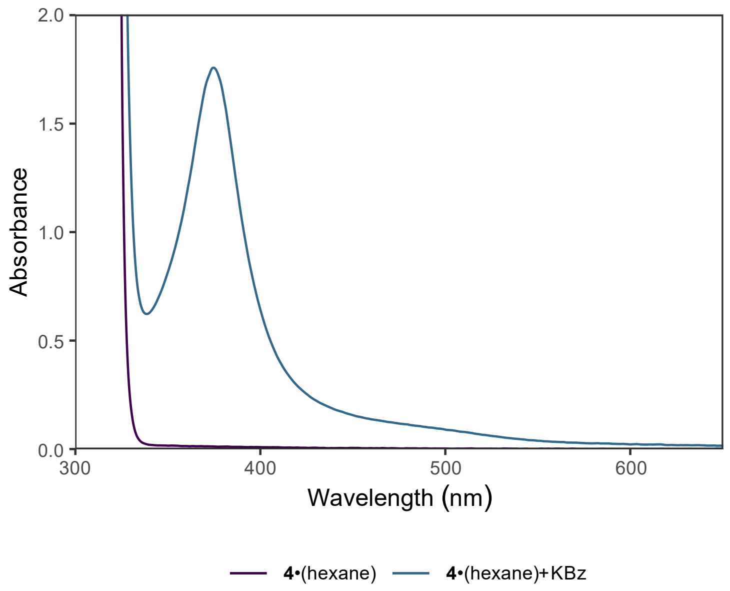

Figure S12. Experimental UV-Vis spectrum of **4**•(hexane) (140 μM) and a filtered reaction mixture of **4**•(hexane) (180 μM) with an excess of KBz (1.5 equivalents) in benzene at room temperature.

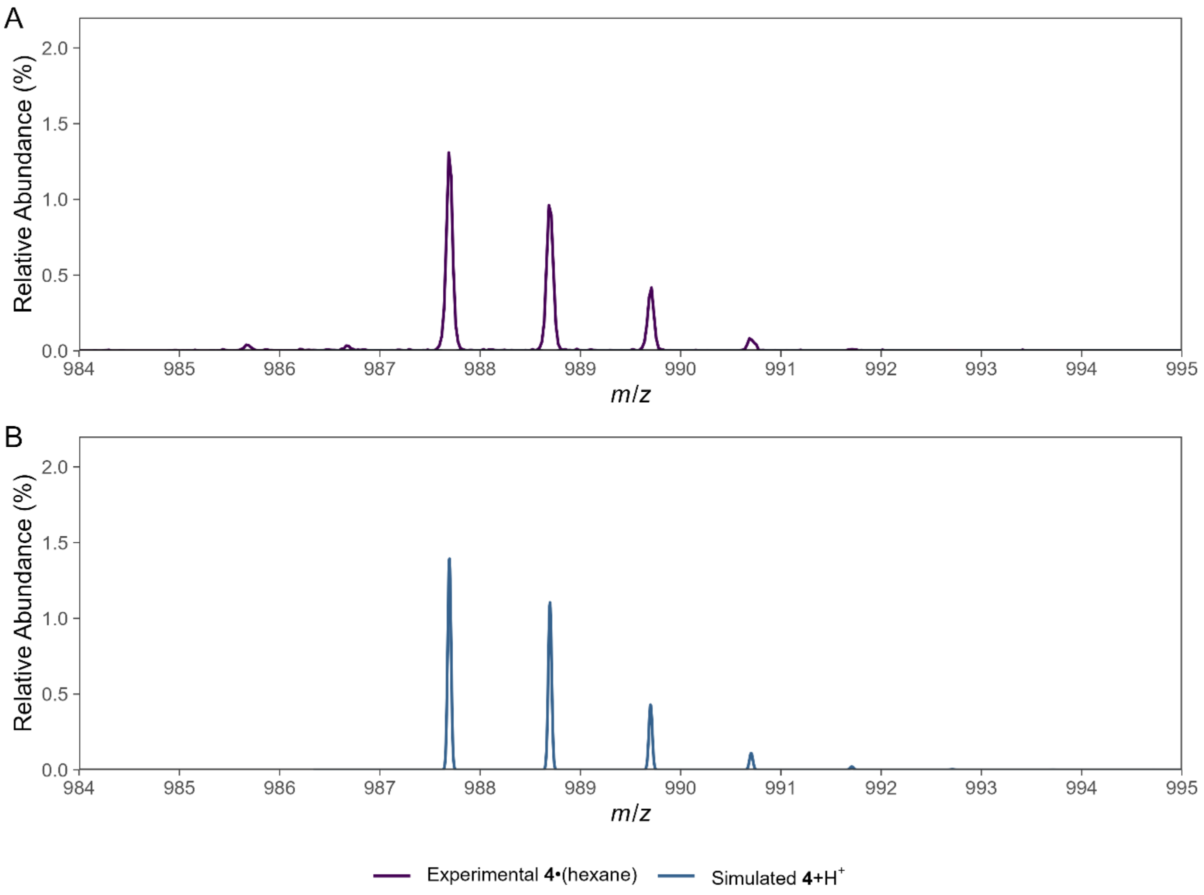

Figure S13. (A) Experimental ESI-MS spectrum for **4**•(hexane). (B) Simulated ESI-MS spectrum for **4**+H$^+$.

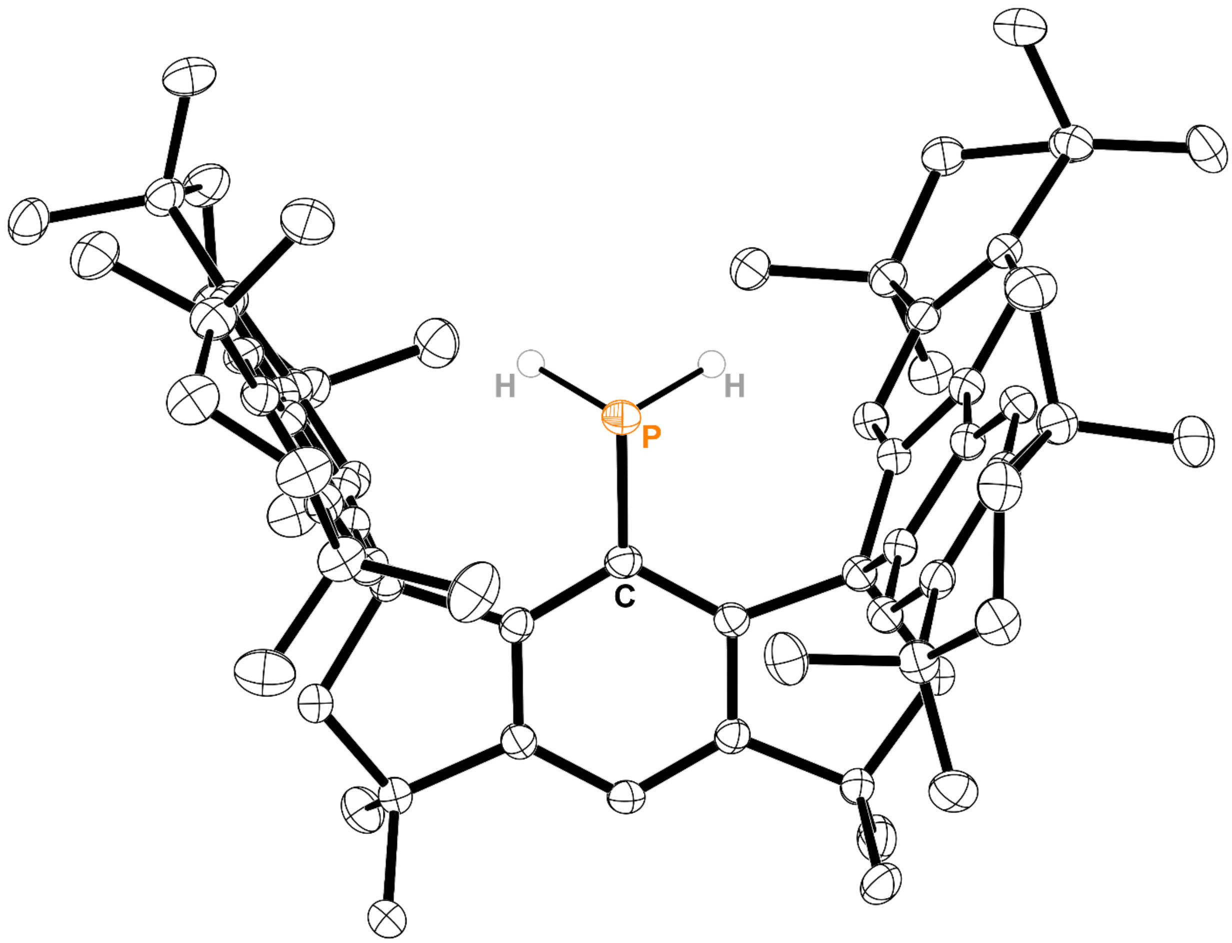

Figure S14. Thermal ellipsoid plot (50% probability) of **4**•(hexane). Solvent molecules, C-bound H atoms, and disordered components are omitted for clarity. Color code: P orange, C black, H grey.

2.3 Synthesis of (M^{s}FluInd*)PTMSH•(Et_2O)$_2$ (6•(Et_2O)$_2$)

A solution of **4**•(hexane) (1.00 g, 0.935 mmol) in benzene (12 mL) was added to a red suspension of KBz (162 mg, 1.25 mmol) in benzene (2 mL) and stirred at room temperature for 2 h. The resulting red suspension was filtered through glass filter paper, and the collected red solids were extracted with hexane (3 × 1.5 mL). The resulting dark red filtrate was treated with TMSCl (130 µL, 1.02 mmol) and stirred at room temperature for 15 min, quickly forming a yellow suspension. The solvent was stripped and the resulting residue was extracted with pentane (3 × 4 mL) and filtered through glass filter paper. The solvent was removed under reduced pressure from the resulting colorless reaction mixture, and the colorless solid recrystallized from Et_2O at –30 °C Yield: 0.727 g, 64%.

Elemental analysis, Found: C, 82.72; H, 9.76%. Calc. for $C_{83}H_{119}PO_2Si$: C, 82.53; H, 9.93%.

^{1}H NMR (400 MHz, C_6D_6): δ = 7.88 (s, 4H), 7.57-7.24 (m, 5H), 2.41 (d, $^1J_{PH}$ = 221 Hz, 1H), 2.65-2.25 (br m, 4H), 1.80-1.18 (br m, 78 H), –0.73 (d, $^3J_{PH}$ = 3.7 Hz, 9H) ppm.

^{13}C{^{1}H} NMR (101 MHz, C_6D_6): δ = 164.4, 156.0, 143.7, 138.7, 132.5, 123.7, 117.7, 116.4, 65.1, 60.9, 42.0, 35.8, 35.7, 35.0, 34.7, 32.8, 32.6, 32.5, 32.2, 1.5 ppm.

^{31}P{^{1}H} NMR (162 MHz, C_6D_6): δ = –150.0 (s, satellite d, $^1J_{PSi}$ = 29.8 Hz) ppm.

^{31}P NMR (162 MHz, C_6D_6): δ = –150.0 (d, $^1J_{PH}$ = 222 Hz) ppm.

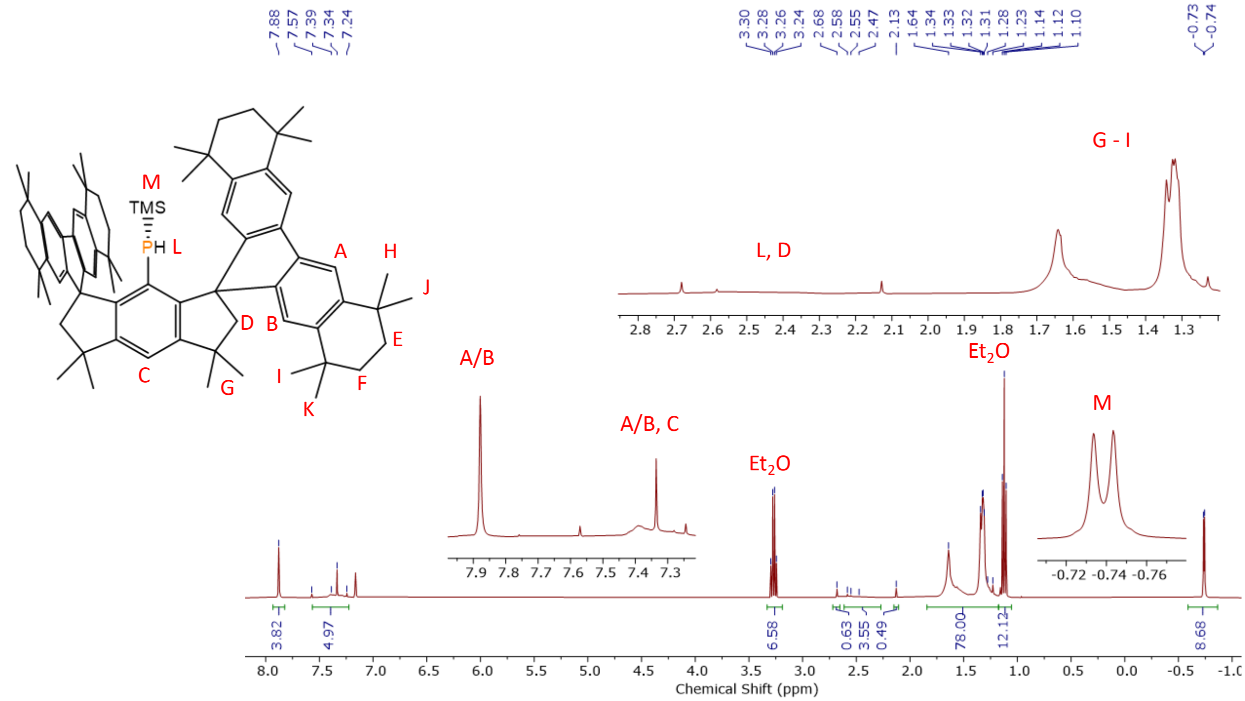

Figure S15. ^{1}H NMR spectrum (C_6D_6, 400 MHz) of **6**•$(Et_2O)_2$ at room temperature.

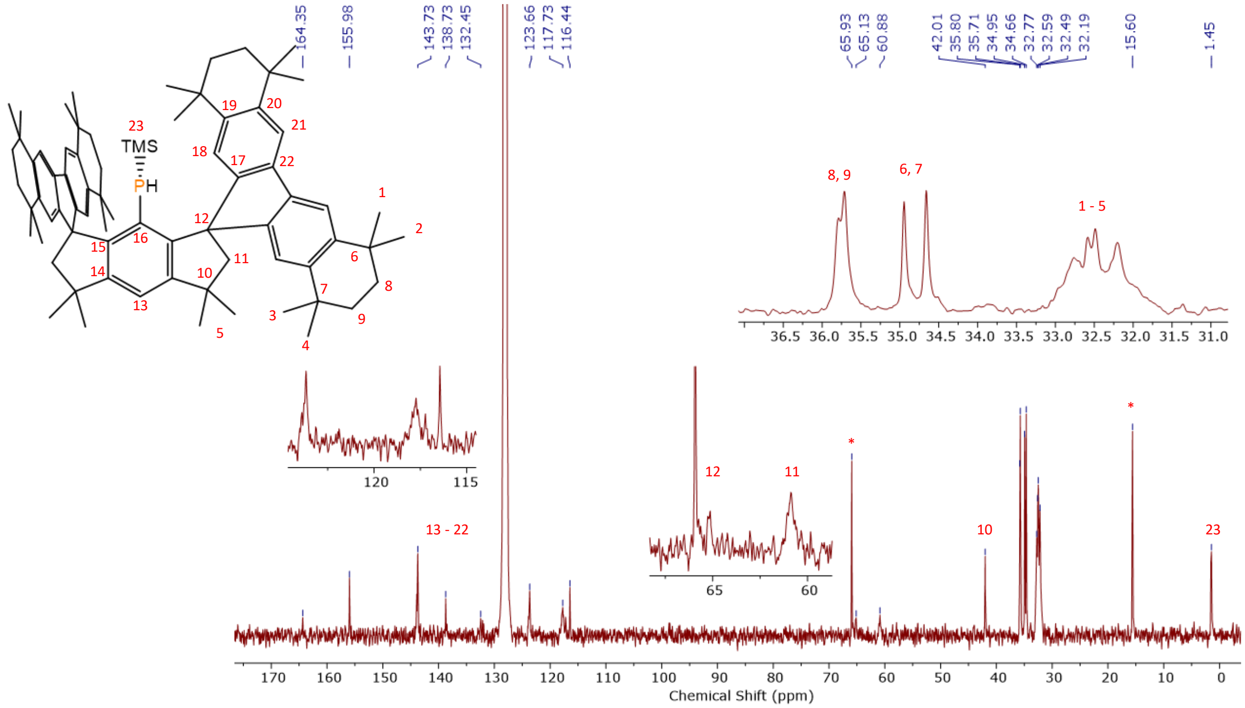

Figure S16. $^{13}C\{^{1}H\}$ NMR spectrum (C_6D_6, 101 MHz) of **6**•$(Et_2O)_2$ at room temperature. The asterisks denotes a signal from the Et_2O solvate.

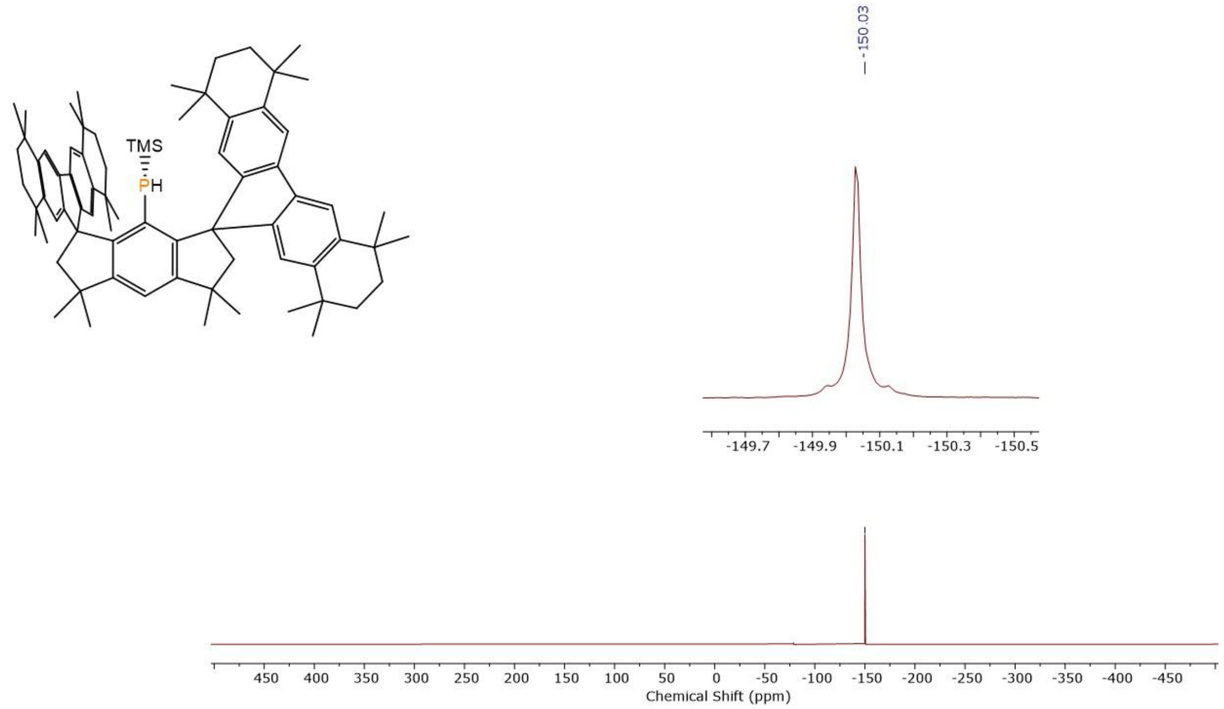

Figure S17. $^{31}P\{^1H\}$ NMR spectrum (C_6D_6, 162 MHz) of **6**•$(Et_2O)_2$ at room temperature.

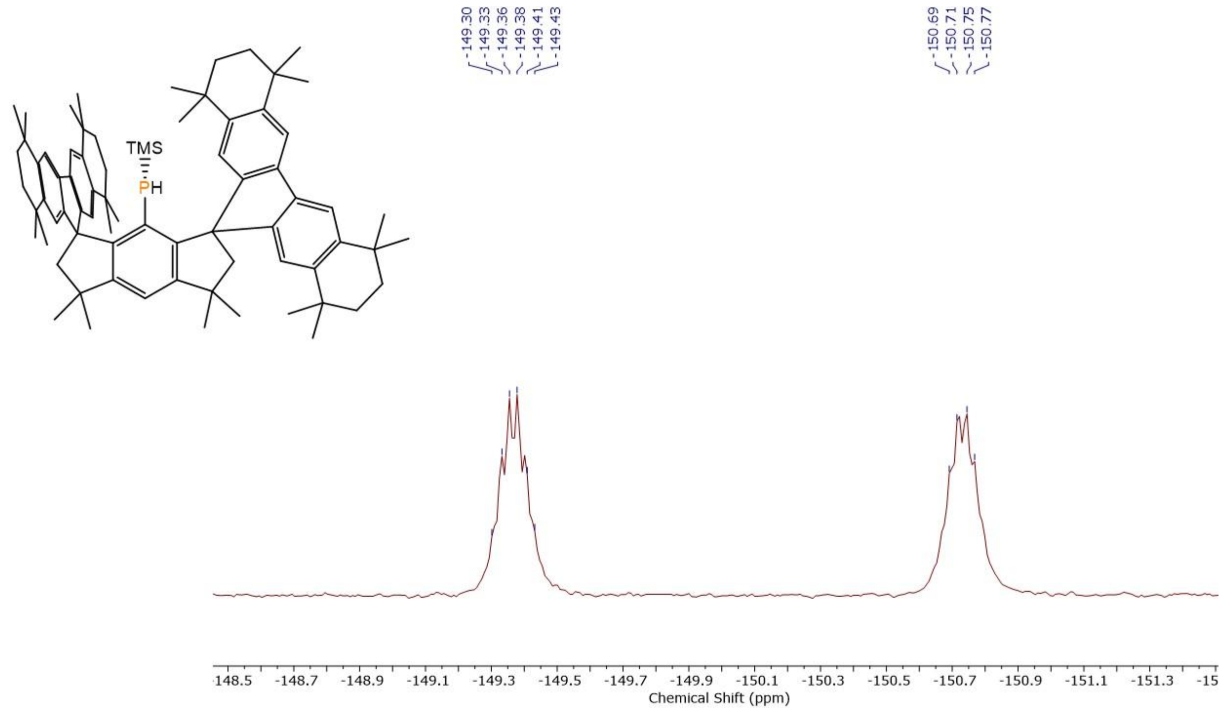

Figure S18. ^{31}P NMR spectrum (C_6D_6, 162 MHz) of **6**•$(Et_2O)_2$ at room temperature.

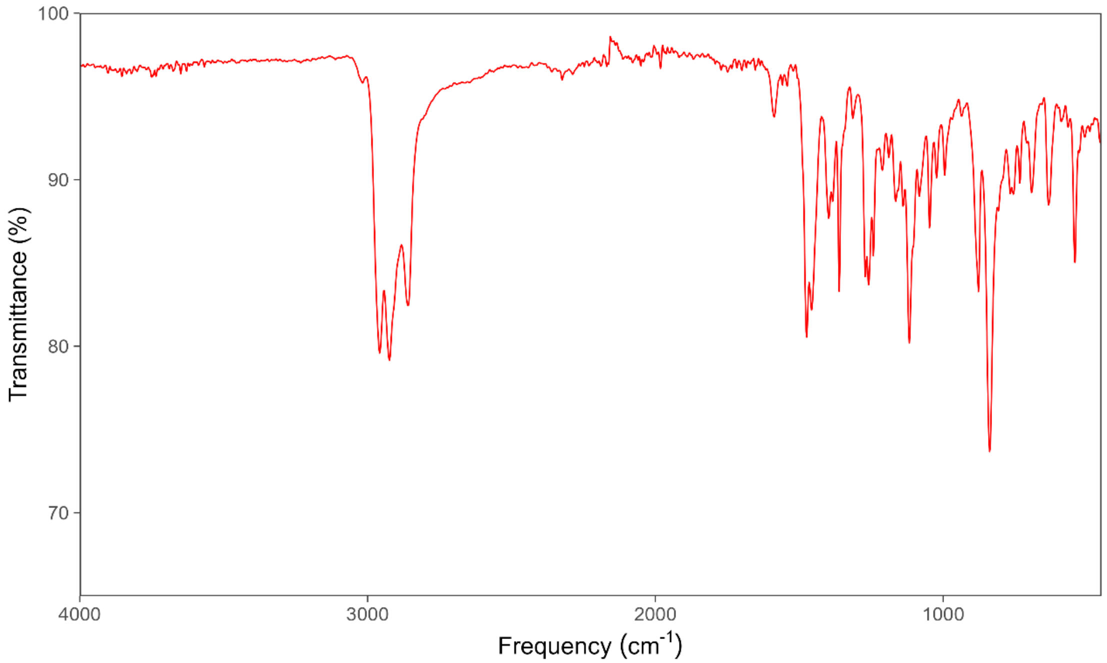

Figure S19. Experimental IR spectrum of **6**•$(Et_2O)_2$.

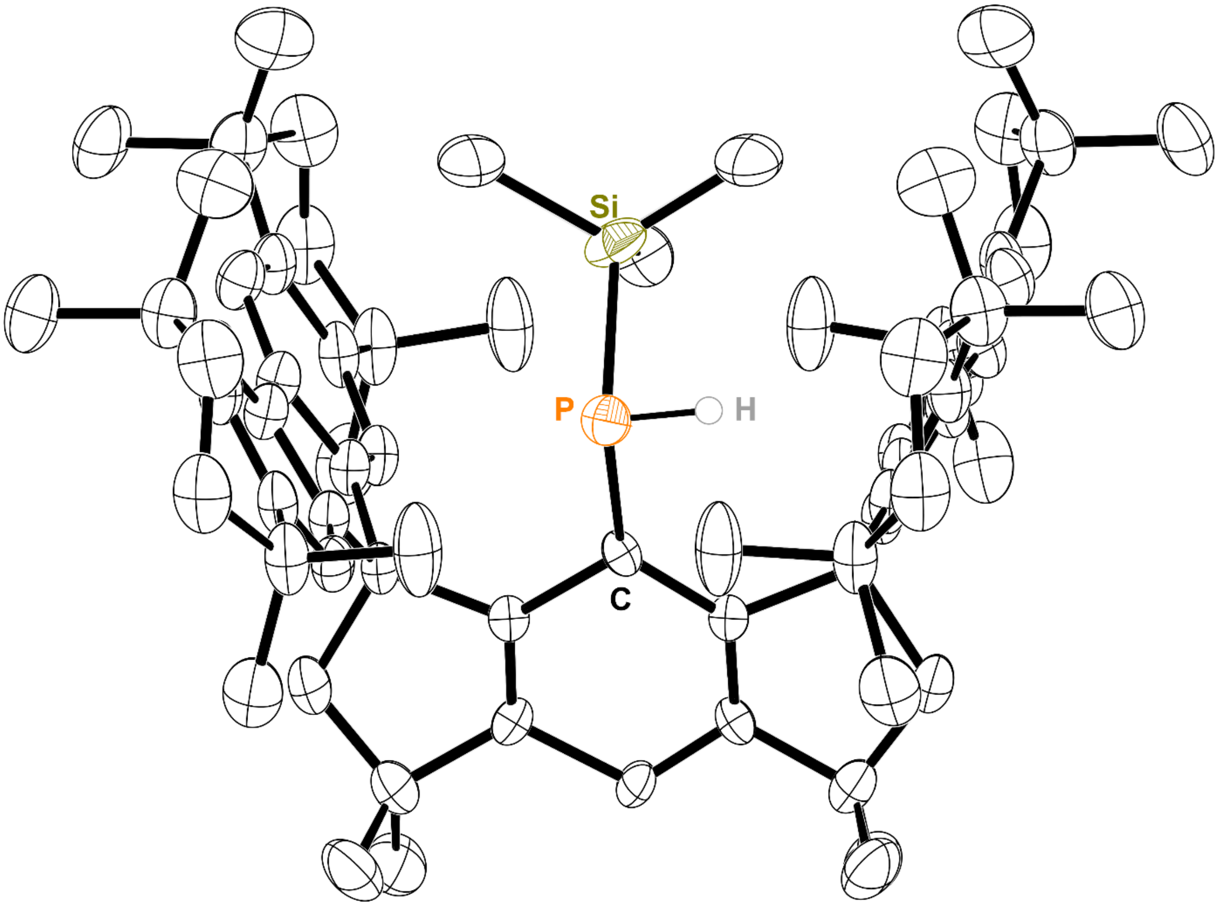

Figure S20. Thermal ellipsoid plot (50% probability) of **6**•$(Et_2O)_2$. Solvent molecules, C-bound H atoms, and disordered components are omitted for clarity. Color code: P orange, C black, H grey, Si dark yellow.

2.4 Synthesis of ($M^sFluInd^*$)$PHPCl_2$•(Et_2O)$_2$ (7•(Et_2O)$_2$)

A solution of **4**•(hexane) (249 mg, 0.232 mmol) in benzene (6 mL) was added to a red suspension of KBz (82 mg, 0.63 mmol) in benzene (2 mL) and stirred at room temperature for 2 h. The resulting red suspension was filtered through glass filter paper, and the collected red solids were extracted with hexane (3 × 1.5 mL). The resulting red filtrate was stripped of volatiles under reduced pressure and suspended in Et_2O (8 mL). The red suspension was cooled to –78 °C and stirred. PCl_3 (35 µL, 0.40 mmol) was added to the reaction mixture resulting in the loss of color and dissolution of solids. The reaction mixture was stirred at –78 °C for 30 min before being sonicated and allowed to warm to room temperature. As the solution warmed, a significant amount of colorless solid precipitated. After stirring at room temperature for 1 h, the volatiles were removed, and the resulting solids were extracted with portions of benzene (3 × 1.5 mL) that were passed through glass filter paper before being stripped of solvent. The product was recrystallized from Et_2O to afford the product as a colorless crystalline solid. Yield: 225 mg (78%). Crystals of **6**•(Et_2O)$_2$ suitable for X-ray diffraction were grown from ether at –30 °C.

Elemental analysis, Found: C, 80.00; H, 8.92%. **Calc.** for $C_{80}H_{110}P_2Cl_2O_2$: C, 77.70; H, 8.97%. Elemental analysis for (**7**•(Et_2O)$_2$) was unsuccessful (*vide infra*), and the best results are included.

ESI-MS (m/z) [4+H]$^+$ 987.685 (calc 987.693); only the protonolysis product **4** was able to be assigned in the ESI-MS spectrum

^{1}H NMR (400 MHz, C_6D_6): δ = 7.86 (s, 2H), 7.85 (s, 2H), 7.42 (s, 1H), 7.31 (s, 2H), 7.27 (s, 2H), 3.95 (dd, $^1J_{PH}$ = 219 Hz, $^2J_{PH}$ = 14.5 Hz, 1H), 2.59-2.44 (m, 4H), 1.72-1.49 (m, 28 H), 1.34-1.22 (m, 48 H) ppm.

^{13}C{^{1}H} NMR (101 MHz, C_6D_6): δ = 156.6, 153.1, 152.7, 152.2, 144.8, 144.8, 144.7, 143.8, 138.5, 138.4, 122.6, 122.3, 121.9, 119.4, 118.5, 118.4, 117.2, 64.4, 60.1, 42.4, 35.6, 34.9, 34.9, 34.9, 34.7, 34.5, 32.9, 32.8, 32.8, 32.7, 32.6, 32.4, 32.4, 32.3, 32.3, 32.2, 32.0 ppm.

^{31}P{^{1}H} NMR (162 MHz, C_6D_6): δ = 208.3 (d, $^1J_{PP}$ = 248 Hz), –43.0 (d, $^1J_{PP}$ = 248 Hz) ppm.

^{31}P NMR (162 MHz, C_6D_6): δ = 208.3 (dd, $^1J_{PP}$ = 248 Hz, $^2J_{PH}$ = 14.5 Hz), –43.0 (d, $^1J_{PP}$ = 248 Hz, $^1J_{PH}$ = 219 Hz) ppm.

*Bulk samples of **7**•$(Et_2O)_2$ prepared in this manner contained impurities which appeared in relatively low abundance by ^{1}H and ^{31}P{^{1}H} NMR. Attempts to purify via recrystallization were unsuccessful. We attribute our failure to remove these impurities to the high crystallinity and similar solubilities of species in the reaction mixture which contain the M^{s}FluInd* ligand. Therefore, we provide full spectral data for the bulk crystalline material isolated and confirm homogeneity of the crystalline solid by PXRD to confirm our accurate characterization of the bulk material. We reference other reports which have described similar challenges with species containing M^{s}FluInd*, other bulky ligands, and halogen-functionalized phosphanes.*[38-40]

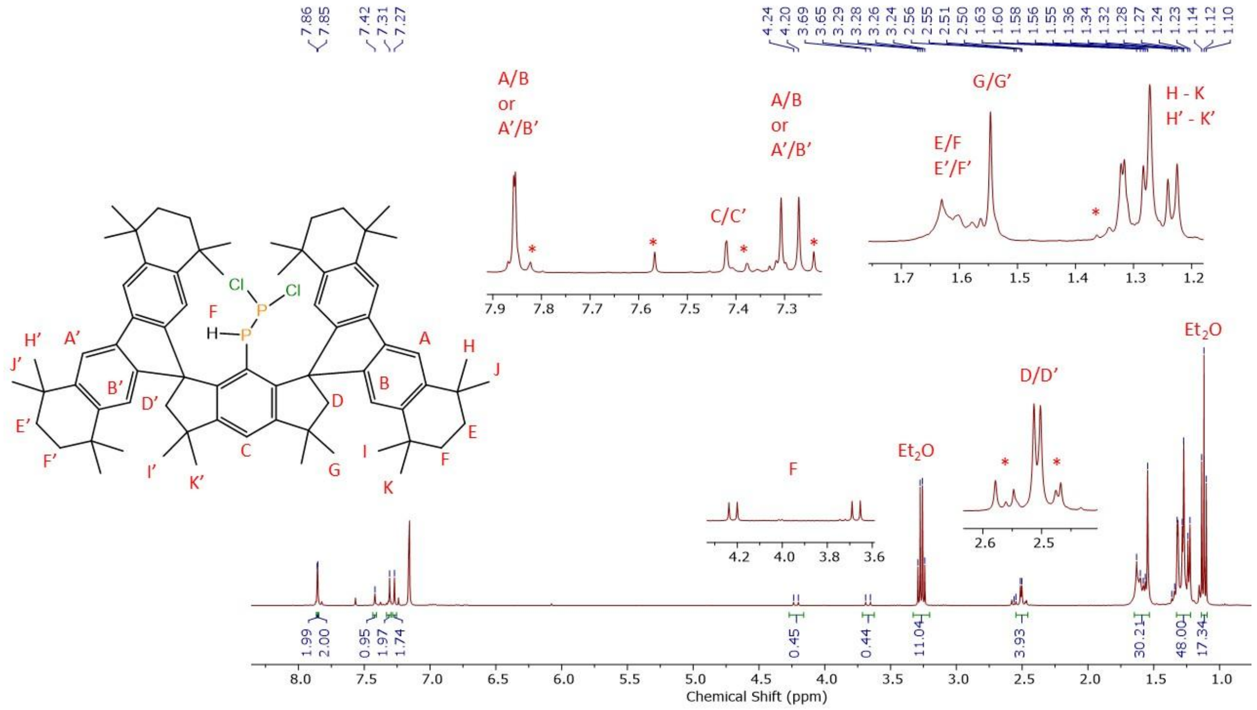

Figure S21. ^{1}H NMR spectrum (C_6D_6, 400 MHz) of **7**•$(Et_2O)_2$ at room temperature. An asterisk indicates a signal arising from impurities.

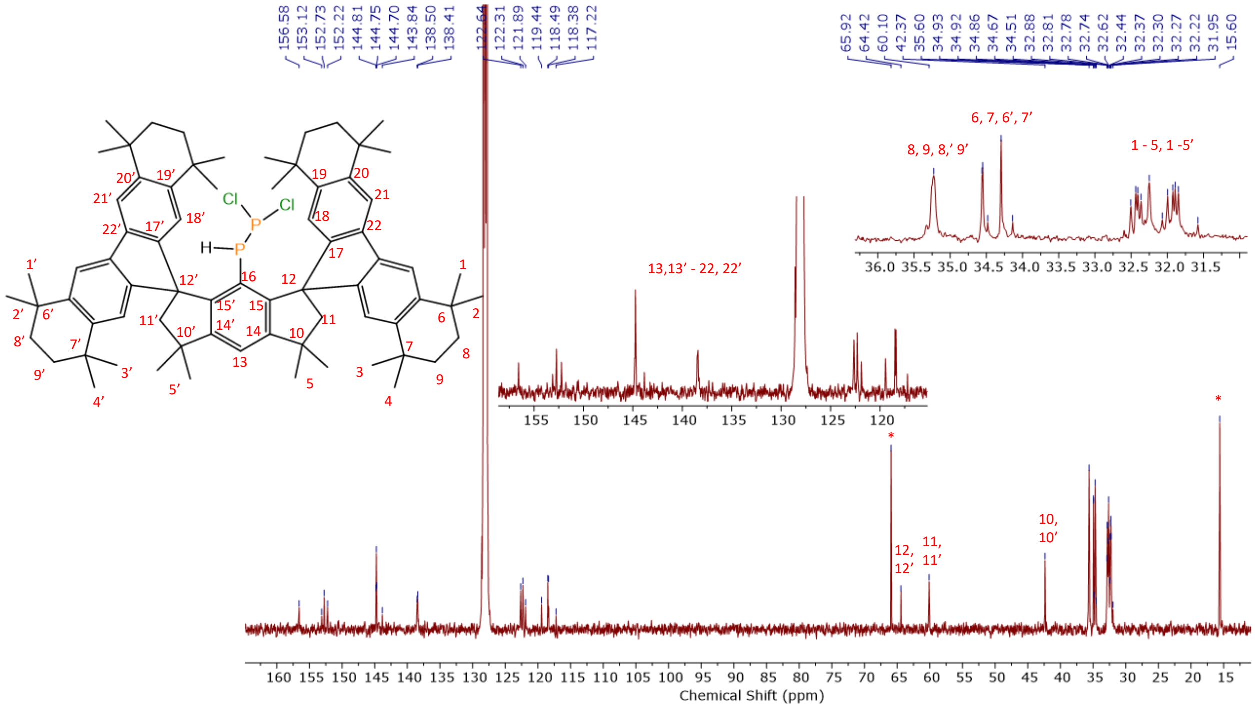

Figure S22. $^{13}C\{^1H\}$ NMR spectrum (C_6D_6, 101 MHz) of **7**•$(Et_2O)_2$ at room temperature. The asterisks denotes a signal from the Et_2O solvate.

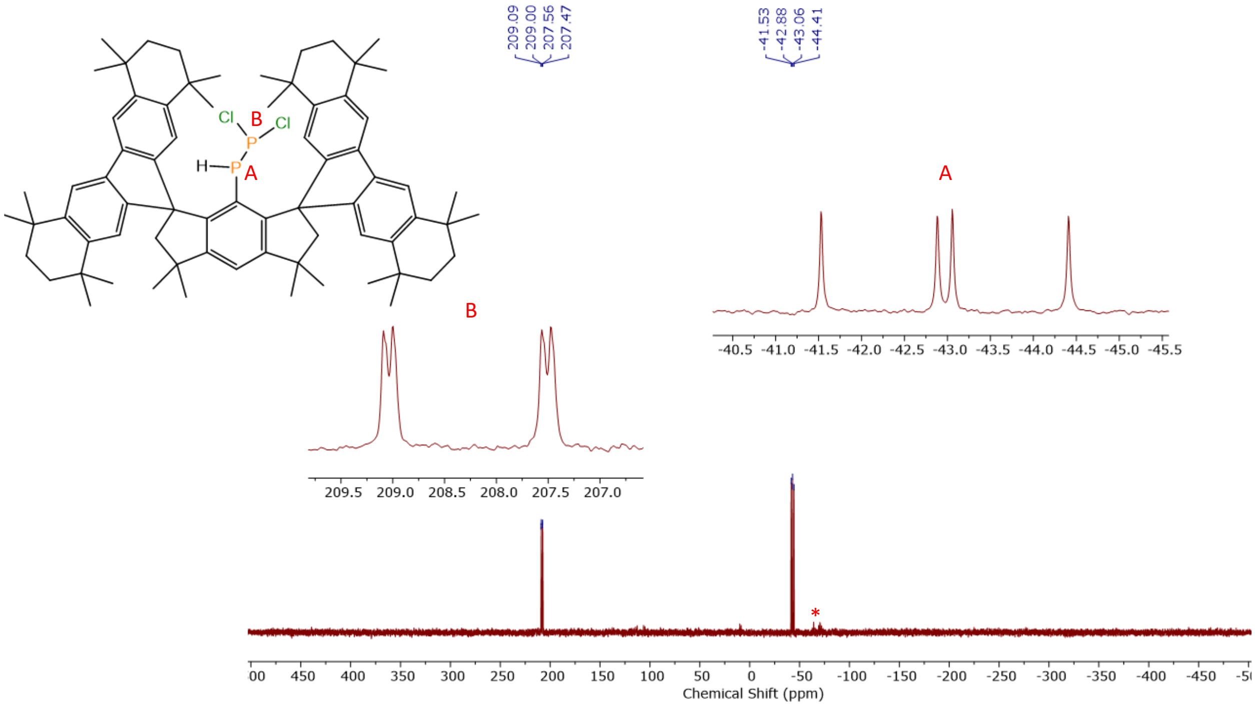

Figure S23. ^{31}P NMR spectrum (C_6D_6, 162 MHz) of **7**•$(Et_2O)_2$ at room temperature. An asterisk indicates a signal arising from impurity.

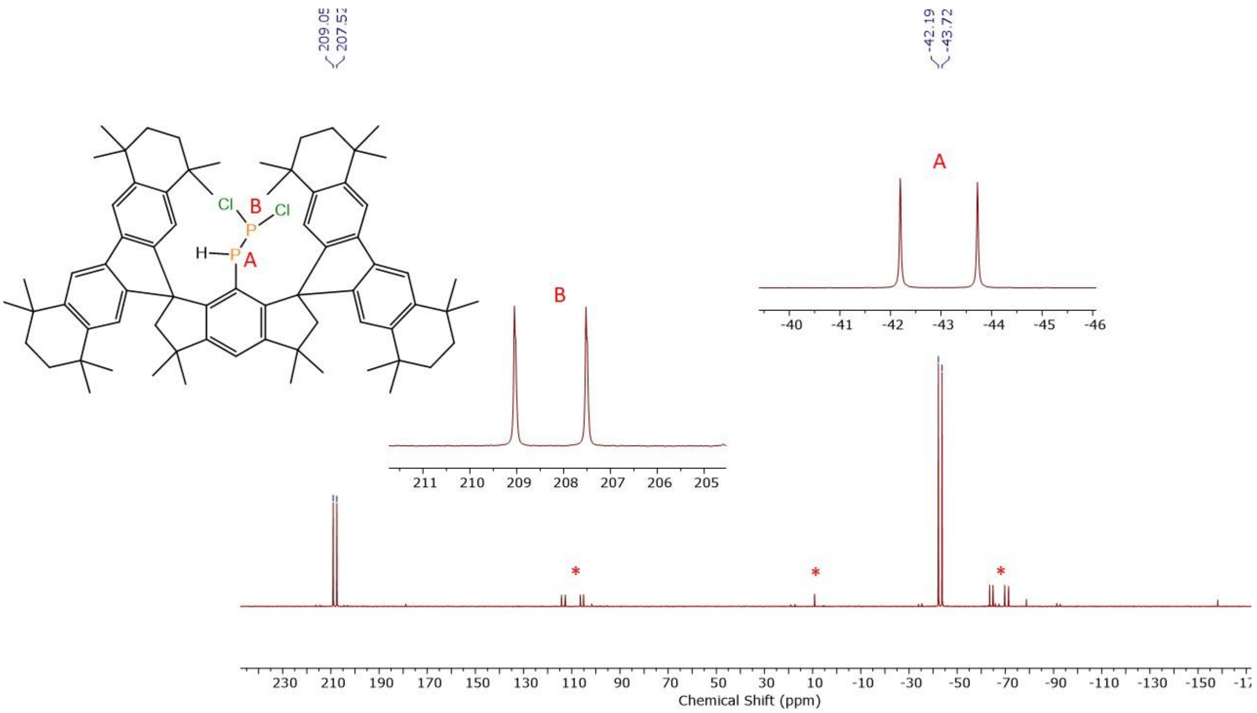

Figure S24. $^{31}P\{^{1}H\}$ NMR spectrum (C_6D_6, 162 MHz) of **7**•$(Et_2O)_2$ at room temperature. An asterisk indicates a signal arising from impurity.

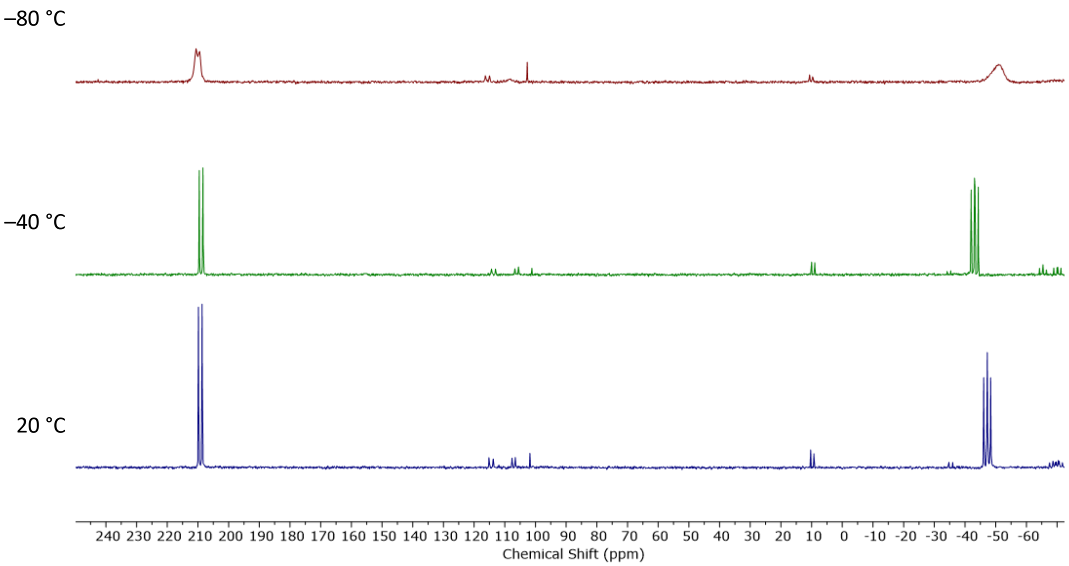

Figure S25. Variable temperature ^{31}P NMR spectra (toluene, 162 MHz) of **7**•$(Et_2O)_2$ at –80 °C, –40 °C, and 20 °C.

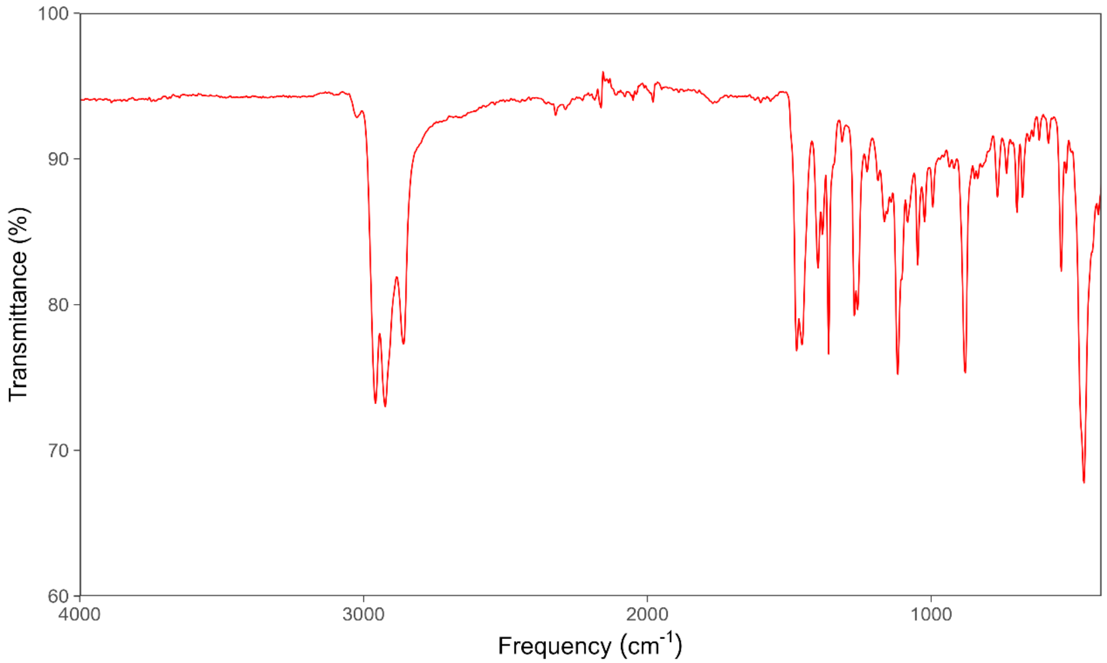

Figure S26. Experimental IR spectrum of **7**•$(Et_2O)_2$.

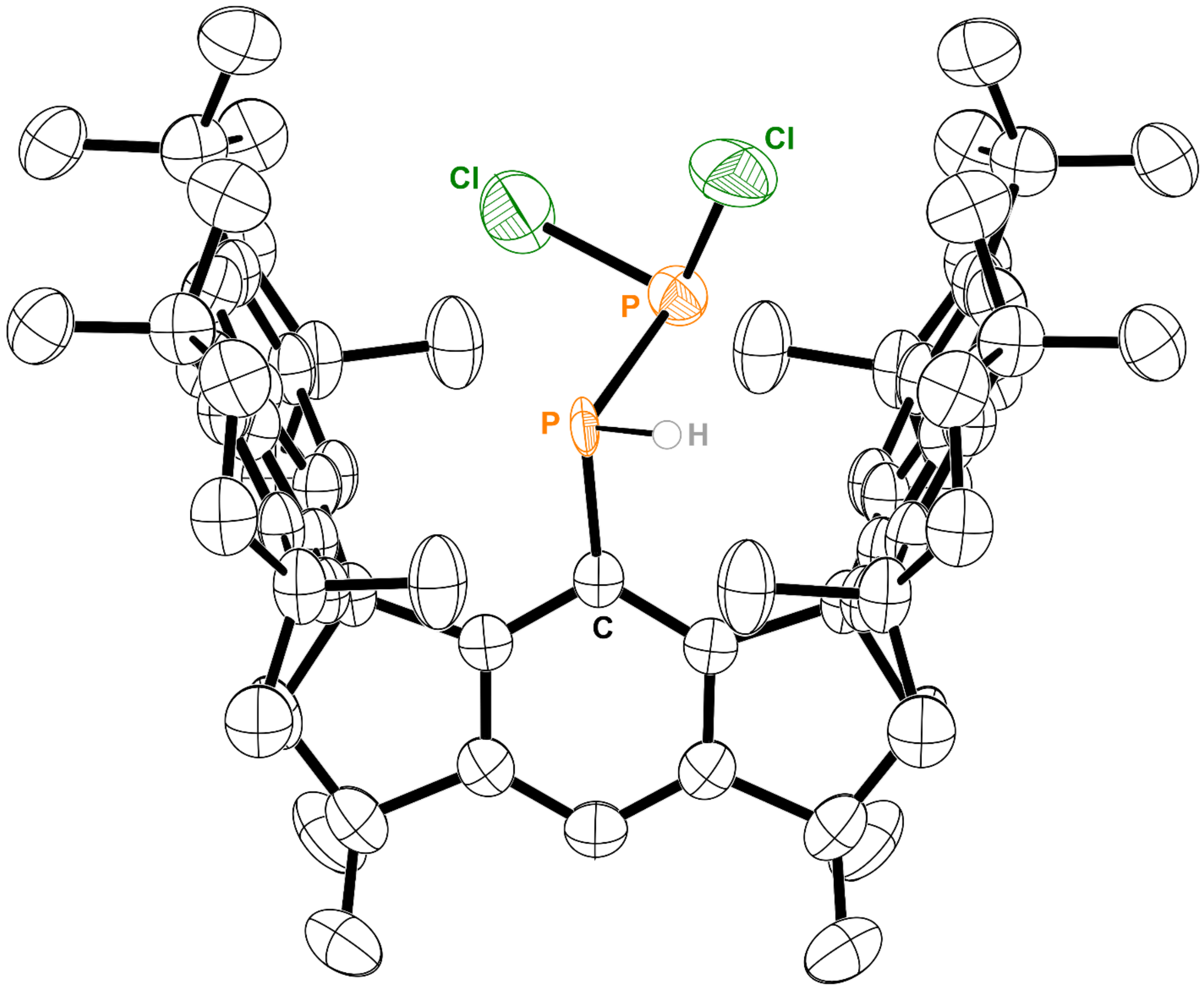

Figure S27. Thermal ellipsoid plot (50% probability) of **7**•$(Et_2O)_2$. Solvent molecules, C-bound H atoms, and disordered components are omitted for clarity. Color code: P orange, C black, Cl dark green, H grey.

2.5 Synthesis of ($M^sFluInd^*$)PPCl•(Et_2O)$_2$ (8•(Et_2O)$_2$)

Method A. A solution of **7**•(Et_2O)$_2$ (202 mg, 0.186 mmol) in hexane (6 mL) was treated with triethylamine (400 mg, 3.9 mmol). The mixture quickly became yellow and precipitated a colorless solid. After standing at room temperature for 4 h, the mixture was filtered into a new vial and placed in the freezer, using additional aliquots of hexane (3 × 1.5 mL) for quantitative transfer and extraction of the ammonium chloride filter cake. The yellow filtrate was cooled to –30 °C overnight. The following morning, the mixture was filtered again before being stripped of solvent. The solid residue was extracted with Et_2O, filtered through glass filter paper. The resulting yellow filtrate was concentrated and cooled to –30 °C to produce a batch of yellow crystalline solid. Yield: 138 mg (71%). Crystals suitable of **7**•(Et_2O)$_2$ for X-ray diffraction were grown from Et_2O at –30 °C.

Method B. Compound **6**•(Et_2O)$_2$ (50 mg, 0.041 mmol) and KBz (10 mg, 7.6 mmol) were suspended in benzene (1.6 mL) and stirred for 2 h. The red suspension was filtered into an ampoule and the volatiles were removed under vacuum. The resulting red residue was dissolved in Et_2O (4 mL) and cooled to –78 °C before being treated with a solution of PCl_3 (4.2 µL, 0.048 mmol) in Et_2O (410 uL). The reaction mixture rapidly became yellow and was allowed to warm to room temperature before being stripped of solvent. The ampoule was brought into a glovebox before being extracted with pentane, filtered and solvent removed under reduced pressure to yield a yellow powder, which was recrystallized from Et_2O at –30 °C. Yield: 24 mg (49%).

Note: Compound **8** was prepared in the absence of Et_2O for experiments involving $AlCl_3$ or $GaCl_3$ (*vide infra*), employing the following protocol: A solution of **7**•(Et_2O)$_2$ (249 mg, 0.201 mmol) in hexane (6 mL) was treated with triethylamine (400 mg, 3.9 mmol). The mixture quickly became yellow and precipitated a colorless solid. After standing at room temperature overnight, the mixture was filtered into a new vial and placed in the freezer for 3 h, using additional aliquots of hexane (3 × 1.5 mL) for quantitative transfer and extraction of the ammonium chloride filter cake. The mixture was filtered again before being stripped of solvent. The solid residue was dissolved in benzene (4 mL) before being filtered and stripped of solvent to afford a yellow powder of **8**. Spectral characteristics for **8** were identical to those of **8**•(Et_2O)$_2$, albeit in the absence of Et_2O. Yield: 184 mg (87%).

Elemental analysis, Found: C, 79.87; H, 9.12%. **Calc.** for $C_{80}H_{109}P_2ClO_2$: C, 80.06; H, 9.15%.

ESI-MS (m/z) [8+Na]$^+$ 1073.698 (calc 1073.602).

^{1}H NMR (400 MHz, C_6D_6): δ = 7.74 (s, 4H), 7.32 (s, 1H), 7.30 (s, 1H), 2.58 (s, 4 H), 1.70-1.50 (m, 28 H), 1.35-1.17 (m, 48 H) ppm.

^{13}C{^{1}H} NMR (101 MHz, C_6D_6): δ = 154.6, 144.7, 144.4, 138.3, 123.1, 118.2, 118.0, 63.1, 57.1, 43.1, 35.7, 35.6, 34.9, 34.7, 33.0, 32.7, 32.6, 32.4, 32.3, 32.2, 32.0 ppm.

^{31}P NMR (162 MHz, C_6D_6): δ = 501.5 (d, $^1J_{PP}$ = 574 Hz), 433.2 (d, $^1J_{PP}$ = 574 Hz) ppm.

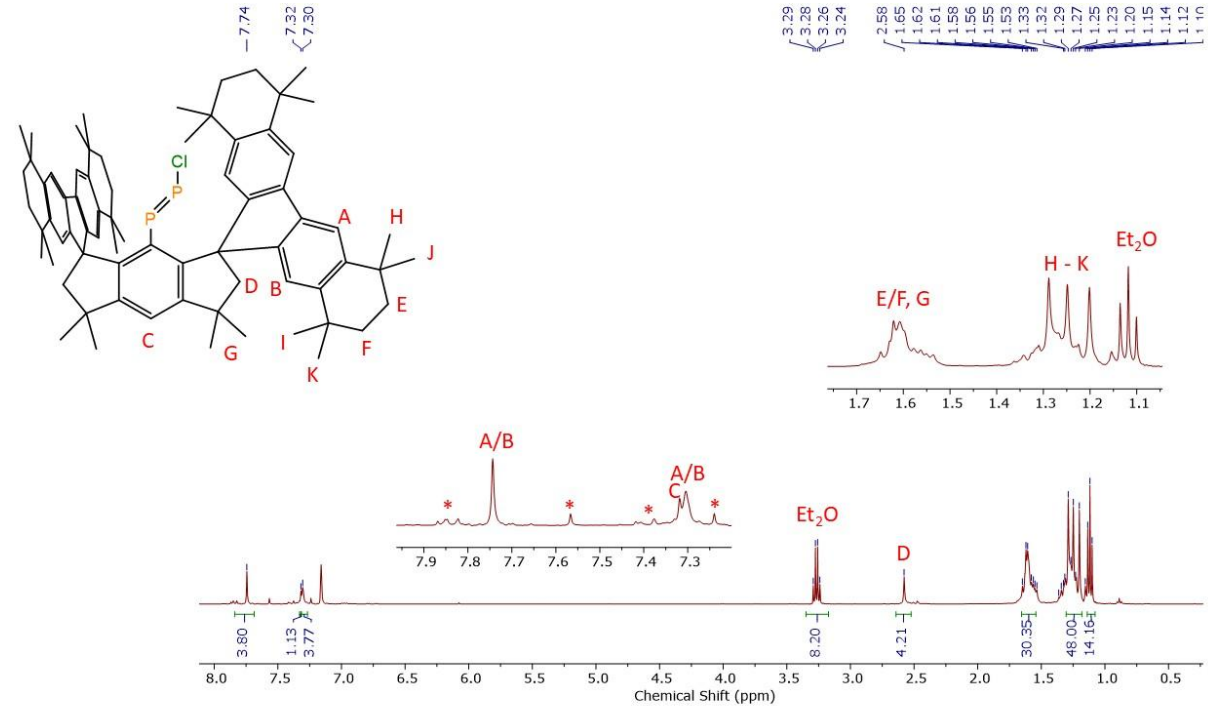

Figure S28. ^{1}H NMR spectrum (C_6D_6, 400 MHz) of **8**•$(Et_2O)_2$ at room temperature. An asterisk indicates a signal arising from impurity.

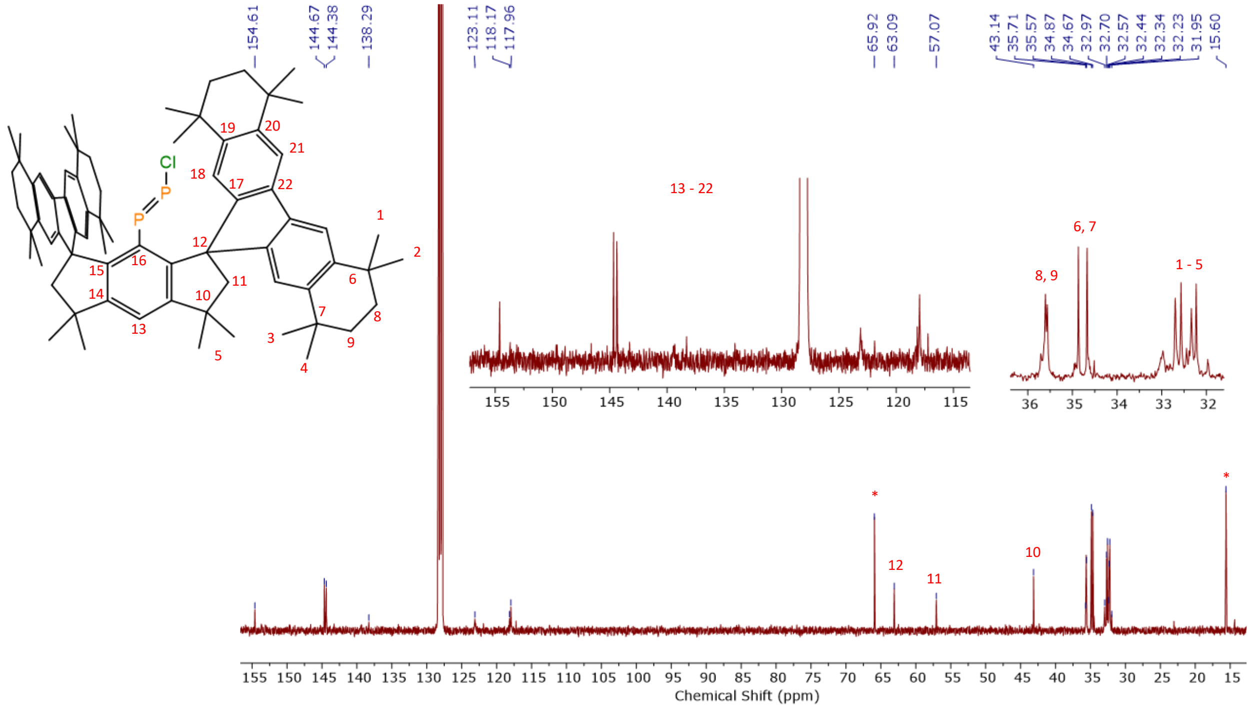

Figure S29. $^{13}C\{^{1}H\}$ NMR spectrum (C_6D_6, 101 MHz) of **8**•$(Et_2O)_2$ at room temperature. The asterisks denotes a signal from the Et_2O solvate.

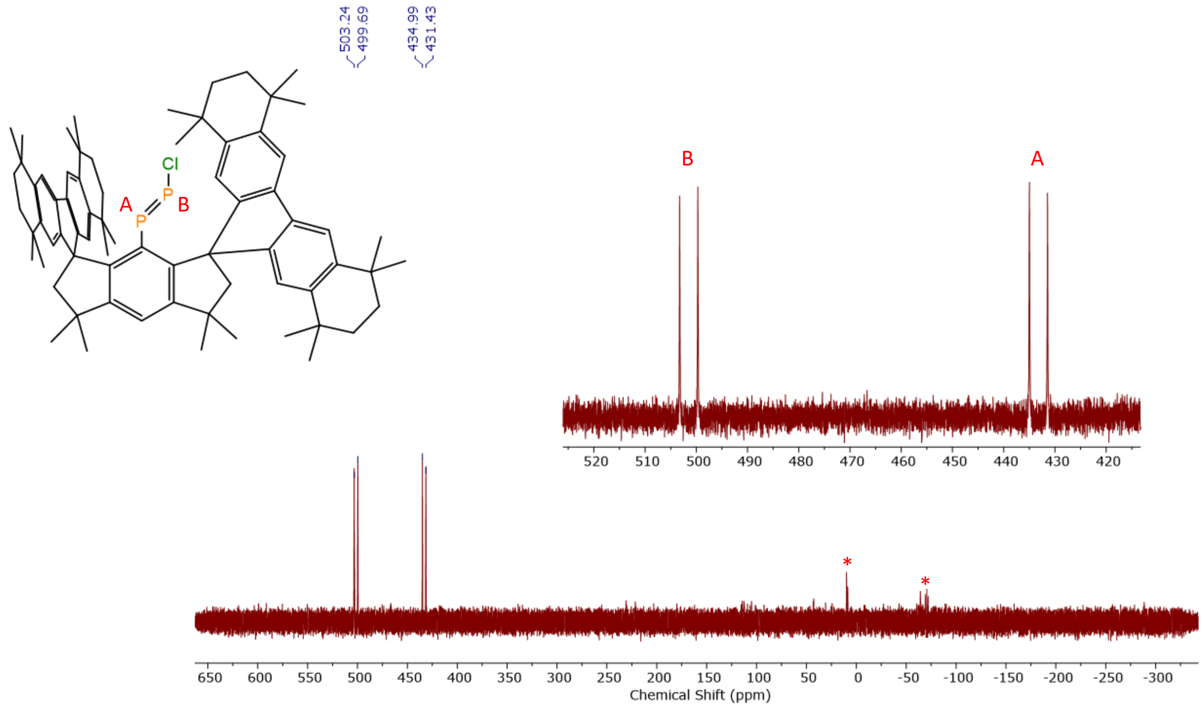

Figure S30. ^{31}P NMR spectrum (C_6D_6, 162 MHz) of **8**•$(Et_2O)_2$ at room temperature. An asterisk indicates a signal arising from impurity.

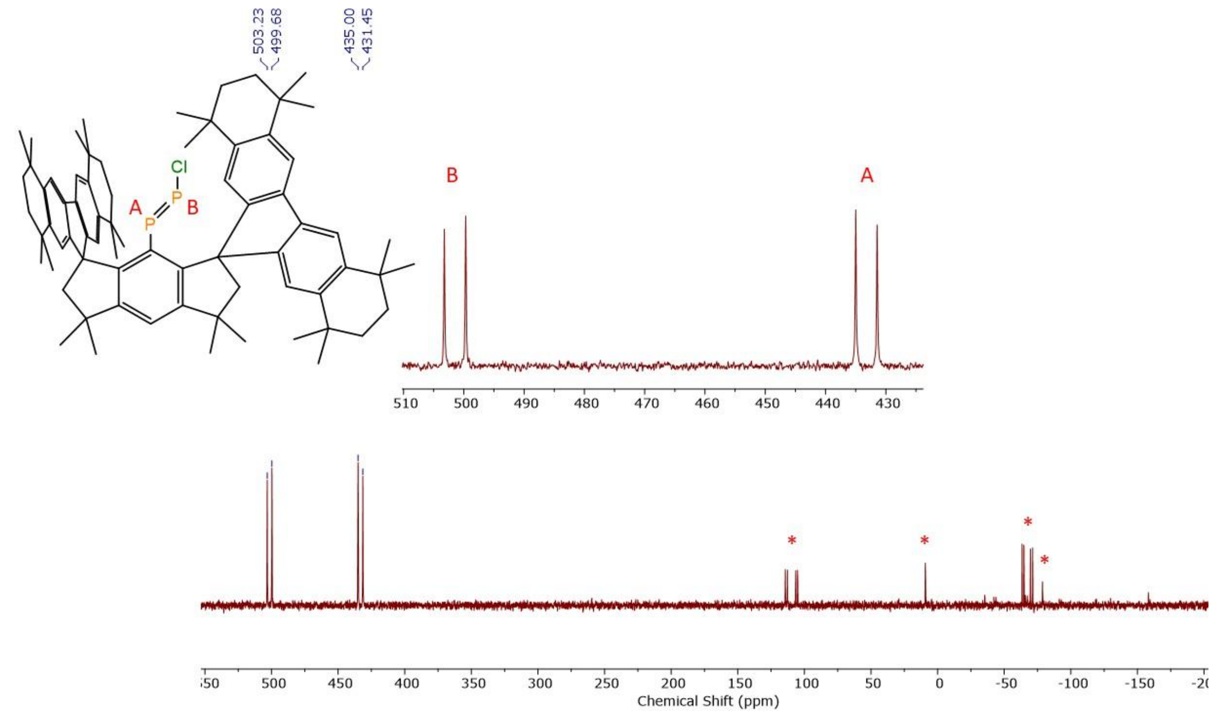

Figure S31. $^{31}P\{^{1}H\}$ NMR spectrum (C_6D_6, 162 MHz) of **8**•$(Et_2O)_2$ at room temperature. An asterisk indicates a signal arising from impurity.

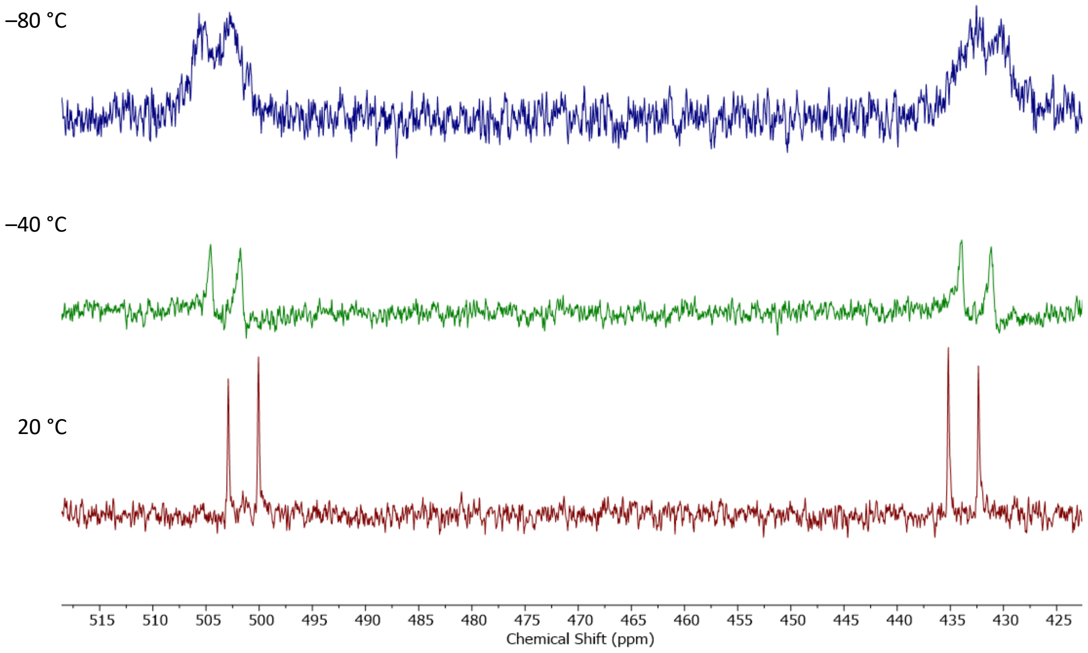

Figure S32. Variable temperature ^{31}P NMR spectra (toluene, 202 MHz) of **8**•$(Et_2O)_2$ at –80 °C, –40 °C, and 20 °C.

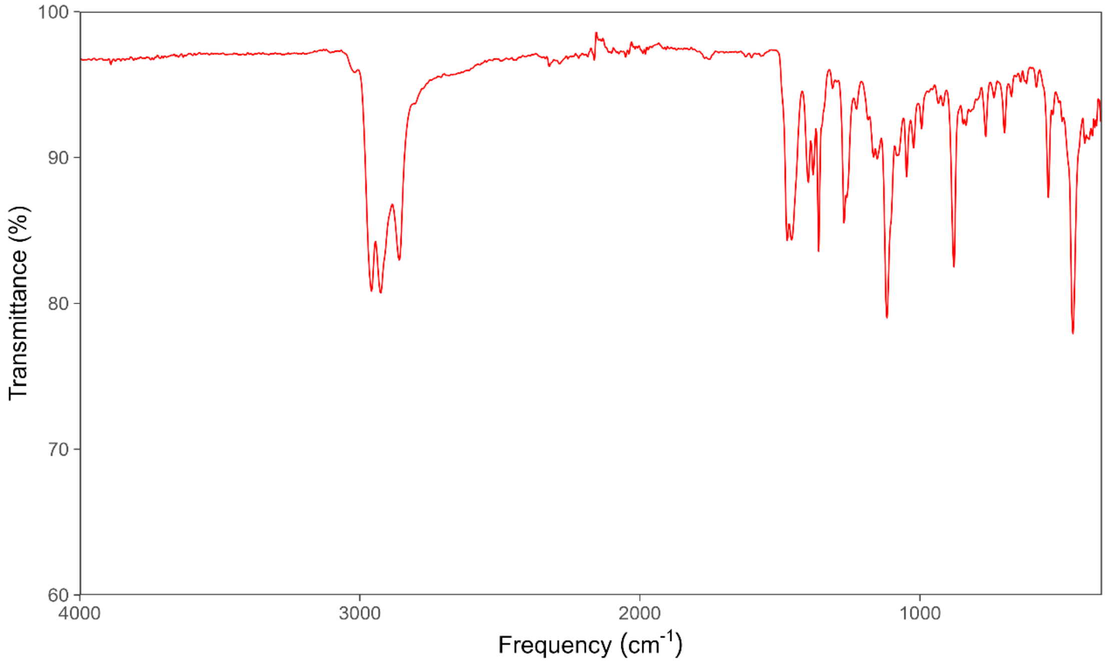

Figure S33. Experimental IR spectrum of **8**•(Et_2O)$_2$. While the IR absorbance associated with the P–P stretch cannot be clearly identified due to its low intensity and position within the fingerprint region,[41] the strong band assigned to the P–Cl stretch appears at a lower wavenumber ($\nu_{P–Cl}$ = 451 cm^{-1}) relative to that of **7**•(Et_2O)$_2$ ($\nu_{P–Cl}$ = 461 cm^{-1}) (Supplementary Figure S34).

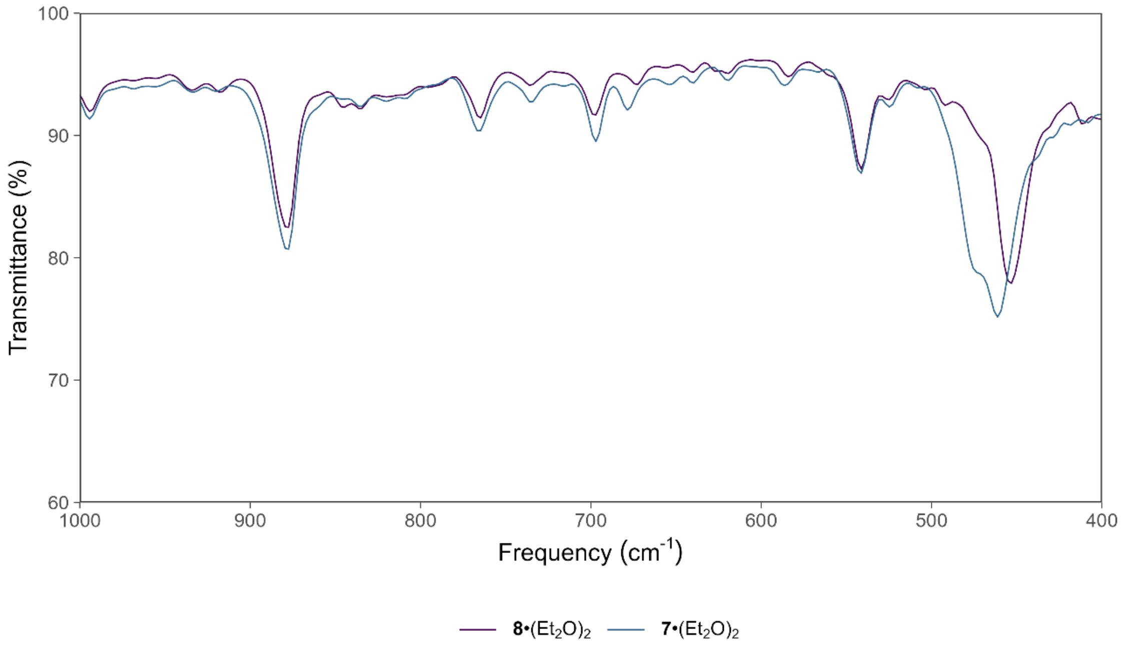

Figure S34. Experimental IR spectra of **7**•$(Et_2O)_2$ and **8**•$(Et_2O)_2$.

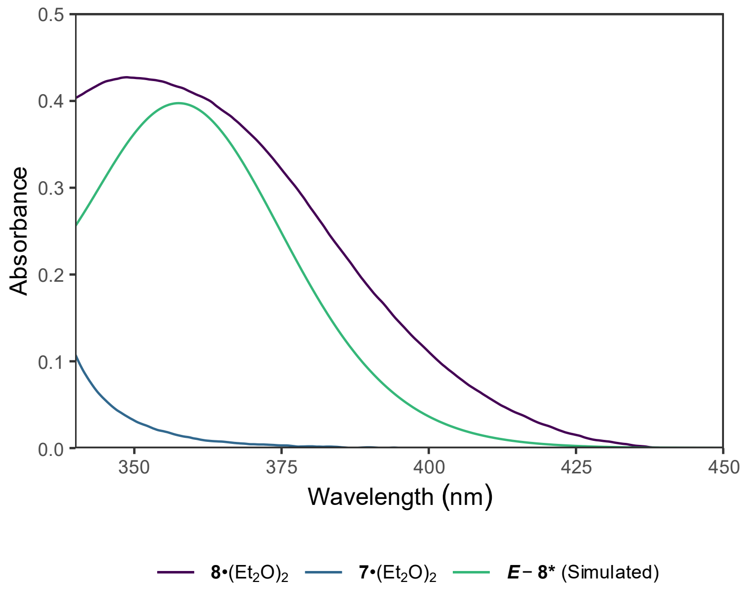

Figure S35. Experimental UV-Vis spectra of **7**•$(Et_2O)_2$ (86 μM) and **8**•$(Et_2O)_2$ (104 μM) in benzene at room temperature. Simulated UV-Vis spectrum (DKH-PBE0/old-DKH-TZVPP) of ***E*-8*** (further information provided in Supplementary Table S12).

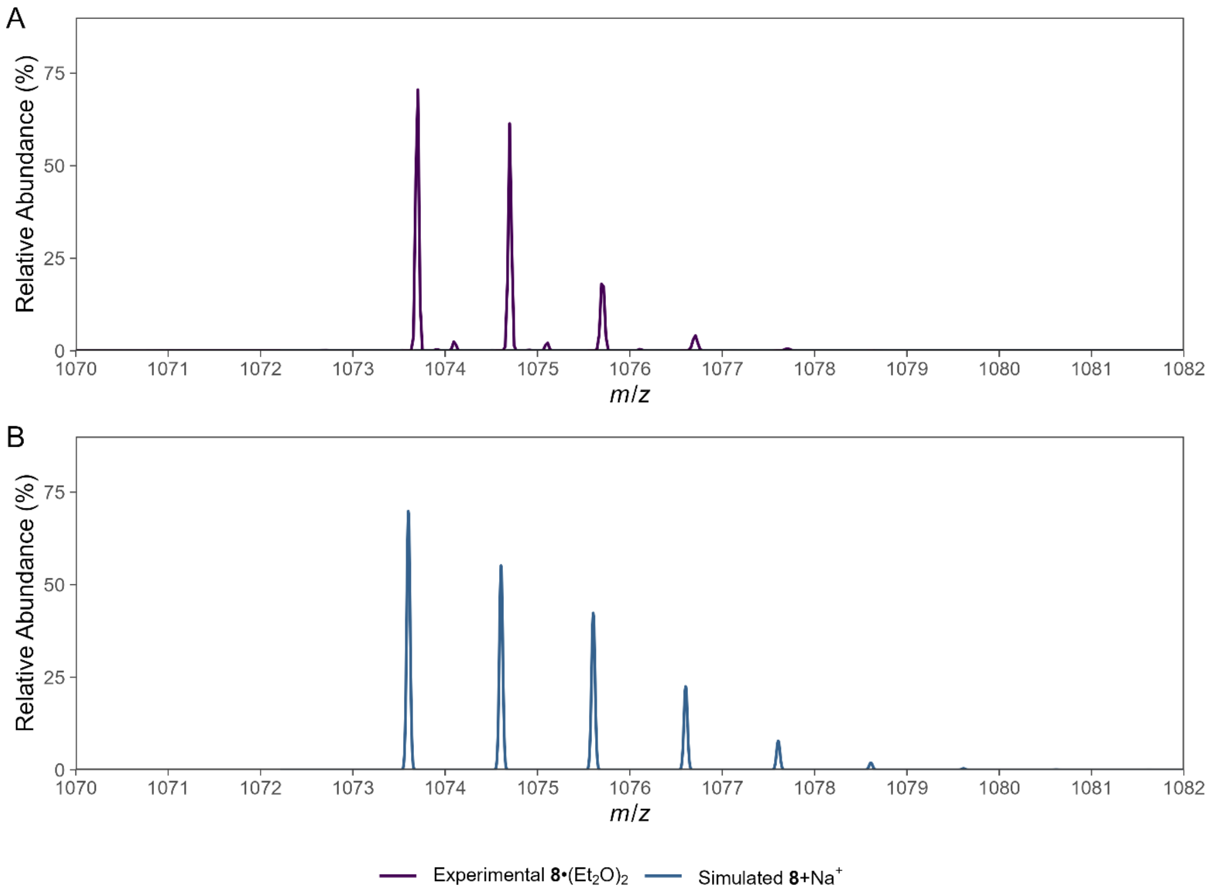

Figure S36. (A) Experimental ESI-MS spectrum for **8**•$(Et_2O)_2$. (B) Simulated ESI-MS spectrum for **8**+Na^+.

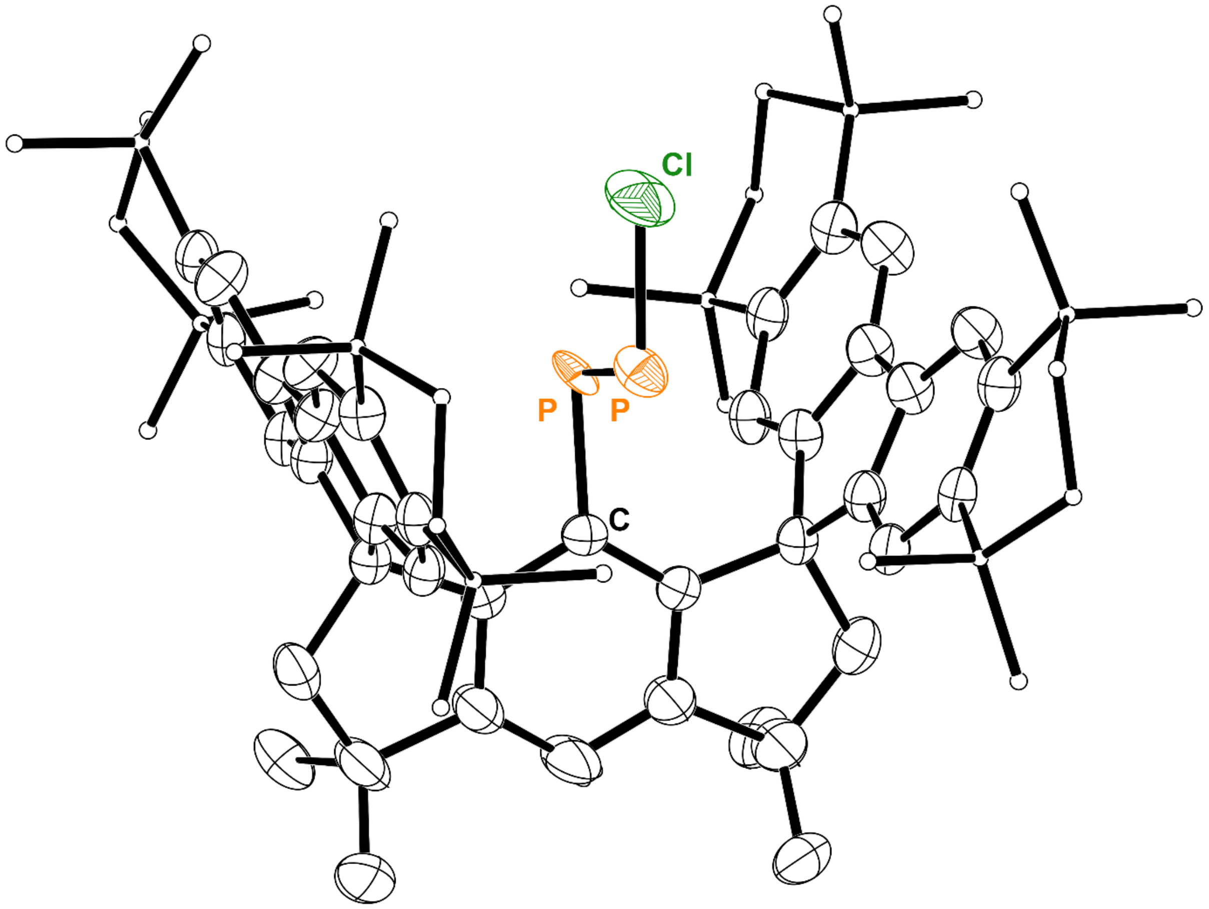

Figure S37. Thermal ellipsoid plot (50% probability) of **8**•(Et_2O)$_2$ (image showing major *E* component of disorder). Solvent molecules, H atoms, and disordered components are omitted for clarity. Select C atoms are shown as spheres of arbitrary radius for clarity. Color code: P orange, Cl dark green, C black, H grey.

.

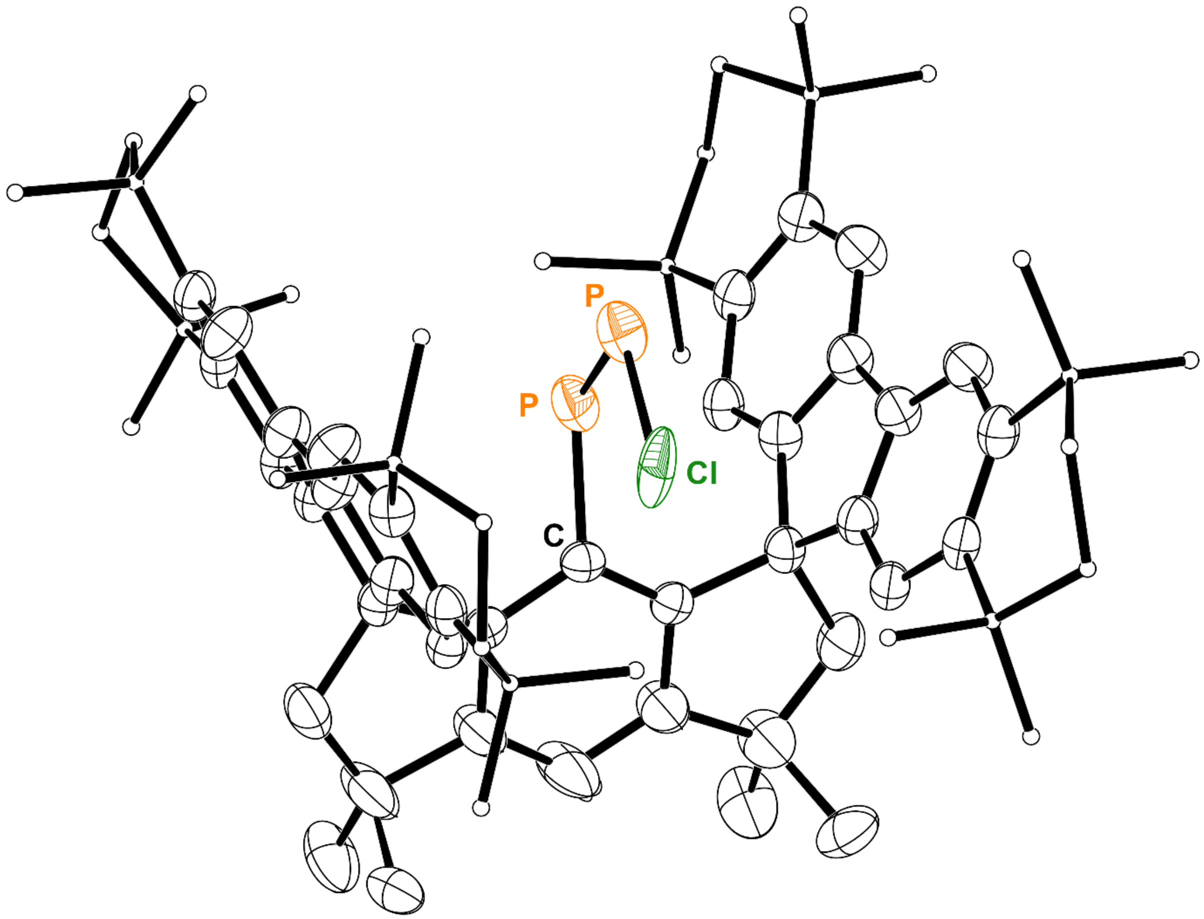

Figure S38. Thermal ellipsoid plot (50% probability) of **8**•$(Et_2O)_2$ (image showing minor *Z* component of disorder). Solvent molecules, H atoms, and disordered components are omitted for clarity. Select C atoms are shown as spheres of arbitrary radius for clarity. Color code: P orange, Cl dark green, C black.

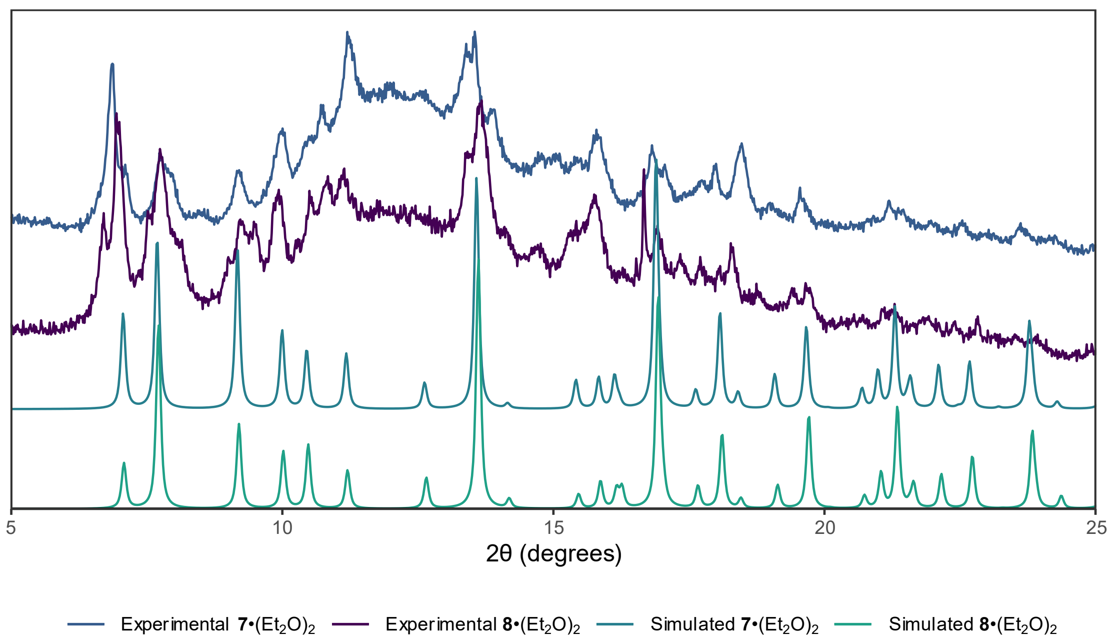

Figure S39. Simulated and experimental powder X-ray diffractograms for **7**•$(Et_2O)_2$ and **8**•$(Et_2O)_2$.

2.6 Synthesis of (M^{s}FluInd*)PPBr•(Et_2O)$_2$ (9•(Et_2O)$_2$)

A solution of **8**•(Et_2O)$_2$ (63 mg, 0.053 mmol) in toluene (0.6 mL) was treated with TMSBr (288 mg, 1.88 mmol) and heated to 100 °C for 16 h. The solvent was stripped, and the resulting residue was recrystallized from Et_2O at –30 °C to afford a batch of yellow crystals that were dried under vacuum. Yield: 41 mg (63 %). Crystals suitable for X-ray diffraction were grown from Et_2O at –30 °C.

Elemental analysis, Found: C, 77.81; H, 8.84%. **Calc.** for $C_{80}H_{109}P_2BrO_2$: C, 77.20; H, 8.83%.

ESI-MS (m/z) [4+H]$^{+}$ 987.686 (calc. 987.693); only the protonolysis product **4** was able to be assigned in the ESI-MS spectrum.

^{1}H NMR (400 MHz, C_6D_6): δ = 7.75 (s, 4H), 7.31 (s, 1H), 7.30 (s, 1H), 2.58 (s, 4 H), 1.66-1.54 (m, 28 H), 1.36-1.20 (m, 48 H) ppm.

^{13}C{^{1}H} NMR (101 MHz, C_6D_6): δ = 154.3, 144.3, 144.0, 122.7, 117.8, 62.6, 56.6, 42.8, 35.2, 34.5, 34.3, 32.6, 32.3, 32.2, 32.0, 31.9 ppm.

^{31}P NMR (162 MHz, C_6D_6): δ = 489.6 (d, $^{1}J_{PP}$ = 567 Hz), 444.8 (d, $^{1}J_{PP}$ = 567 Hz) ppm.

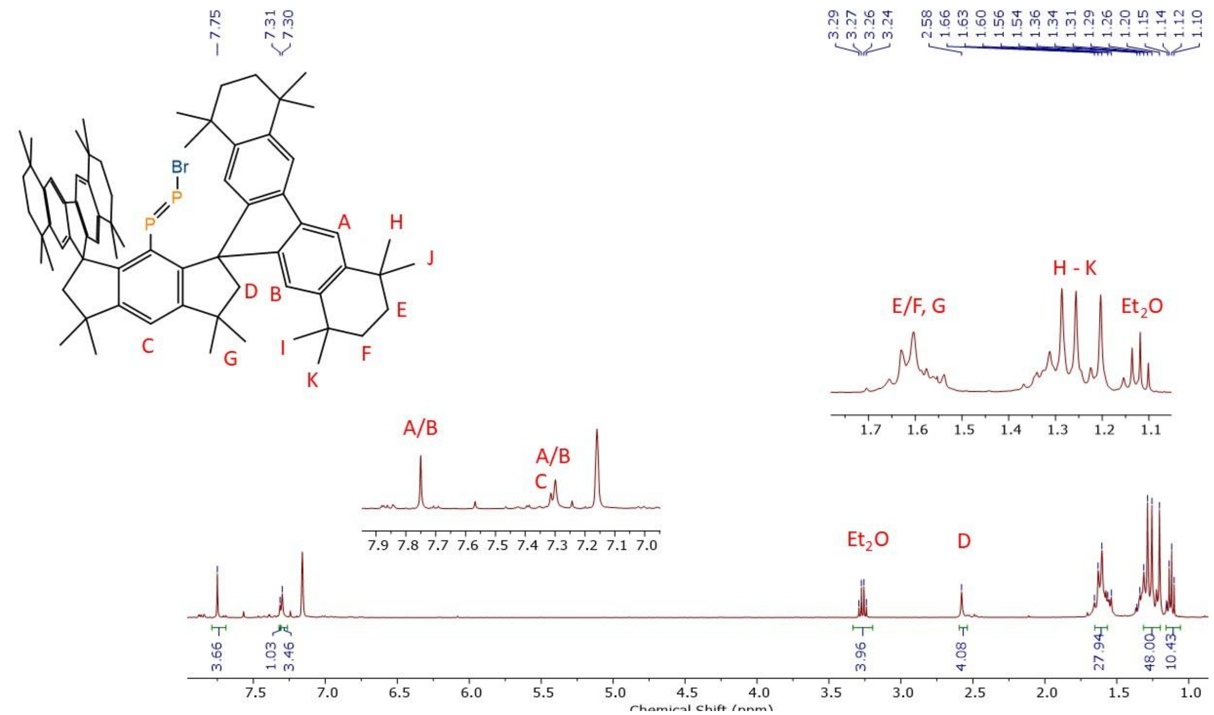

Figure S40. ^{1}H NMR spectrum (C_6D_6, 400 MHz) of **9**•$(Et_2O)_2$ at room temperature.

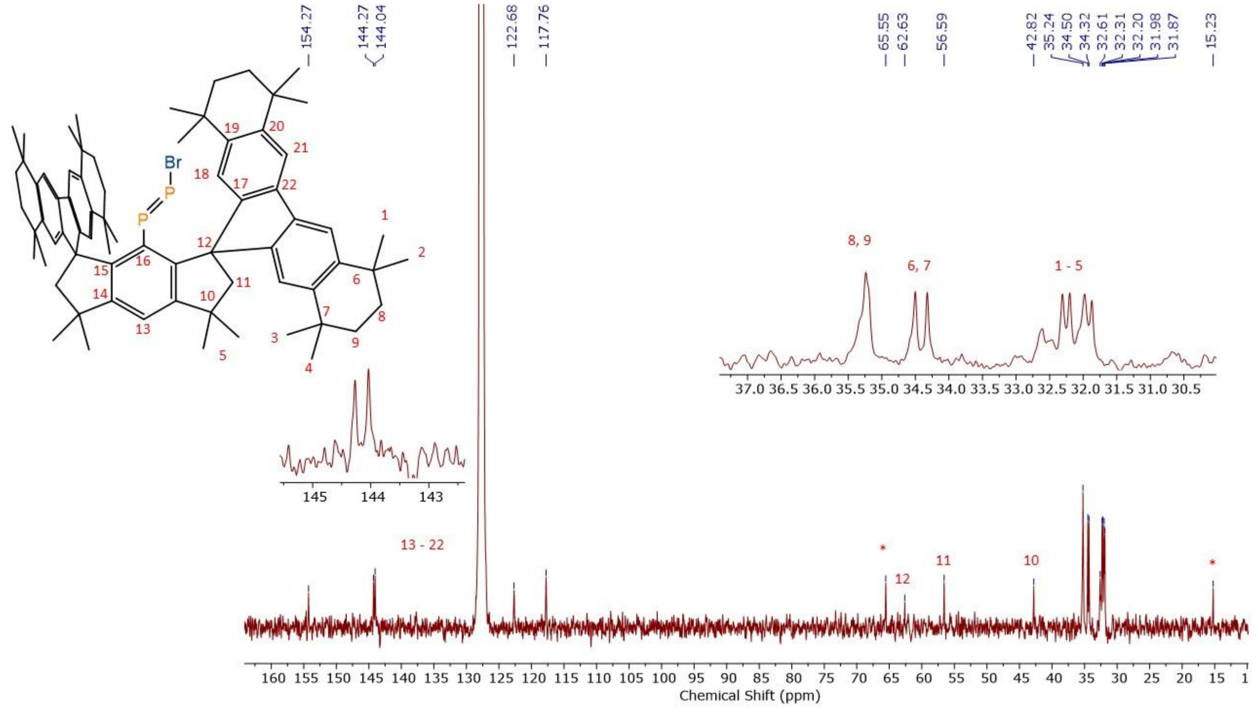

Figure S41. $^{13}C\{^{1}H\}$ NMR spectrum (C_6D_6, 101 MHz) of **9**•$(Et_2O)_2$ at room temperature. The asterisks denotes a signal from the Et_2O solvate.

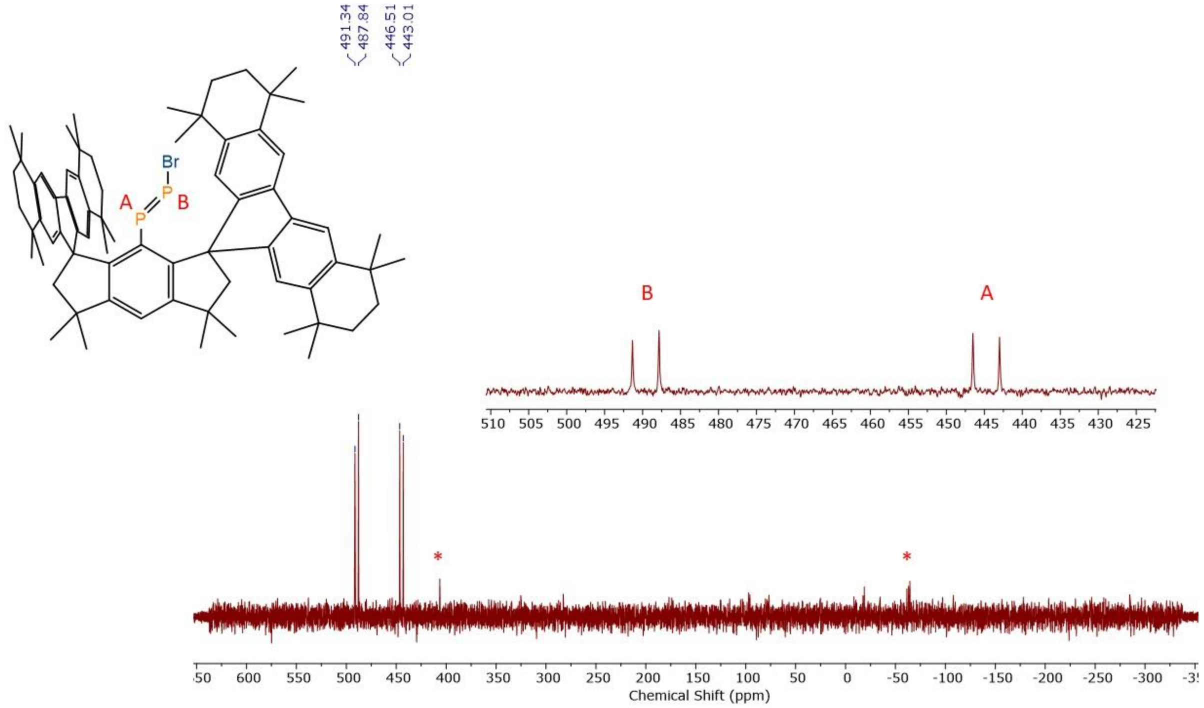

Figure S42. ^{31}P NMR spectrum (C_6D_6, 162 MHz) of **9**•$(Et_2O)_2$ at room temperature. An asterisk denotes signals arising from impurity.

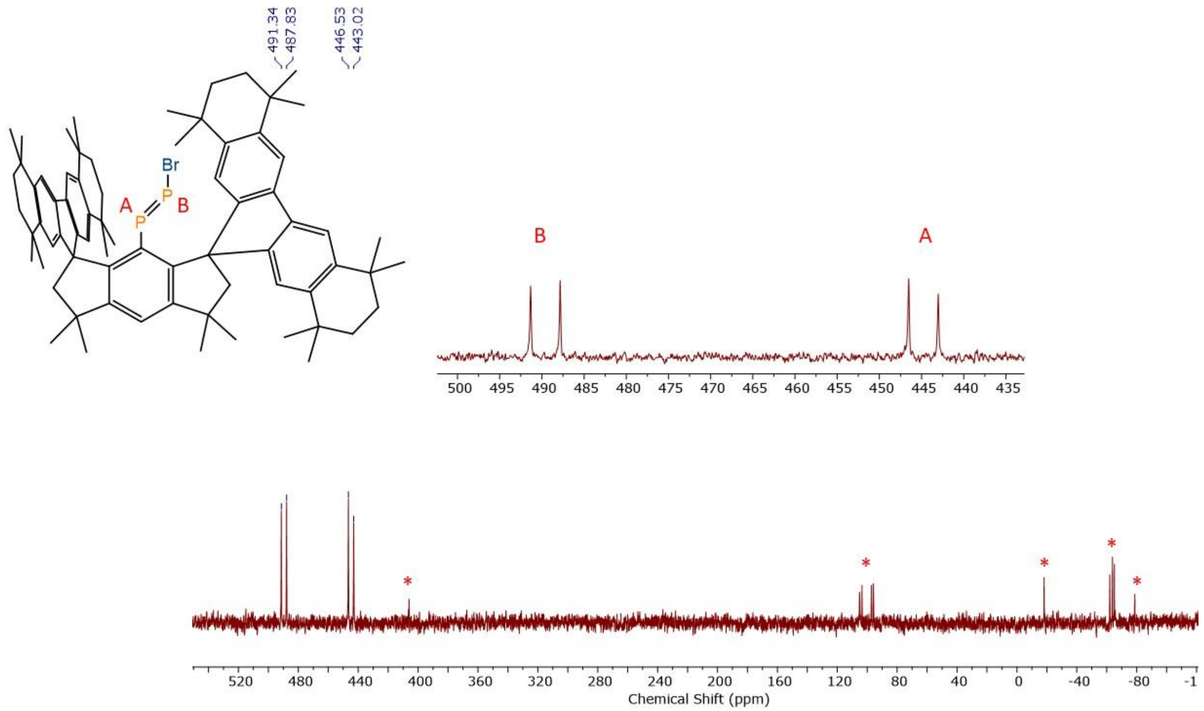

Figure S43. $^{31}P\{^1H\}$ NMR spectrum (C_6D_6, 162 MHz) of **9**•$(Et_2O)_2$ at room temperature. An asterisk denotes signals arising from impurity.

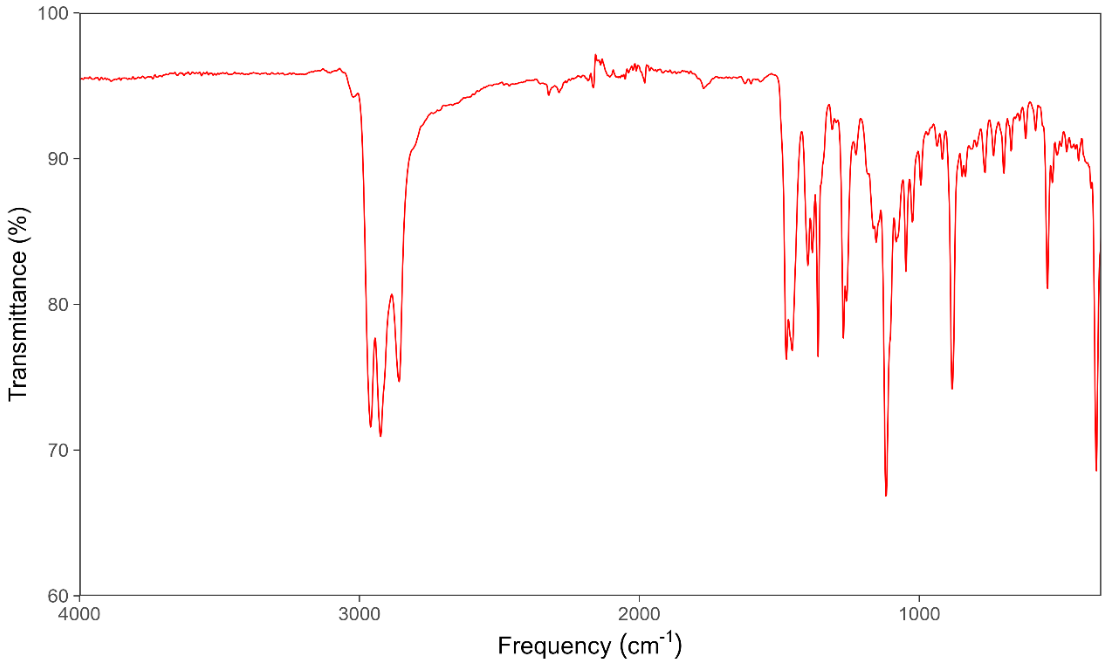

Figure S44. Experimental IR spectrum of **9**•$(Et_2O)_2$. The strong band assigned to the P–Br stretch appears at $\nu_{P–Br}$ = 367 cm^{-1}.

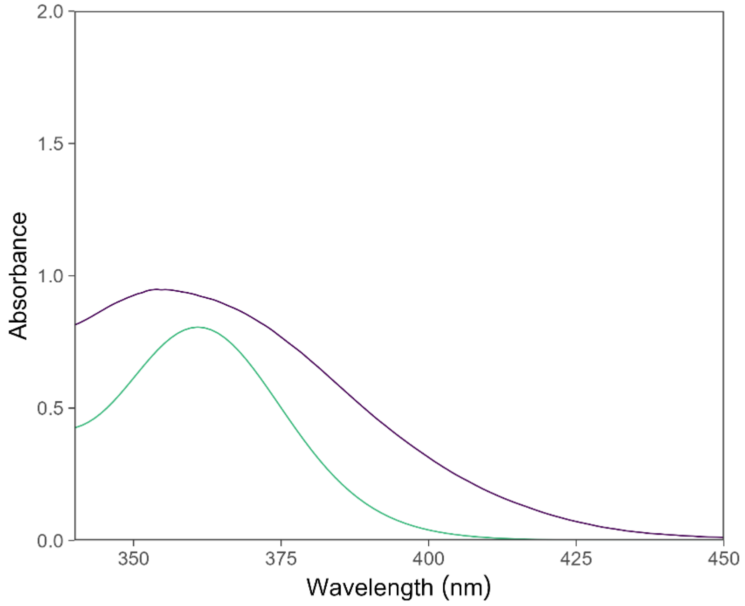

Figure S45. Experimental UV-Vis spectrum of **9**•$(Et_2O)_2$ (110 μM) in benzene at room temperature. Simulated UV-Vis spectrum (DKH-PBE0/old-DKH-TZVPP) of ***E*-9*** (further information provided in Supplementary Table S13).

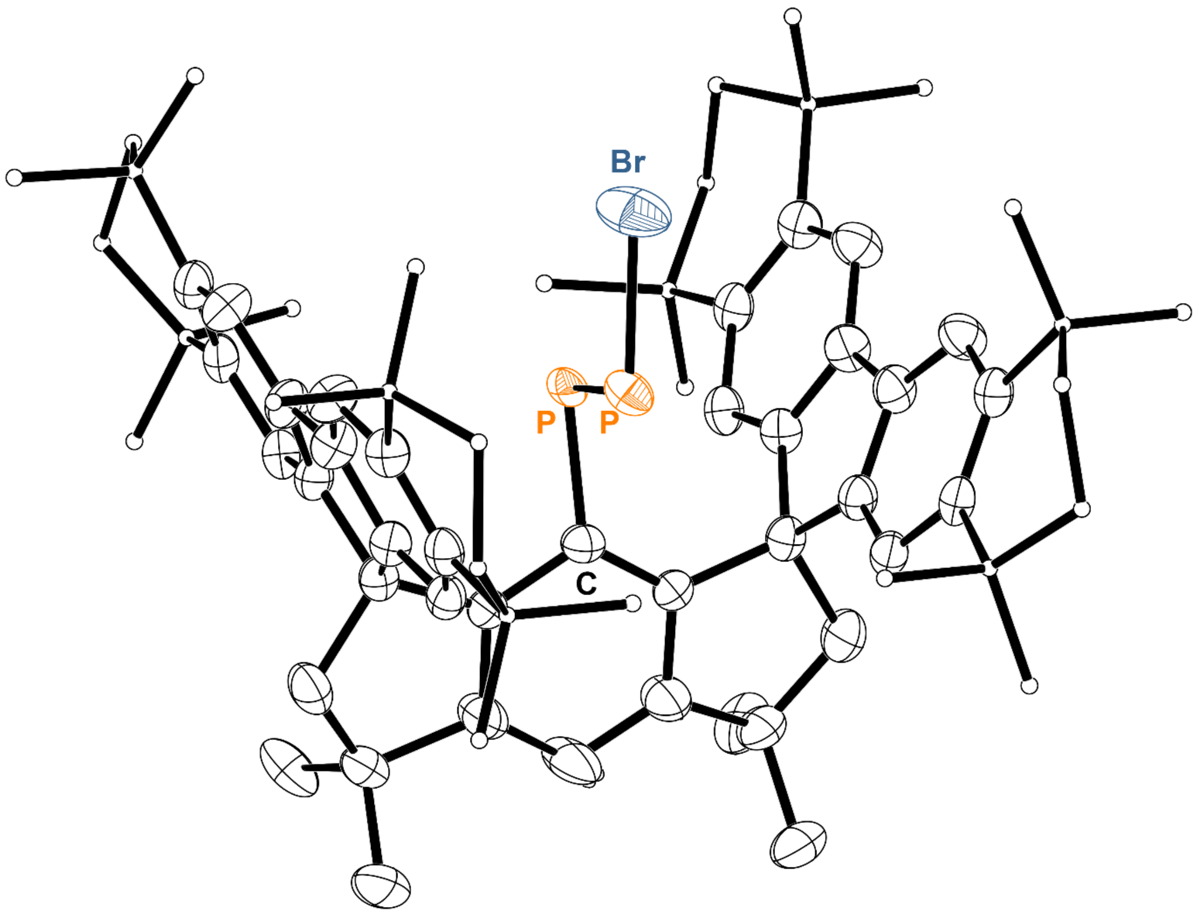

Figure S46. Thermal ellipsoid plot (50% probability) of **9**•$(Et_2O)_2$. (image showing major *E* component) Solvent molecules, C-bound H atoms, and disordered components are omitted for clarity. Select C atoms are shown as spheres of arbitrary radius for clarity. Color code: P orange, C black, H grey, Br blue.

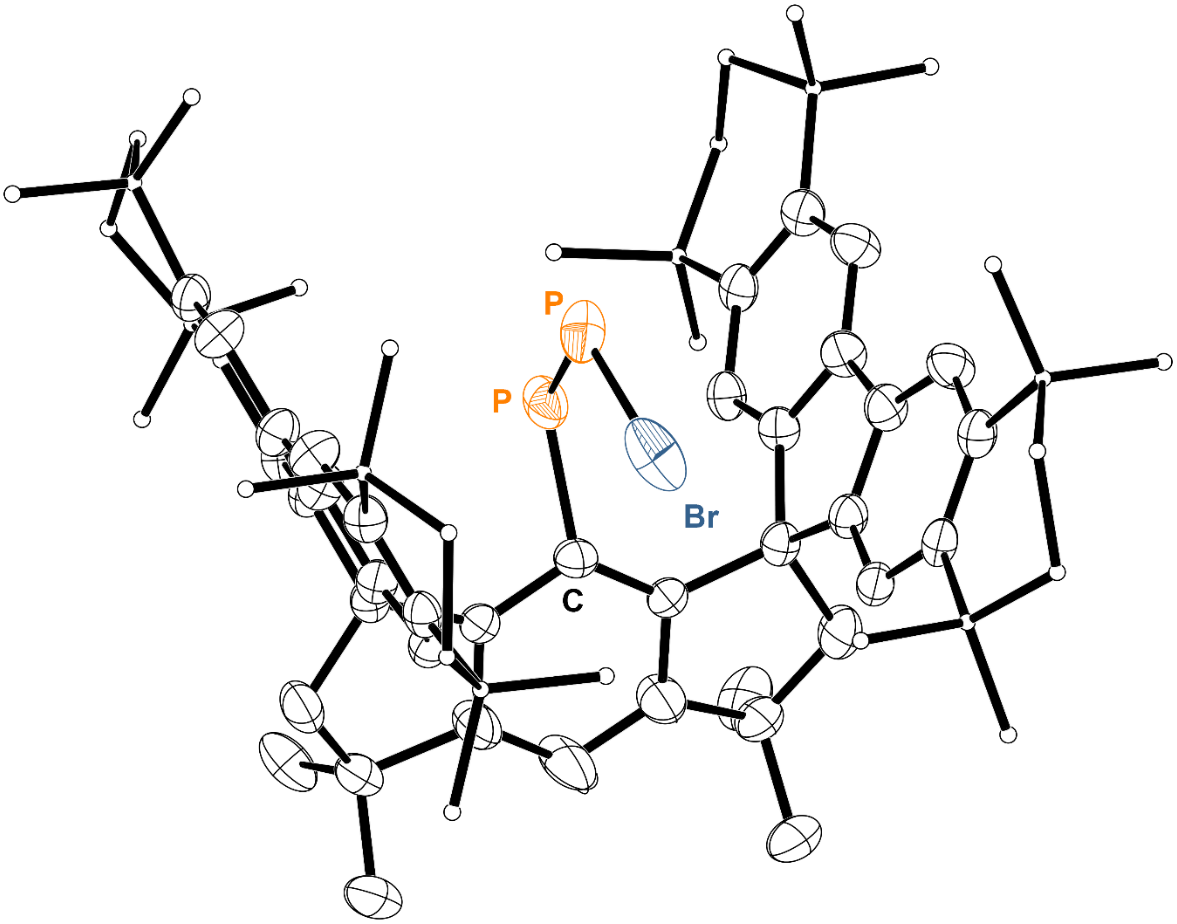

Figure S47. Thermal ellipsoid plot (50% probability) of **9**•$(Et_2O)_2$ (image showing minor *Z* component). Solvent molecules, C-bound H atoms, and disordered components are omitted for clarity. Select C atoms are shown as spheres of arbitrary radius for clarity. Color code: P orange, C black, Br blue.

2.7 Synthesis of (M^sFluInd*)PPI•(Et$_2$O)$_2$ (10•(Et$_2$O)$_2$)

A solution of **8**•(Et$_2$O)$_2$ (82 mg, 0.068 mmol) in toluene (0.6 mL) was treated with TMSI (327 mg, 1.63 mmol) and heated to 100 °C for 16 h. The solvent was stripped, and the resulting residue was recrystallized from Et$_2$O at –30 °C. The resulting yellow crystalline product was dried under vacuum. Yield: 53 mg (60%). Crystals suitable for X-ray diffraction were grown from Et$_2$O at –30 °C.

Elemental analysis, Found: C, 74.45; H, 8.70%. **Calc.** for $C_{80}H_{109}P_2IO_2$: C, 74.39; H, 8.51%.

ESI-MS (m/z) [10+Na]$^+$ 1165.759 (calc 1165.538).

^{1}H NMR (400 MHz, C_6D_6): δ = 7.75 (s, 4H), 7.32 (s, 1H), 7.29 (s, 1H), 2.57 (s, 4 H), 1.67-1.51 (m, 28 H), 1.38-1.19 (m, 48 H) ppm.

^{13}C{^{1}H} NMR (101 MHz, C_6D_6): δ = 154.3, 144.3, 144.0, 122.7, 117.8, 62.6, 56.6, 42.8, 35.2, 34.5, 34.3, 32.6, 32.3, 32.2, 32.0, 31.9 ppm.

^{31}P NMR (162 MHz, C_6D_6): δ = 474.4 (d, $^1J_{PP}$ = 554 Hz), 457.0 (d, $^1J_{PP}$ = 554 Hz) ppm.

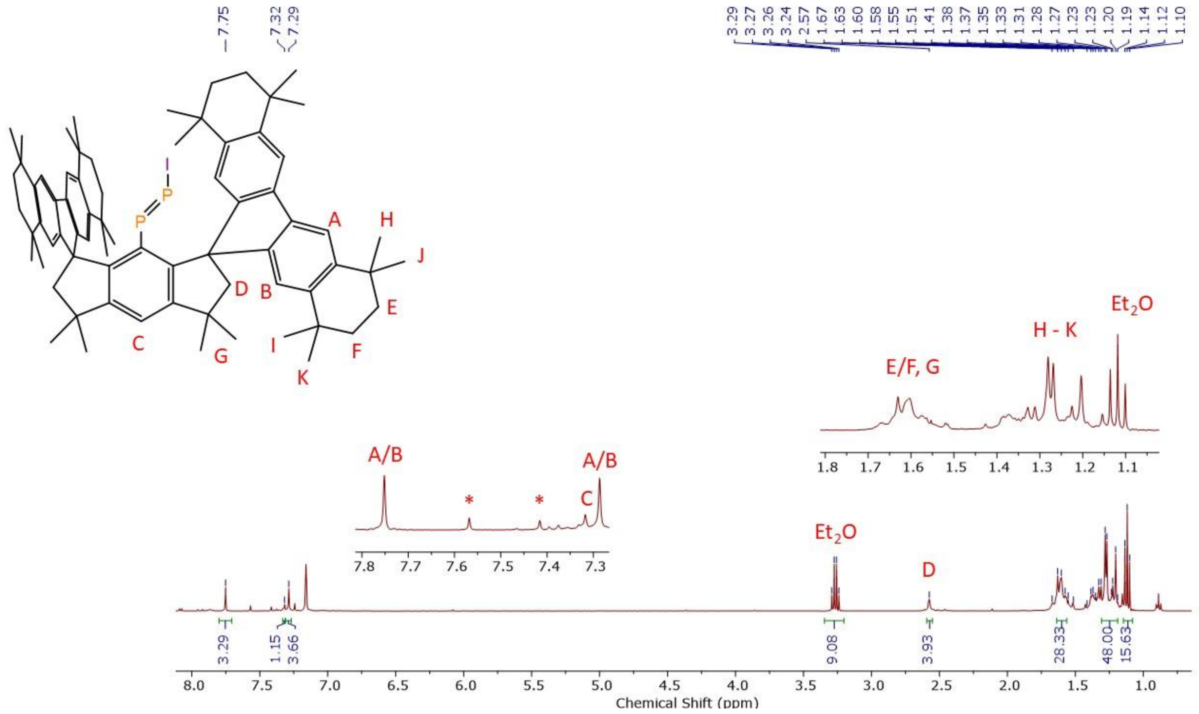

Figure S48. ^{1}H NMR spectrum (C_6D_6, 400 MHz) of **10**•(Et$_2$O)$_2$ at room temperature. An asterisk denotes a signal arising from impurity.

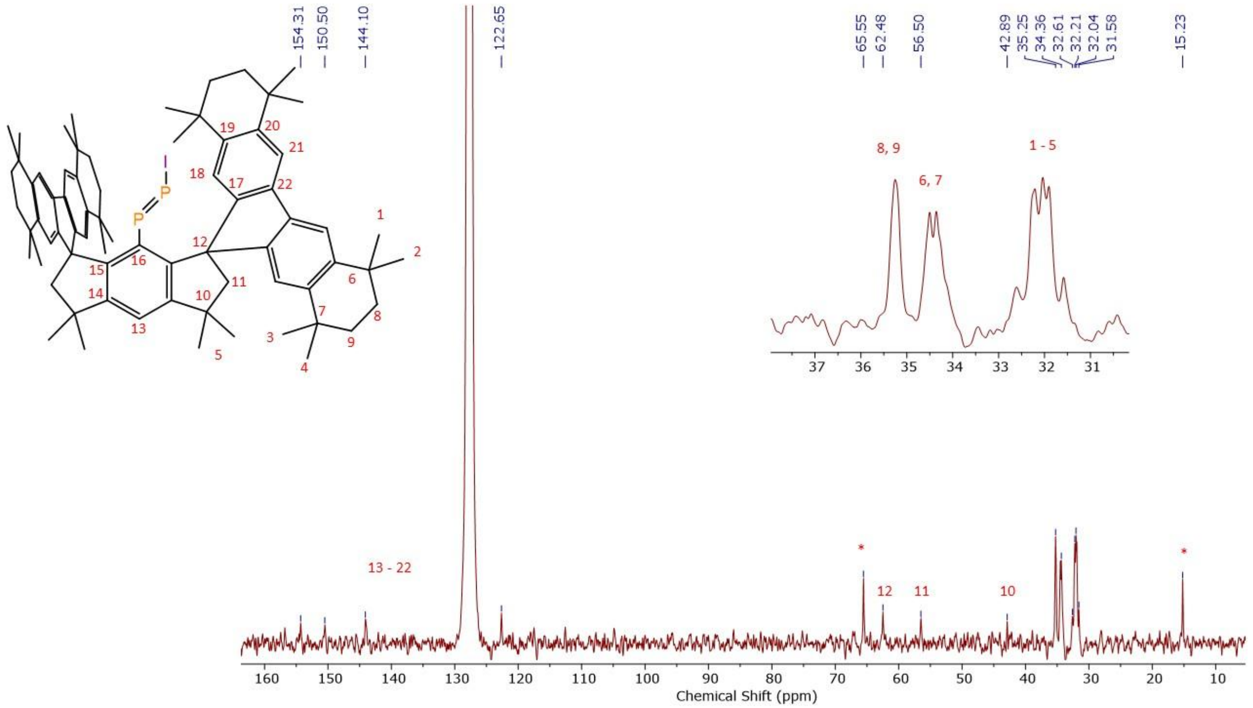

Figure S49. $^{13}C\{^{1}H\}$ NMR spectrum (C_6D_6, 101 MHz) of **10**•$(Et_2O)_2$ at room temperature. The asterisks denotes a signal from the Et_2O solvate.

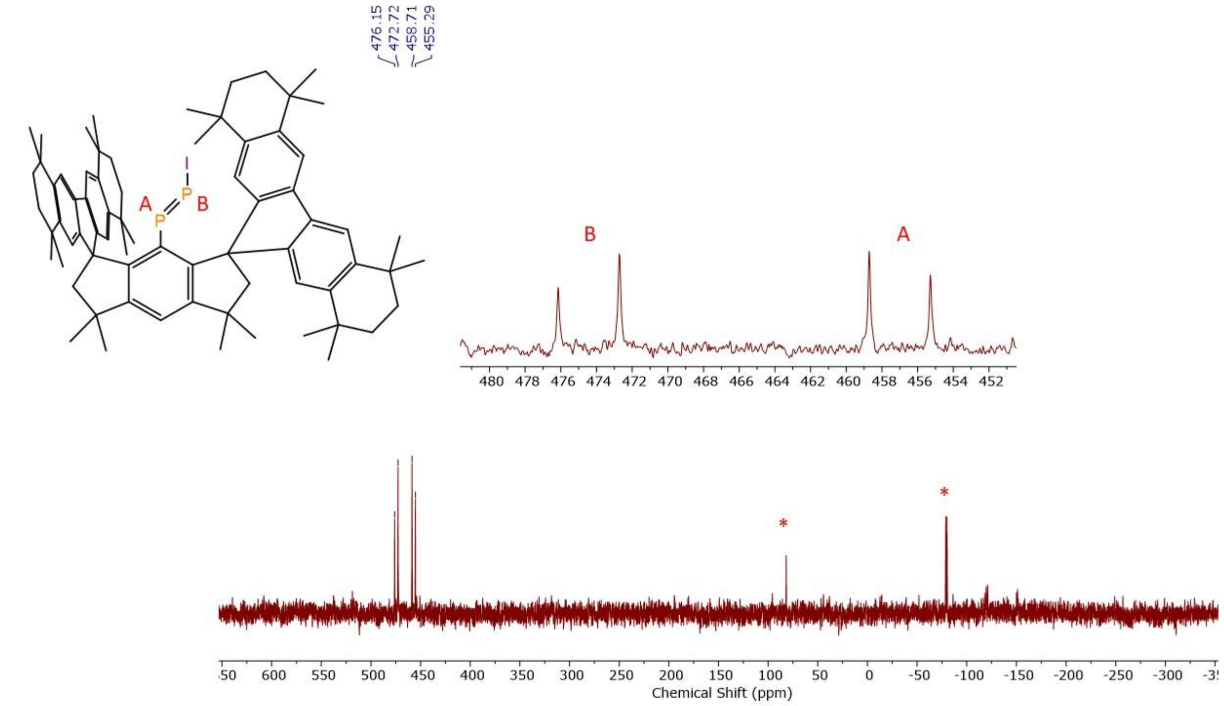

Figure S50. ^{31}P NMR spectrum (C_6D_6, 162 MHz) of **10**•$(Et_2O)_2$ at room temperature. The asterisks denote signals arising from impurity.

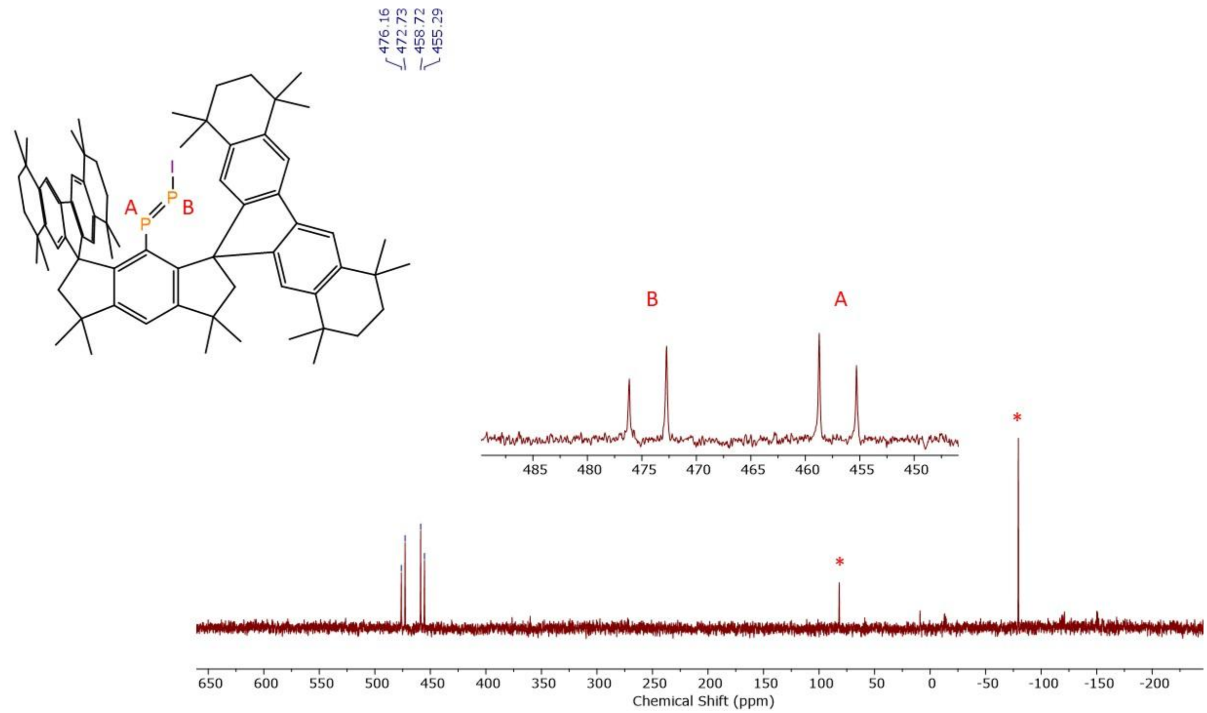

Figure S51. $^{31}P\{^{1}H\}$ NMR spectrum (C_6D_6, 162 MHz) of **10•**$(Et_2O)_2$ at room temperature. The asterisks denote signals arising from impurity.

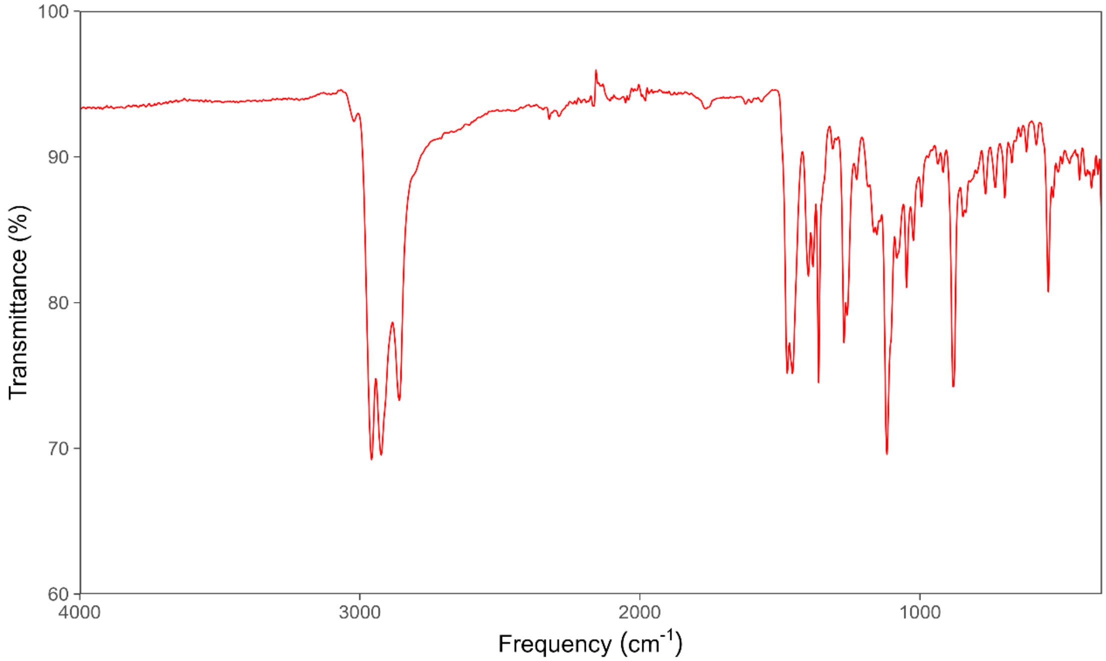

Figure S52. Experimental IR spectrum of **10•**$(Et_2O)_2$.

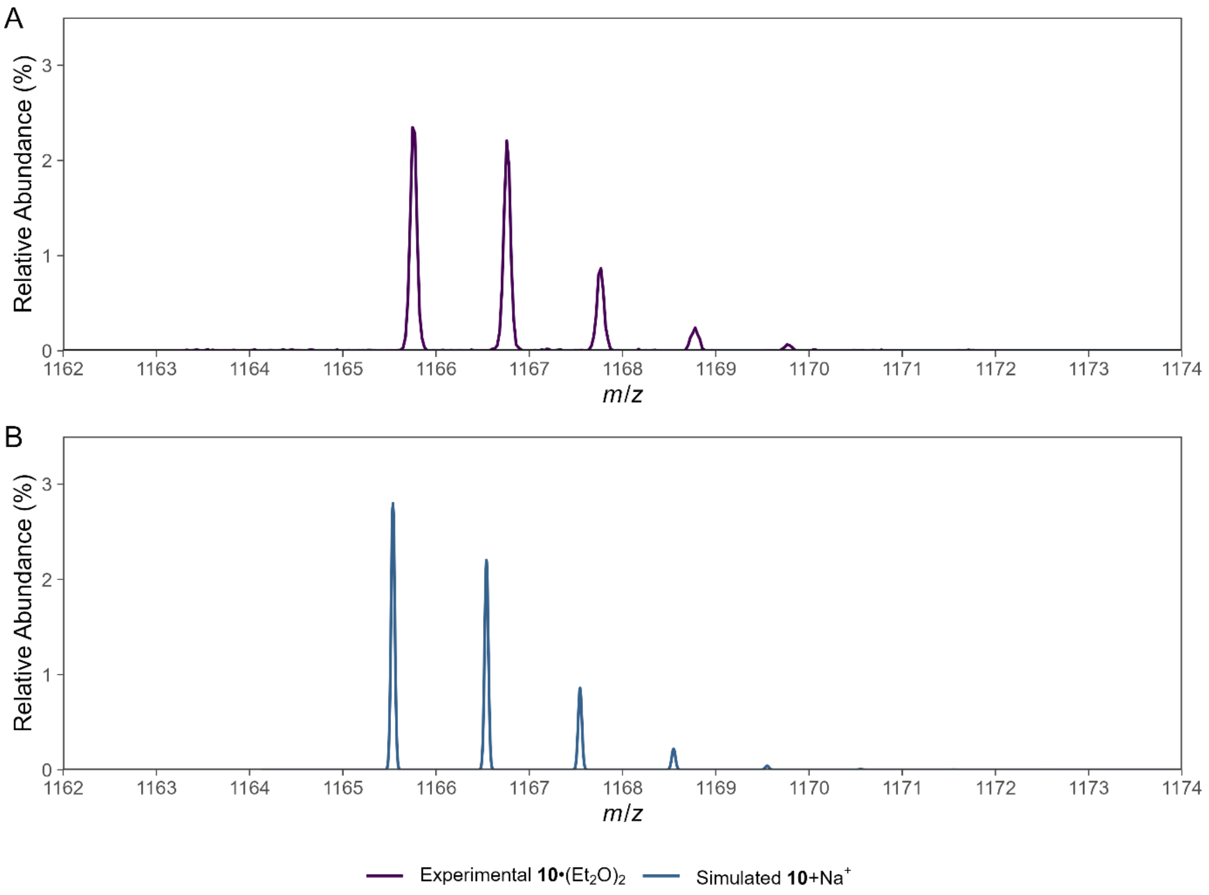

Figure S53. (A) Experimental ESI-MS spectrum for **10**•$(Et_2O)_2$. (B) Simulated ESI-MS spectrum for **10**+Na^+.

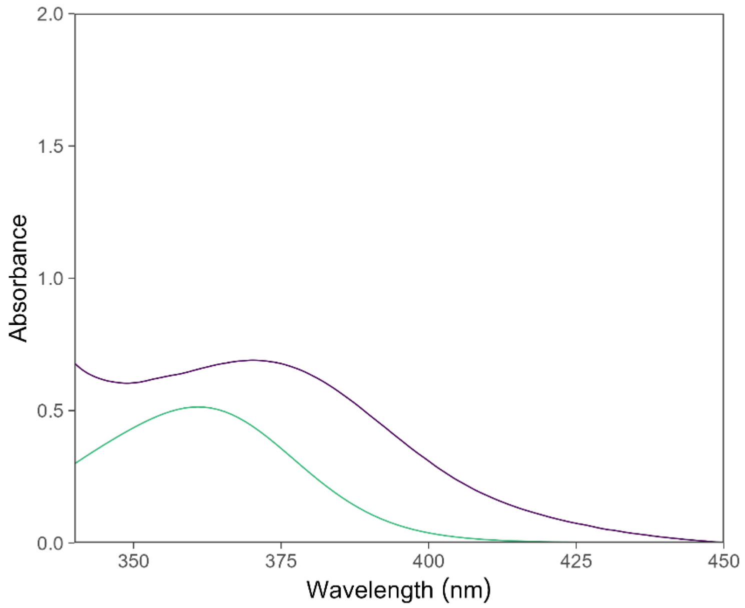

Figure S54. Experimental UV-Vis spectra of **10**•$(Et_2O)_2$ (92 µM) in benzene at room temperature. Simulated UV-Vis spectrum (DKH-PBE0/old-DKH-TZVPP) of ***E*-10*** (further information provided in Supplementary Table S14).

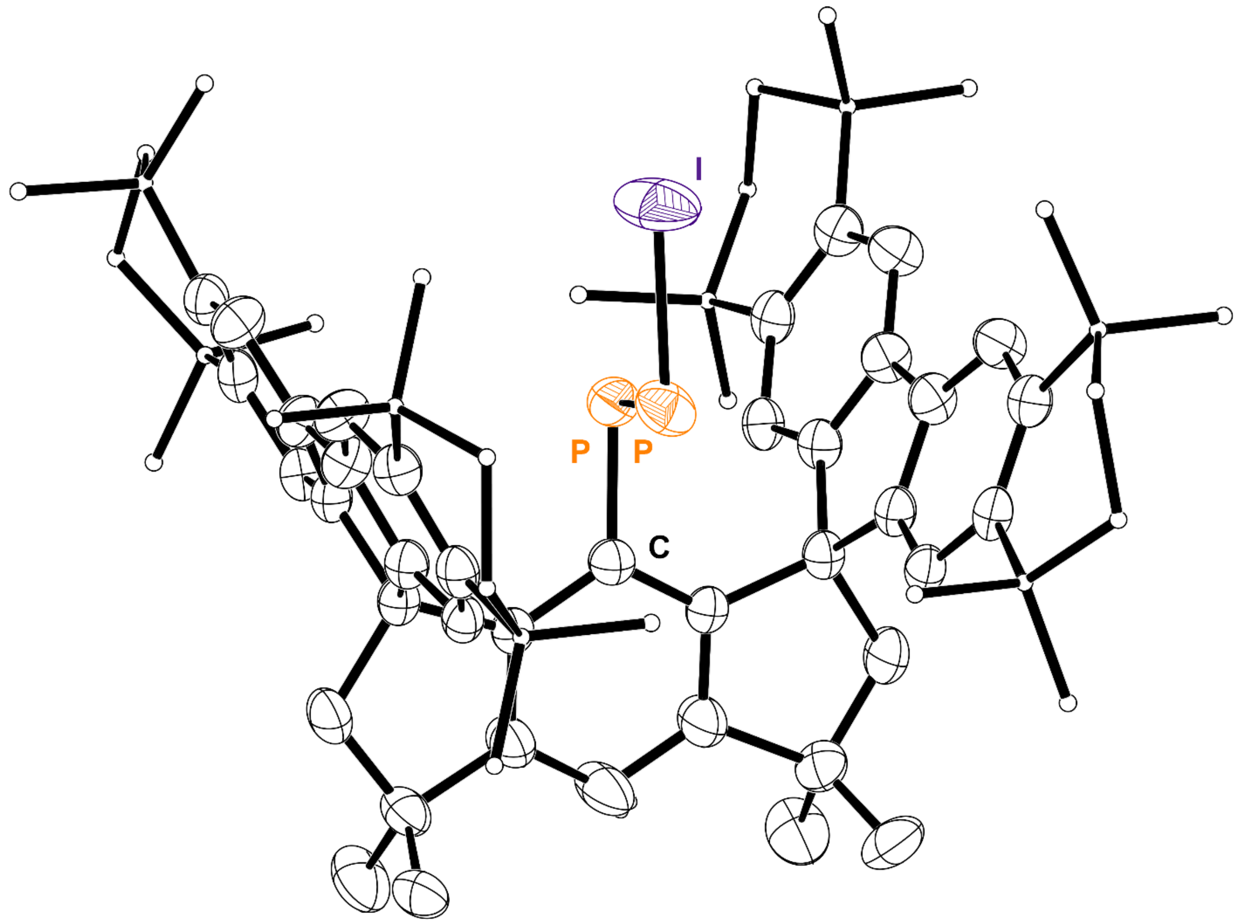

Figure S55. Thermal ellipsoid plot (50% probability) of **10**•$(Et_2O)_2$ (image showing the major *E* component). Solvent molecules, C-bound H atoms, and disordered components are omitted for clarity. Select C atoms are shown as spheres of arbitrary radius for clarity. Color code: P orange, C black, I purple.

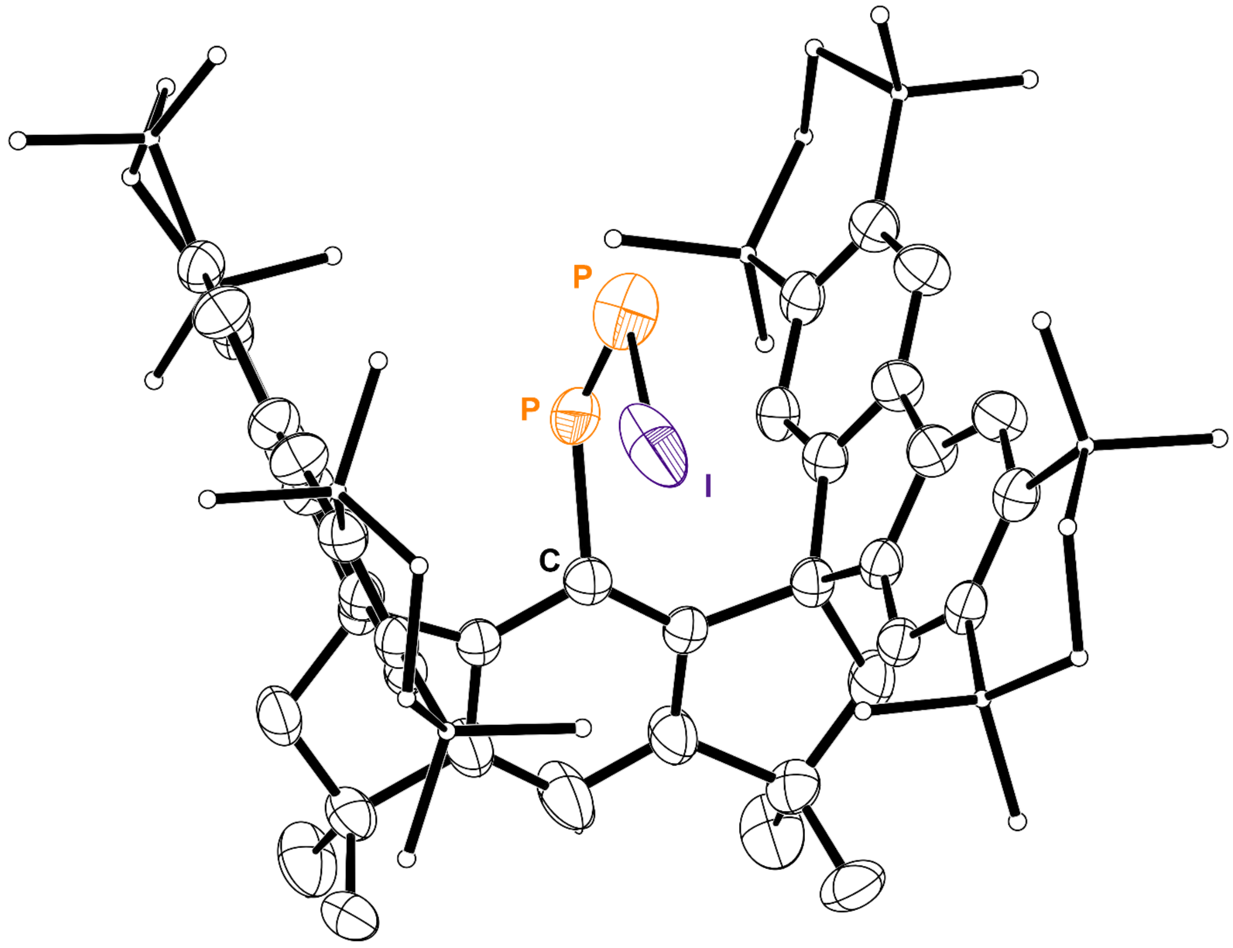

Figure S56. Thermal ellipsoid plot (50% probability) of **10**•$(Et_2O)_2$ (image showing the minor *Z* component). Solvent molecules, C-bound H atoms, and disordered components are omitted for clarity. Select C atoms are shown as spheres of arbitrary radius for clarity. Color code: P orange, C black, H grey, I purple.

2.8 Synthesis of [(M^{s}FluInd*)PPCl•Ag][CF_3SO_3]•(hexane) (11•(hexane))

A solution of **8**•(Et_2O)$_2$ (75 mg, 0.062 mmol) in toluene (1.2 mL) was added dropwise to a suspension of $AgCF_3SO_3$ (33 mg, 0.128 mmol) in toluene (4 mL) before being stirred at room temperature for 16 h under dark condition. The suspension was then filtered and the resulting orange filtrate was stripped of solvent to afford an orange powder. The residue was recrystallized from hexane at –30 °C to form an orange crystalline product that was dried under vacuum. Yield: 44 mg (50%). Crystals suitable for X-ray diffraction were grown from a concentrated solution of **11** in toluene/hexane/benzene.

Elemental analysis, Found: C, 60.25; H, 6.56%. **Calc.** for $C_{79}H_{103}AgClF_3O_3P_2S$: C, 68.02; H, 7.44%. *Elemental analysis of the silver complex, **11•(hexane)**, provided unexpectedly low results, possibly due to issues involving incomplete combustion of organometallic complexes or thermal decomposition or photodecomposition of organometallic silver complexes.*[42, 43]

ESI-MS (m/z) [11–CF_3SO_3]$^+$ 1157.530 (calc 1157.517).

^{1}H NMR (400 MHz, C_6D_6): δ = 8.05 (s, 4H), 7.32 (s, 1H), 7.19 (s, 4H), 2.48 (s, 4H), 1.61-1.45 (m, 30H), 1.33-1.21 (m, 36H), 1.16 (br s, 12H) ppm.

^{13}C{^{1}H} NMR (101 MHz, C_6D_6): δ = 173.5, 155.2, 145.9, 122.1, 118.7, 62.9, 56.2, 43.3, 35.2, 35.0, 33.1, 33.0, 32.7, 32.5, 32.4, 32.3, 32.1 ppm. Some expected resonances associated with aryl C atoms were not observed, presumably due to significant broadening.

^{31}P{^{1}H} NMR (162 MHz, C_6D_6): δ = 462.8 (br s), 351.3 (br s) ppm.

^{19}F{^{1}H} NMR (162 MHz, C_6D_6): δ = –76.8 ppm.

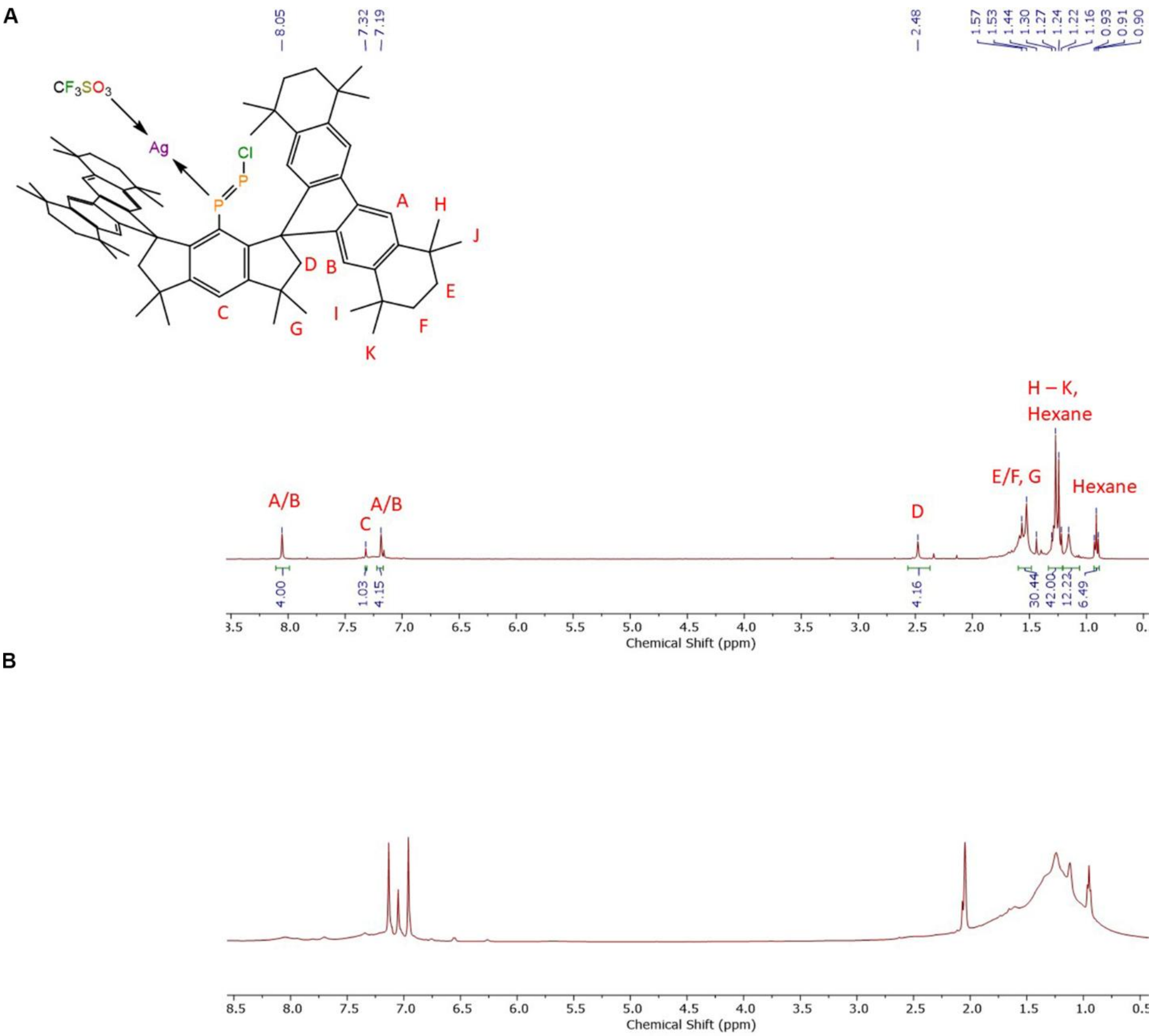

Figure S57. (A) ¹H NMR spectrum (C_6D_6, 400 MHz) of **11•**(hexane) at room temperature. (B) ¹H NMR spectrum (C_7D_8, 500 MHz) of **11•**(hexane) at –80 °C.

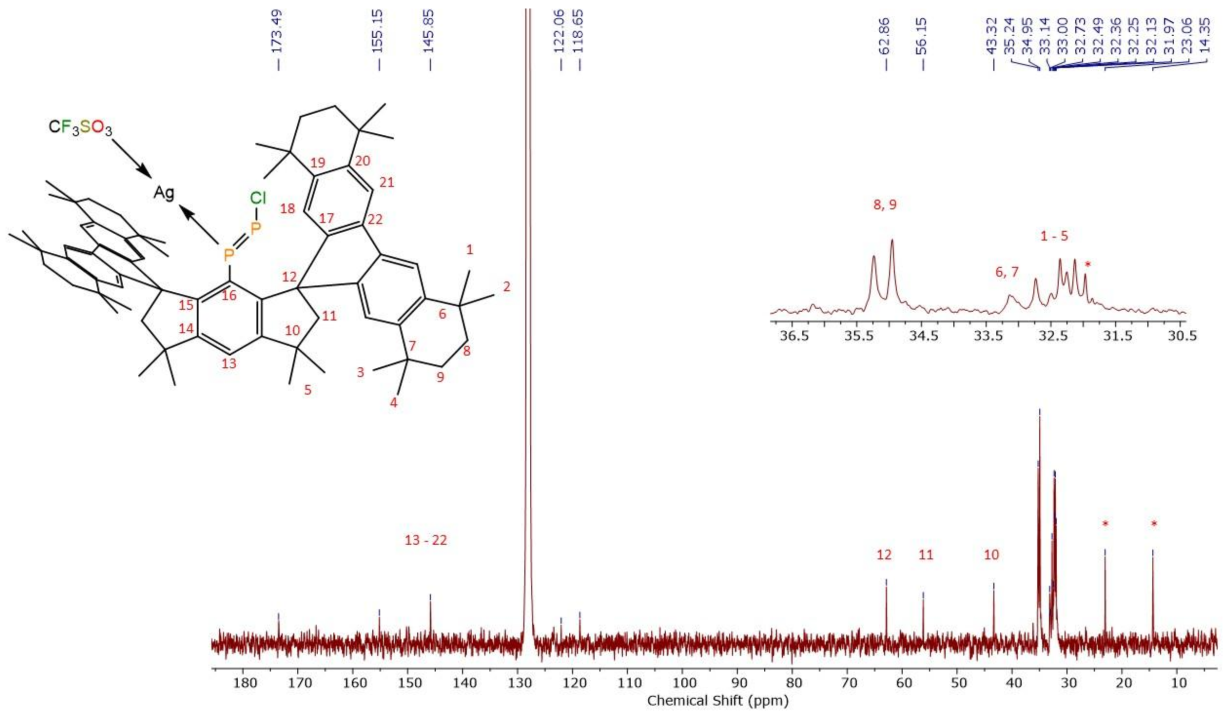

Figure S58. $^{13}C\{^1H\}$ NMR spectrum (C_6D_6, 101 MHz) of **11**•(hexane) at room temperature. An asterisk denotes a signal arising from the hexane solvate.

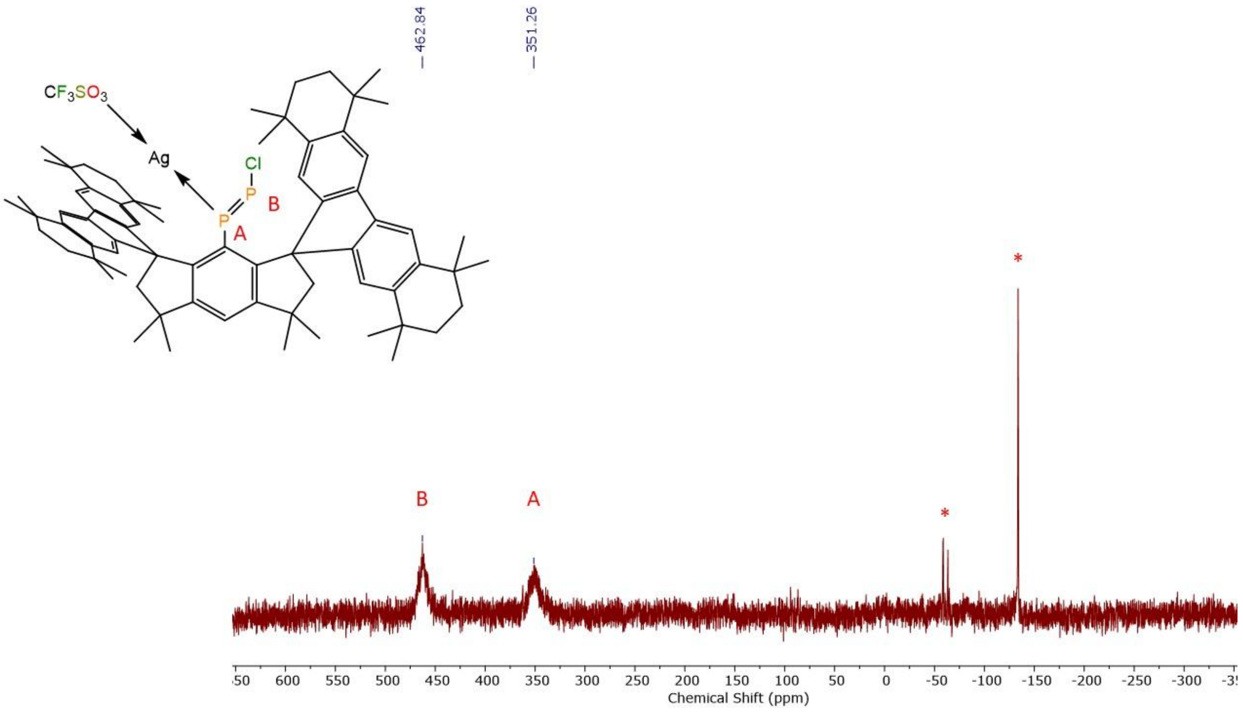

Figure S59. $^{31}P\{^1H\}$ NMR spectrum (C_6D_6, 162 MHz) of **11**•(hexane) at room temperature. An asterisk denotes a signal arising from trace impurity.

A

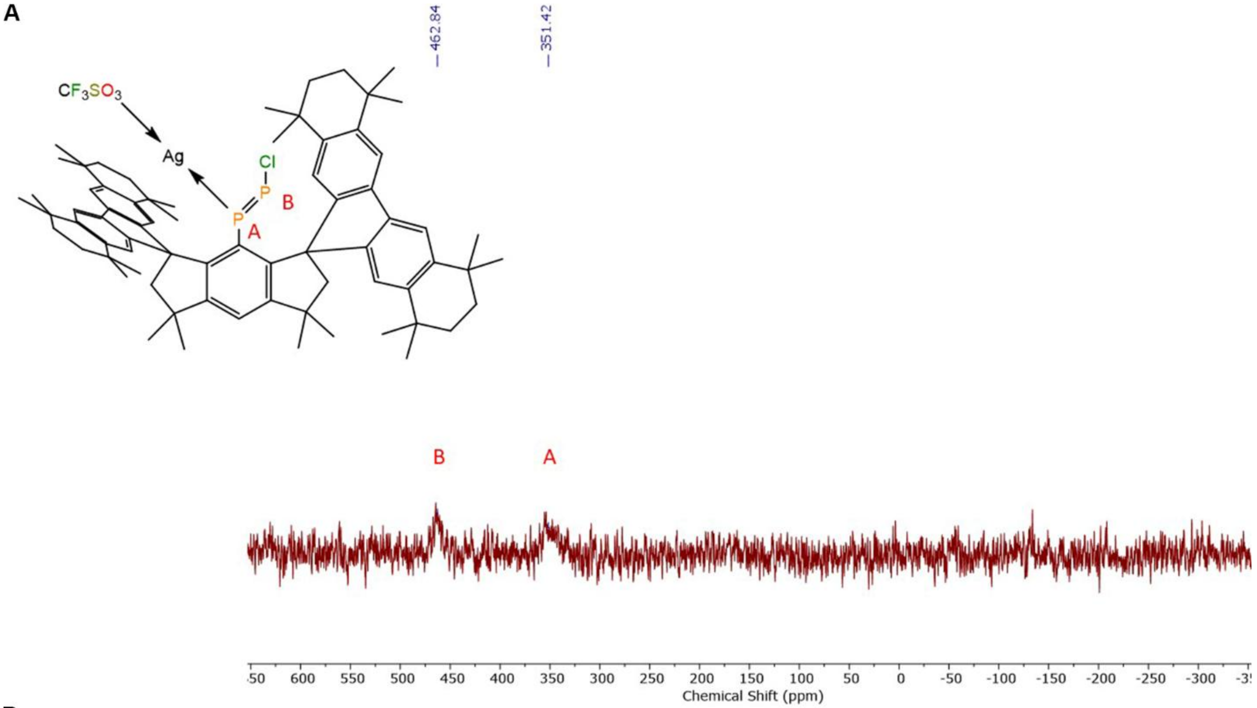

B

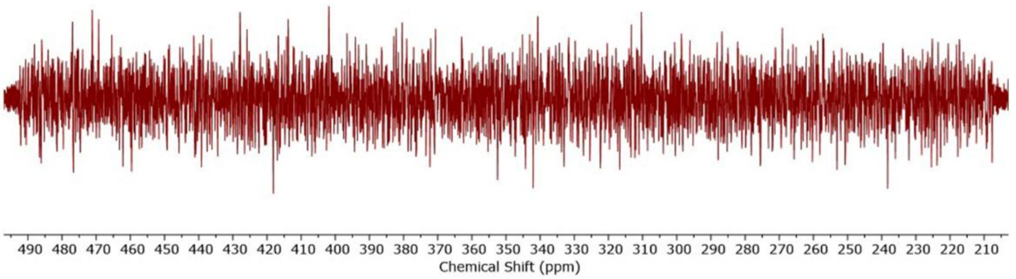

Figure S60. (A) ^{31}P NMR spectrum (C_6D_6, 162 MHz) of **11**•(hexane) at room temperature. (B) ^{31}P NMR spectrum (C_7D_8, 202 MHz) of **11**•(hexane) at –80 °C.

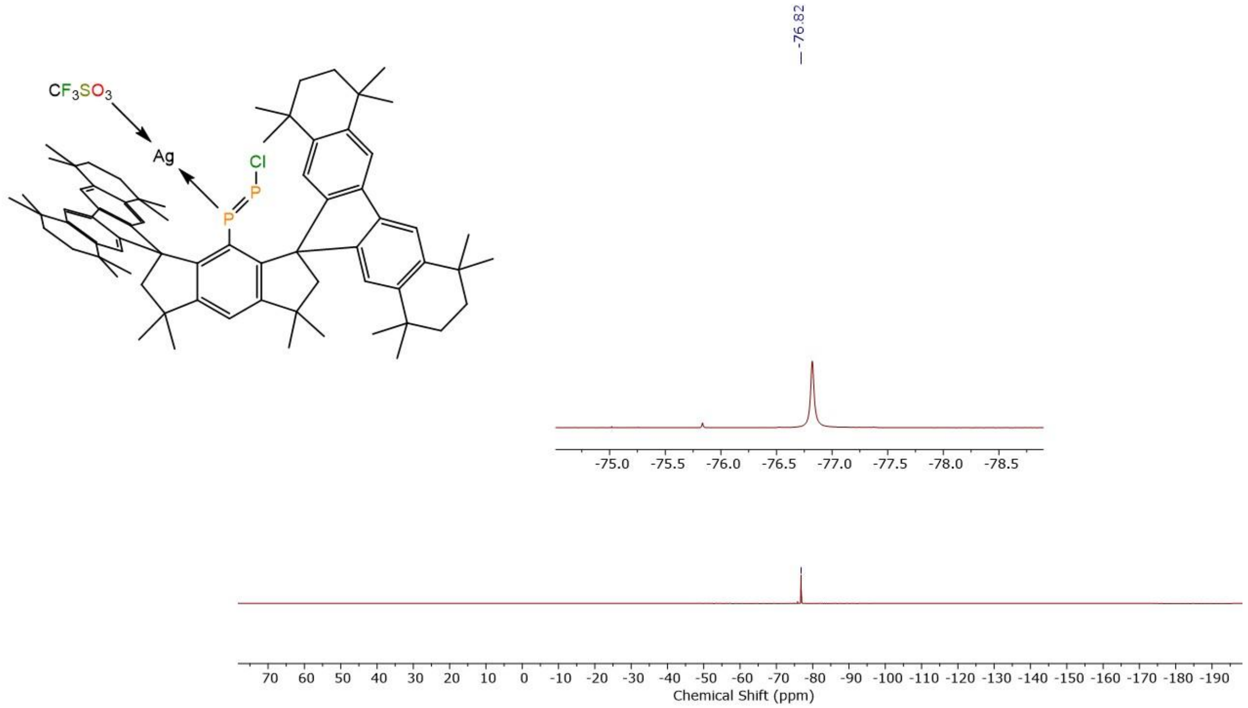

Figure S61. $^{19}F\{^1H\}$ NMR spectrum (C_6D_6, 376 MHz) of **11**•(hexane) at room temperature.

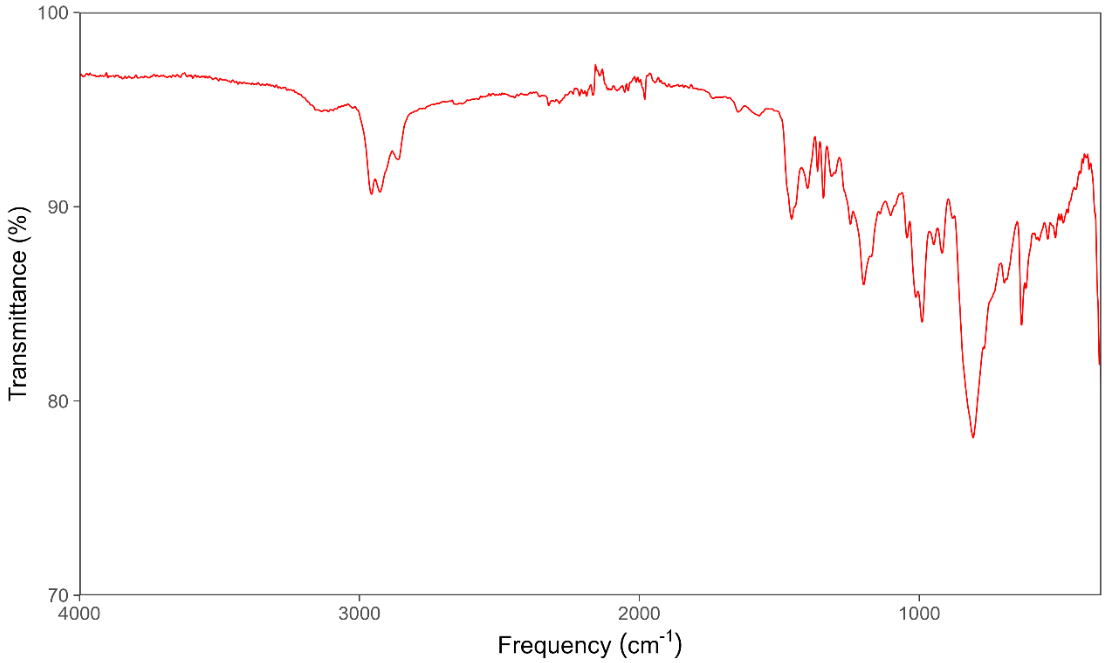

Figure S62. Experimental IR spectrum of **11**•(hexane).

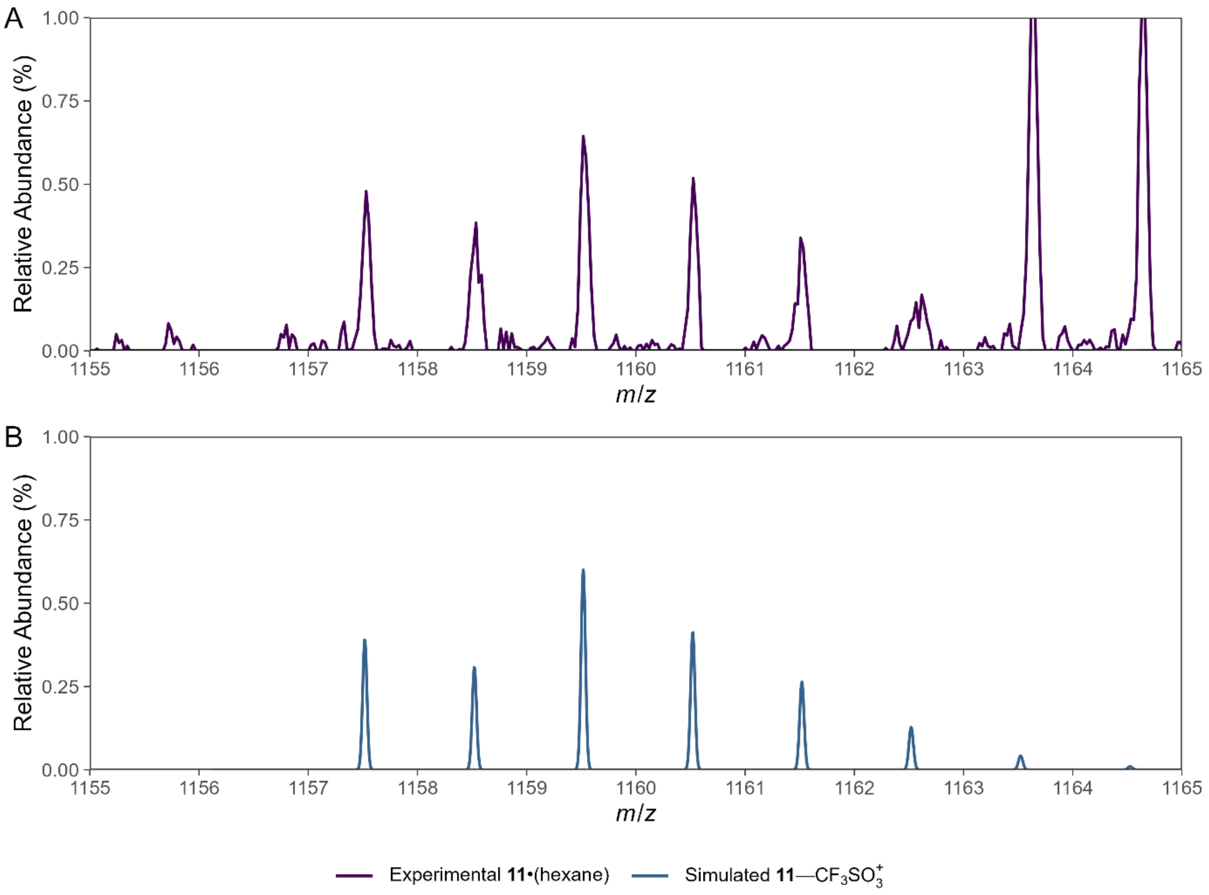

Figure S63. (A) Experimental ESI-MS spectrum for **11**•(hexane). (B) Simulated ESI-MS spectrum for **11**–$CF_3SO_3^+$.

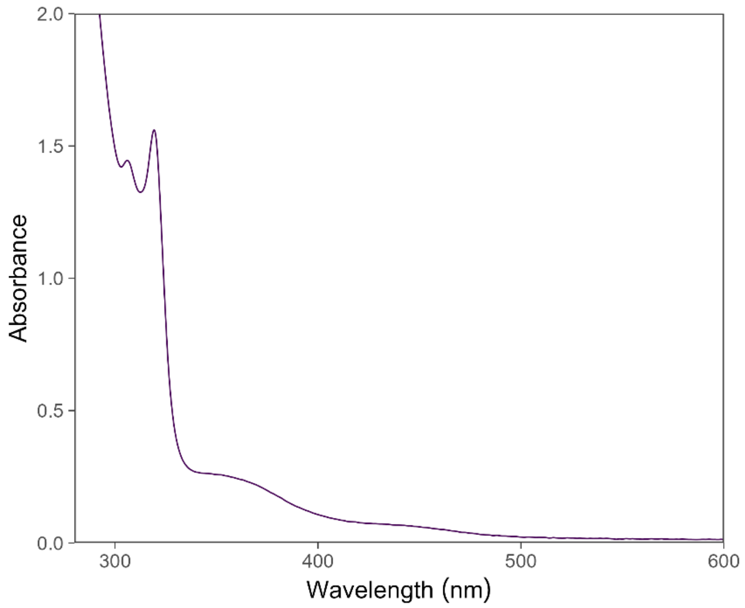

Figure S64. Experimental UV-Vis spectrum of **11**•(hexane) (59 μM) in benzene at room temperature.

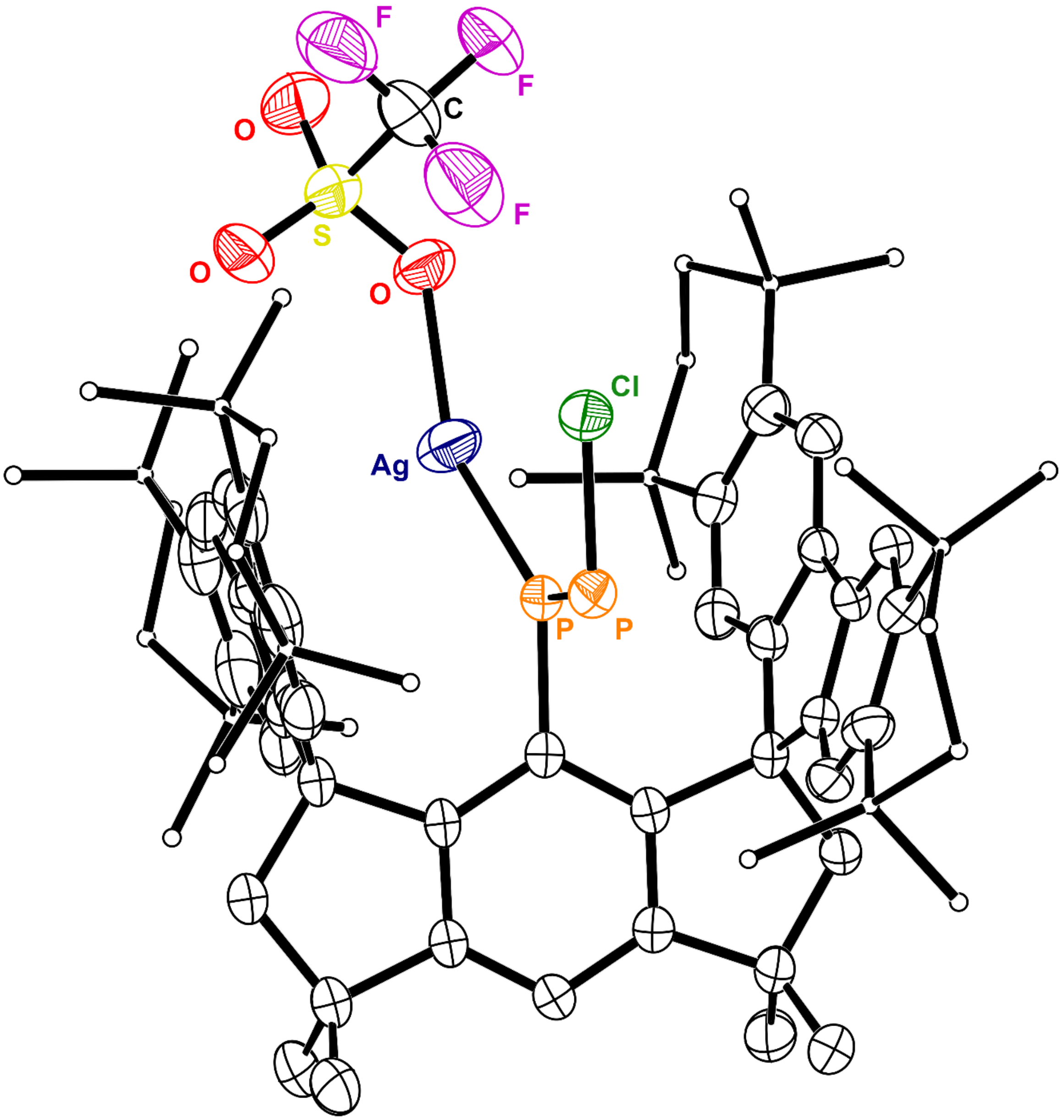

Figure S65. Thermal ellipsoid plot (50% probability) of **11** (image showing the major *E* component with respect to the arylhalodiphosphene ligand, **8**). Solvent molecules, C-bound H atoms, and disordered components are omitted for clarity. Select C atoms are shown as spheres of arbitrary radius for clarity. Color code: P orange, C black, Cl green, Ag navy, O red, S yellow, F pink.

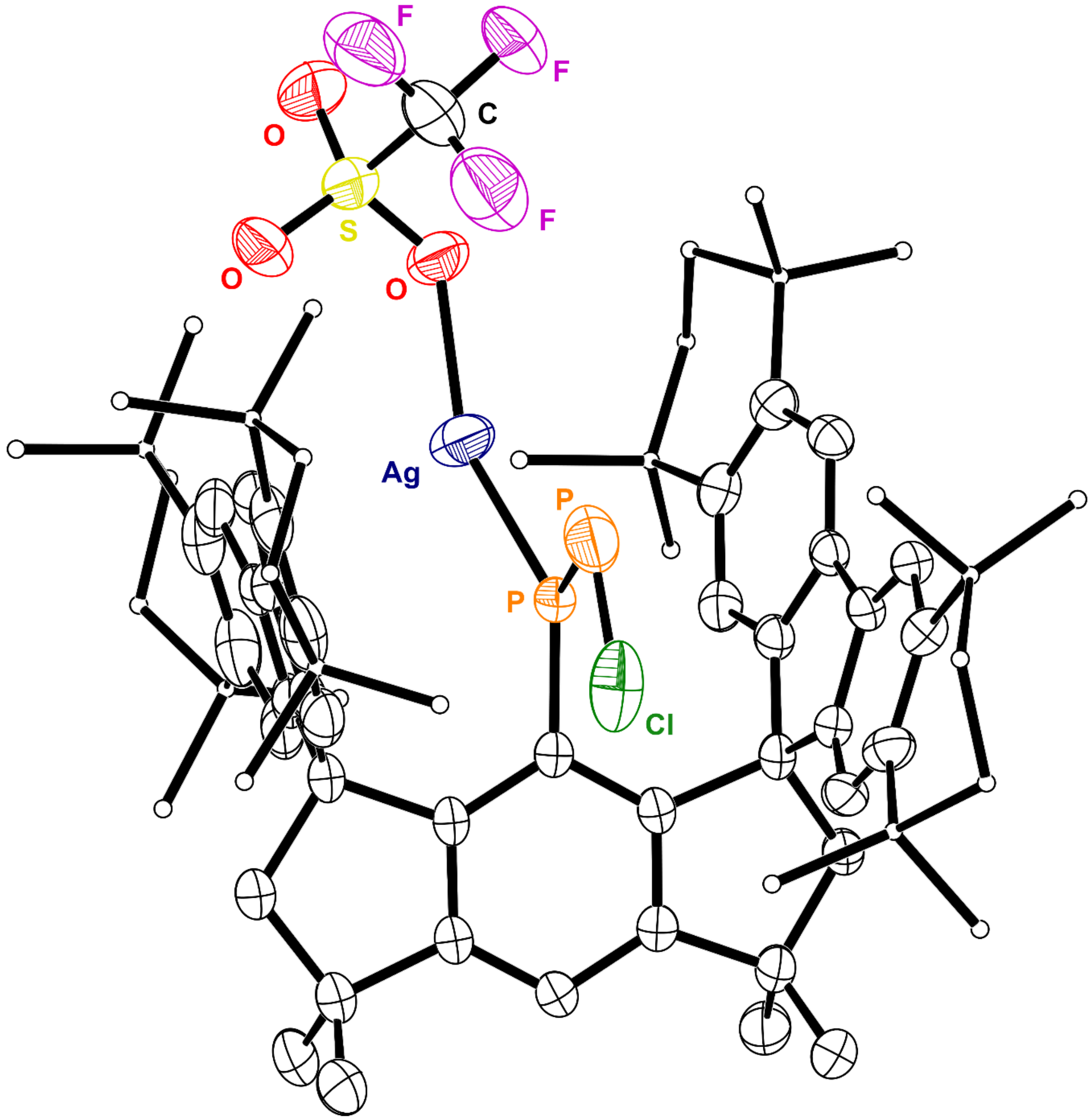

Figure S66. Thermal ellipsoid plot (50% probability) of **11** (image showing the minor *Z* component with respect to the arylhalodiphosphene ligand, **8**). Solvent molecules, C-bound H atoms, and disordered components are omitted for clarity. Select C atoms are shown as spheres of arbitrary radius for clarity. Color code: P orange, C black, Cl green, Ag navy, O red, S yellow, F pink.

2.9 Crystal growth of (M^{s}FluInd*)Li•(Et_2O)•(toluene)$_2$ (2•(Et_2O)•(toluene)$_2$).

A solution of *tert*-butyl lithium (1.7 M in pentane, 1.2 mL, 2.0 mmol) was added to a yellow suspension of **1** (0.400 g, 0.386 mmol) in Et_2O (5 mL) at –78 °C. The yellow solution darkened and was stirred for 15 min before being allowed to warm up to room temperature. As the mixture warmed, the solution reddened and a colorless precipitate formed. Volatiles were removed under vacuum. The solids were washed with Et_2O (3 × 4 mL) before being extracted with toluene. The mixture was filtered through glass filter paper into a vial and colorless crystals formed as the toluene evaporated*.* ***2***•*(Et_2O)•(toluene)$_2$ is highly sensitive and was not isolated as a bulk material or subject to further characterization.*

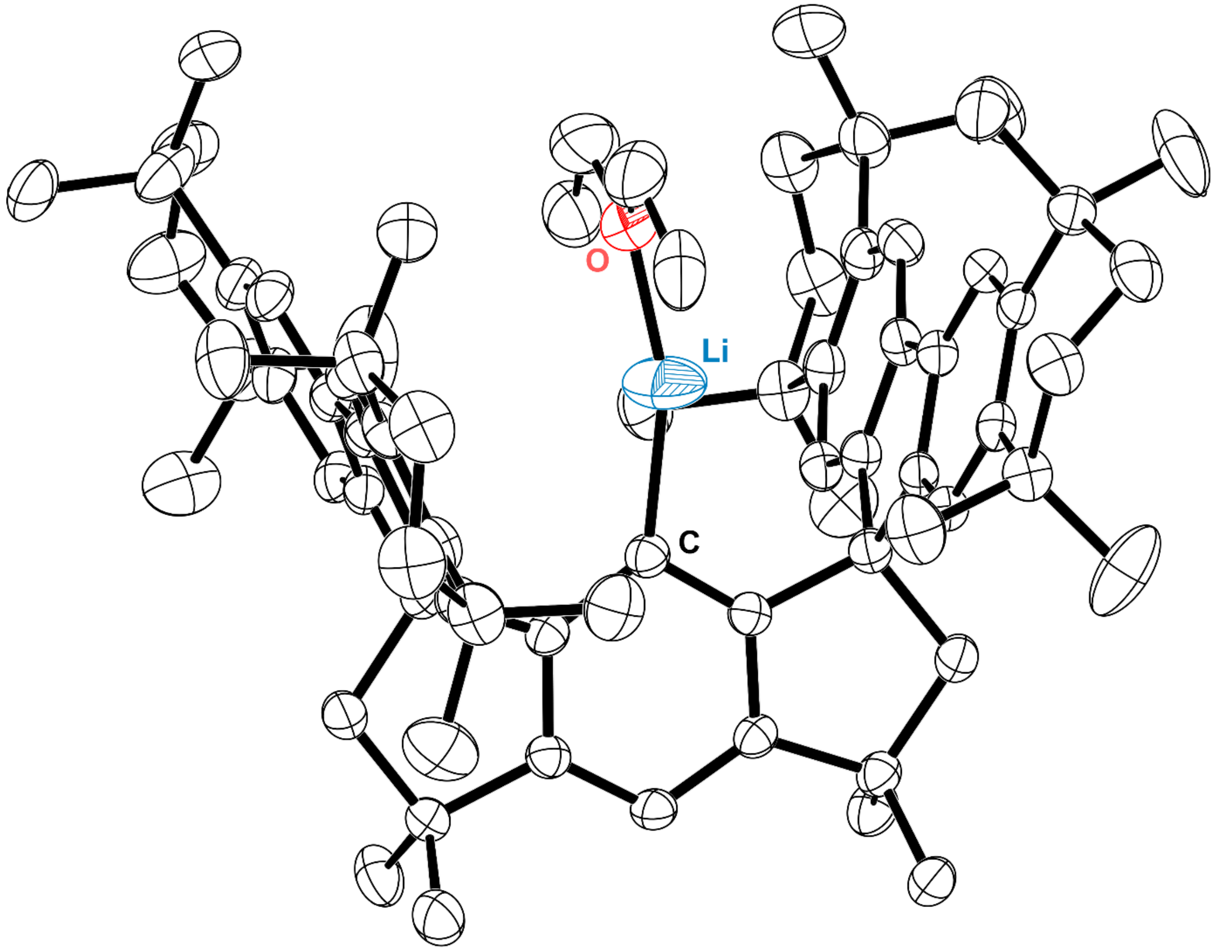

Figure S67. Thermal ellipsoid plot (50% probability) of **2**•(Et_2O)•(toluene)$_2$. Solvent molecules, H atoms, and disordered components are omitted for clarity. Color code: Li light blue, O red, C black.

2.10 Crystal growth ($M^sFluInd^*$)PHK•(toluene)$_{2.5}$ (5•(toluene)$_{2.5}$).

A solution of **4**•(hexane) (49 mg, 0.050 mmol) in benzene (2 mL) was added to a red suspension of KBz (9.0 mg, 0.07 mmol) in benzene (0.6 mL) and stirred at room temperature for 1 h. The resulting red solution was filtered through glass filter paper and solvent removed under reduced pressure to give a red powder. This red powder was dissolved in hexane, which was transferred to a crystallization vial, from which the hexane could diffuse out into an outer pool of toluene. Large red block crystals formed overnight at room temperature, and one crystal was analyzed by SC-XRD to determine the solid-state structure of **5**•(toluene)$_{2.5}$. ***5***•*(toluene)$_{2.5}$ is highly sensitive and was not isolated as a bulk material or subject to further characterization.*

.

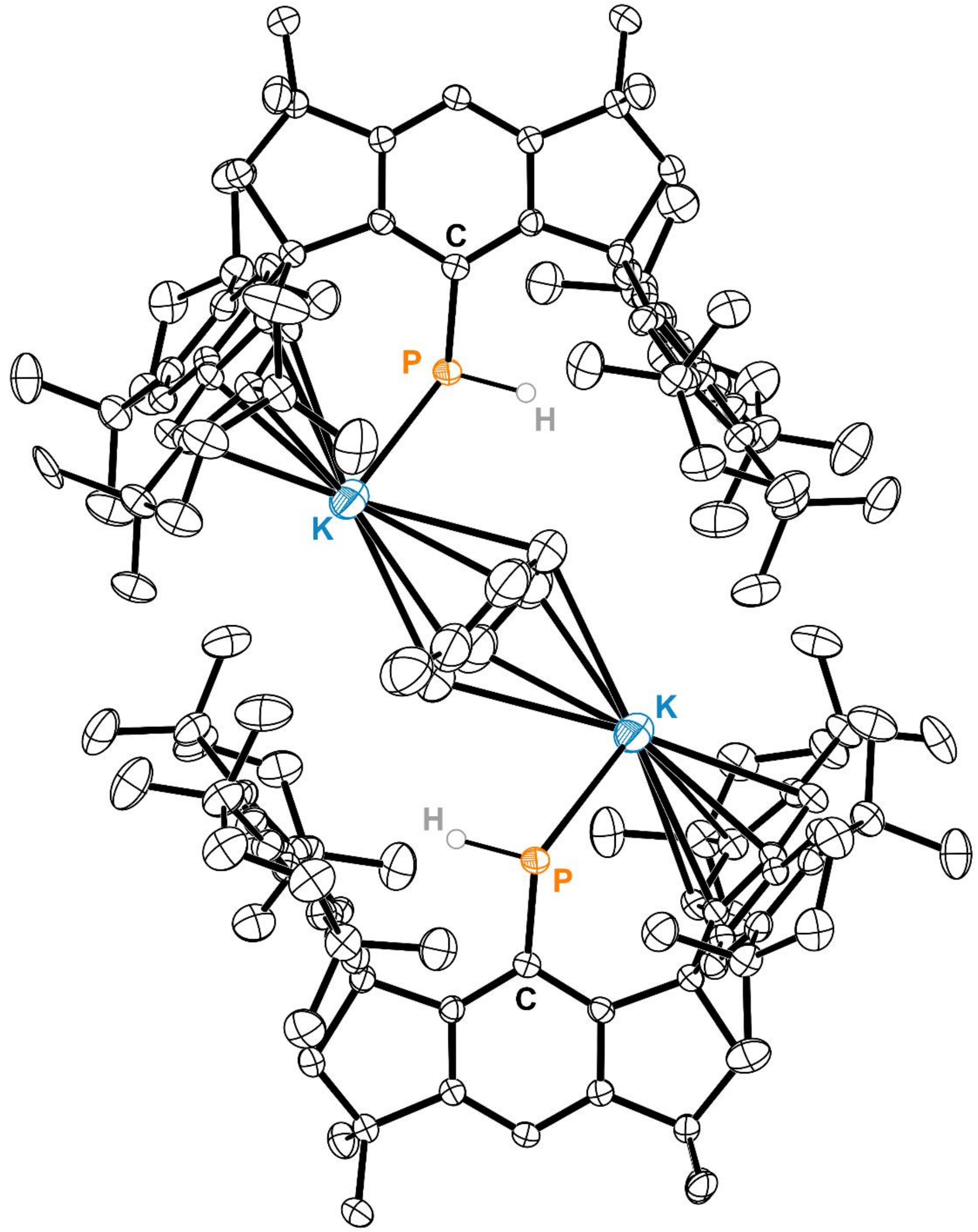

Figure S68. Thermal ellipsoid plot (50% probability) of **5**•(toluene)$_{2.5}$. Solvent molecules and disordered components are omitted for clarity. Color code: P orange, C black, H grey, K light blue.

2.11 Protonolysis of 8•(Et_2O)$_2$.

A solution of **8**•(Et_2O)$_2$ (10 mg, 8.3 μmol) in benzene (0.6 mL) was treated with 48% HBr in H_2O (3.0 μL, 26.6 mmol) and heated to 70 °C for 1 h. The solution lost its characteristic yellow color, and NMR analysis revealed the formation of **4**, generated *in situ*.

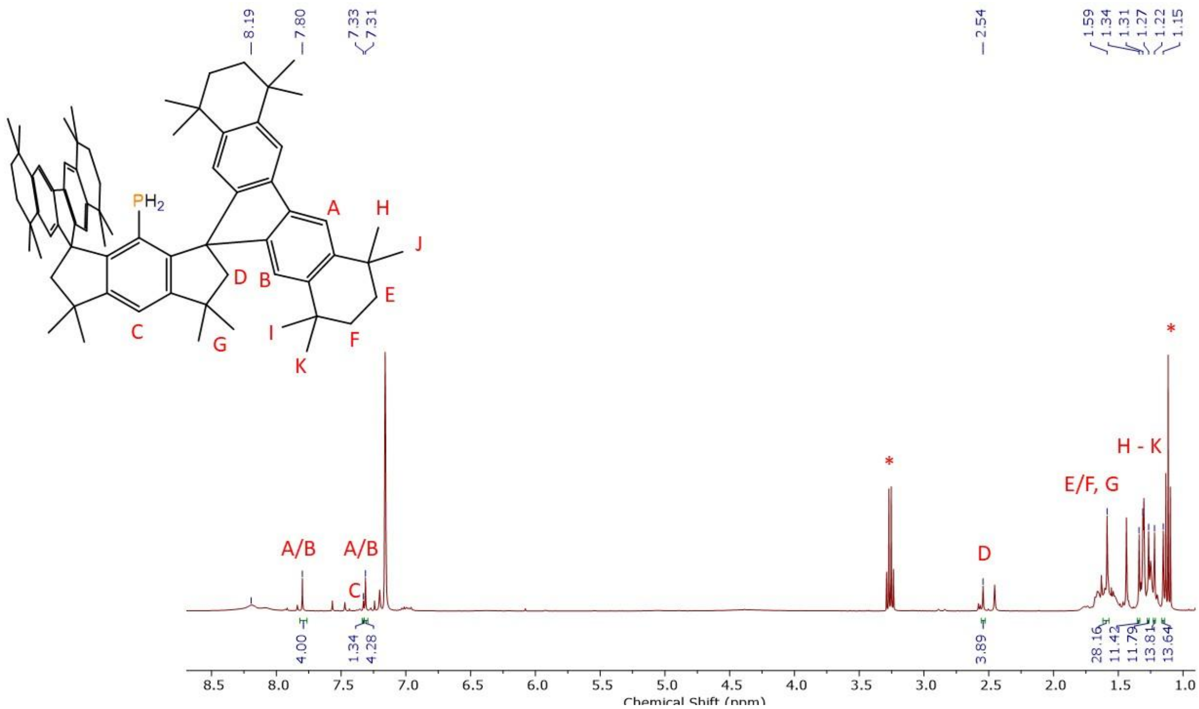

Figure S69. ^1H NMR spectrum (C_6D_6, 400 MHz) of a mixture of **8**•(Et_2O)$_2$ and HBr (48% in water) at room temperature. An asterisk denotes a signal arising from the ether solvate.

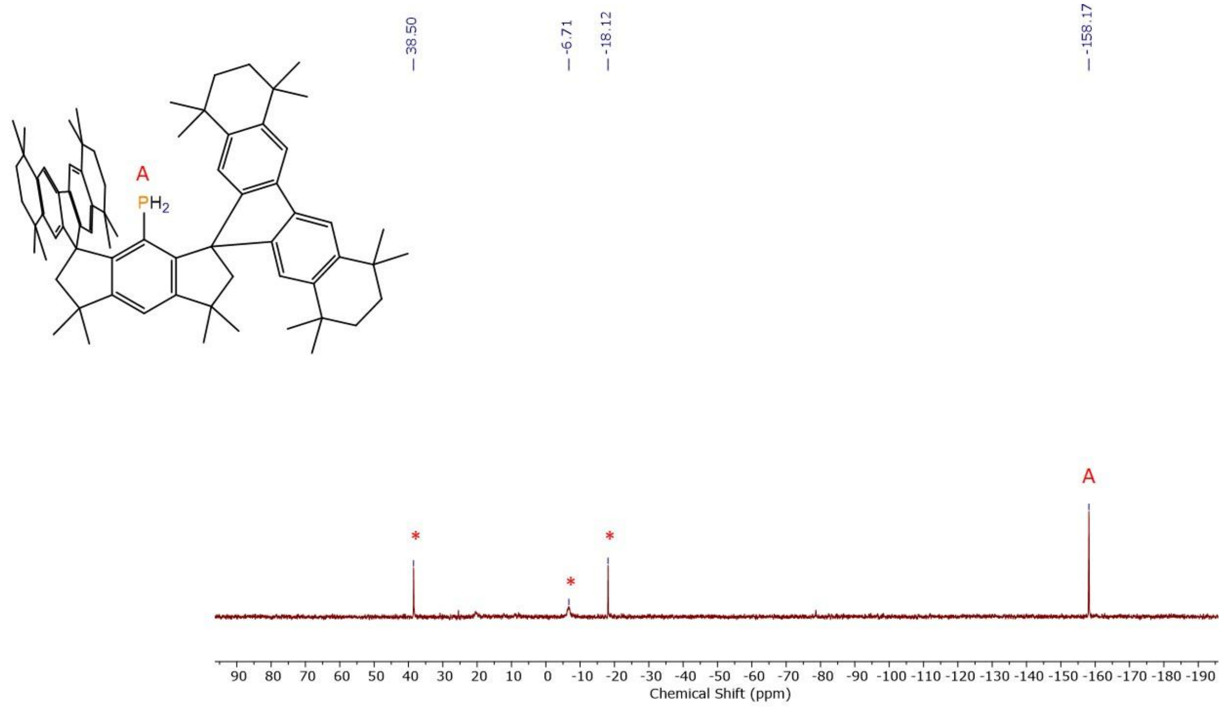

Figure S70. $^{31}P\{^{1}H\}$ NMR spectrum (C_6D_6, 162 MHz) of a mixture of **8**•$(Et_2O)_2$ and HBr (48% in water) at room temperature. An asterisk denotes a signal arising from an unidentified byproduct.

2.12 Treatment of 8•(Et_2O)$_2$ with halogen-abstraction reagents

A solution of **8**•(Et_2O)$_2$ (20 mg, 17 µmol) in C_7D_8 (0.6 mL) was treated with either $GaCl_3$ (2.9 mg, 17 µmol), $AlCl_3$ (2.2 mg, 17 µmol), or TMS(CF_3SO_3) (170 mg, 765 µmol). The reaction mixture was transferred to a J. Young-type NMR tube and heated to 100 °C for 16 h. In each case, ^{31}P NMR analysis confirms the presence of unreacted **8** in solution.

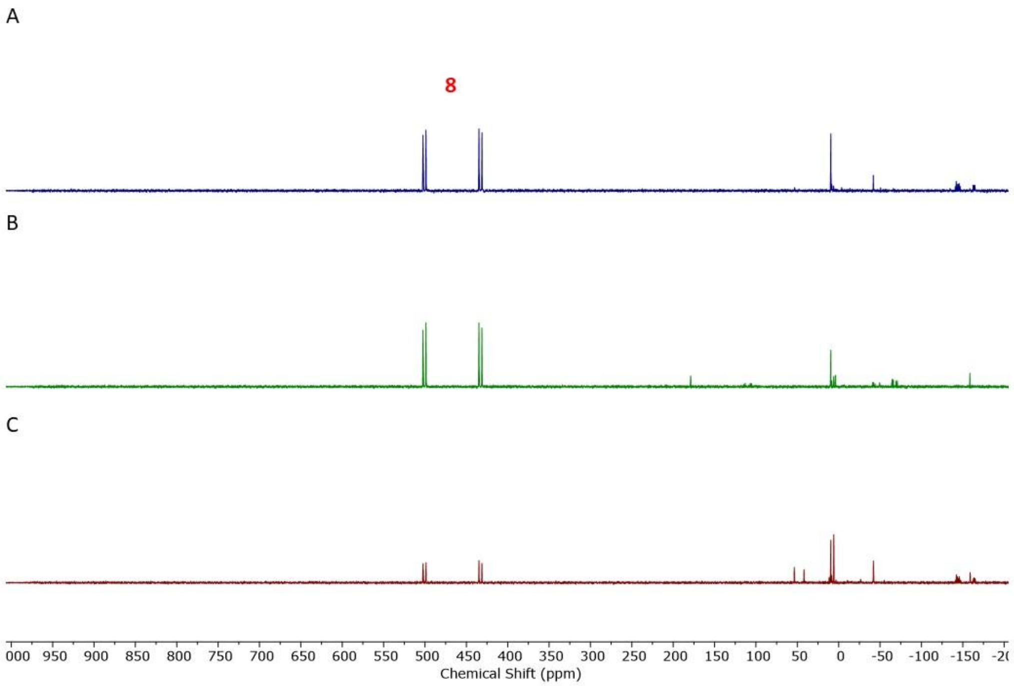

Figure S71. Stacked $^{31}P\{^1H\}$ NMR spectra (C_7D_8, 162 MHz) of (A) a mixture of **8**•(Et_2O)$_2$ and $GaCl_3$, (B) a mixture of **8**•(Et_2O)$_2$ and $AlCl_3$, and (C) a mixture of **8**•(Et_2O)$_2$ and TMS(CF_3SO_3) at room temperature. The mixtures were heated at 100 °C for 16 h prior to spectra acquisition.

2.13 Treatment of 8 with halogen-abstraction reagents

A solution of **8** (20 mg, 19 µmol) in C_7D_8 (0.6 mL) was treated with either $GaCl_3$ (3.3 mg, 19 µmol) or $AlCl_3$ (2.5 mg, 19 µmol). In each case, ^{31}P NMR analysis reveals the presence of multiple inseparable reaction products.

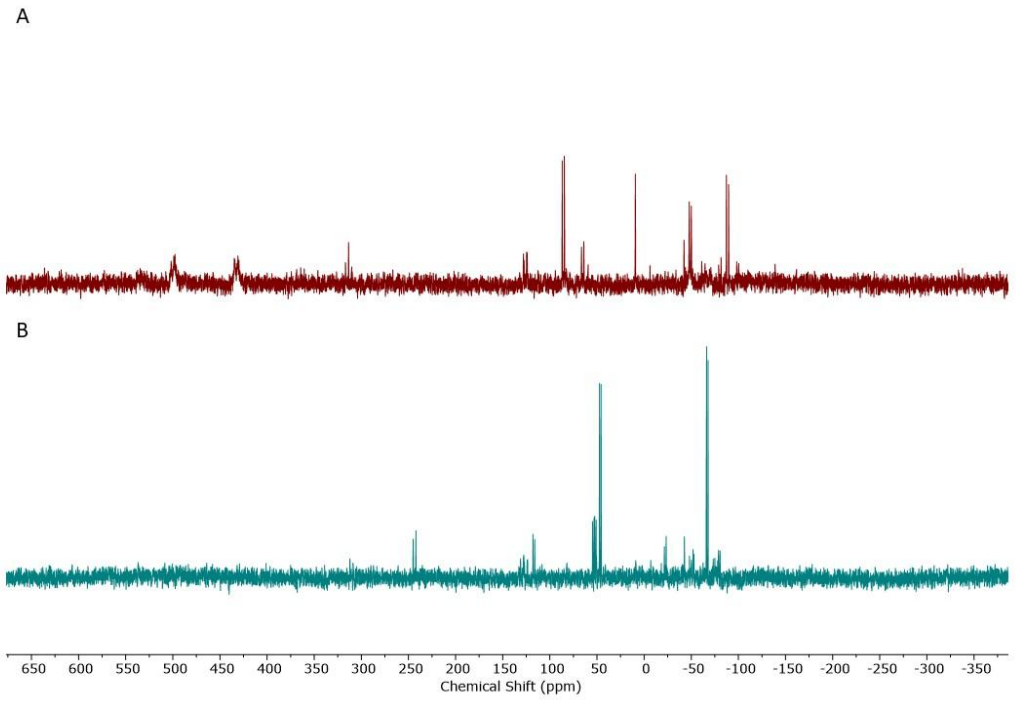

Figure S72. Stacked $^{31}P\{^1H\}$ NMR spectra (C_7D_8, 162 MHz) of (A) a mixture of **8** and $GaCl_3$ and (B) a mixture of **8** and $AlCl_3$ at room temperature.

3. Crystallographic Tables

Table S1. Crystallographic details for **2**•(Et_2O)•(toluene)$_2$, **3**•(hexane), and **4**•(hexane).

**Compound**	**2**• (Et_2O)• (toluene)$_2$	**3**• (hexane)	**4**• (hexane)
**Empirical formula**	$C_{90}H_{115}LiO$	$C_{78}H_{103}Cl_2P$	$C_{78}H_{105}P$
**Formula Weight**	1219.75	1142.47	1073.58
**Temperature (K)**	99.95(18)	100.01(10)	100.00(10)
**Wavelength (Å)**	1.54184	1.54184	1.54184
**Crystal system**	Monoclinic	Monoclinic	Monoclinic
**Space group**	$P2_1/n$	$P2_1/c$	$P2_1/c$
a **(Å)**	12.50871(7)	12.40239(7)	12.46887(5)
b **(Å)**	29.33042(12)	26.34880(17)	26.12266(10)
c **(Å)**	20.62954(9)	20.88177(13)	20.84307(9)
**α (°)**			
**β (°)**	93.8228(5)	101.4906(5)	102.0641(4)
**γ (°)**			
**Volume (Å^3)**	7551.84(6)	6687.14(7)	6639.06(5)
Z	4	4	4
**ρ_{calc} (Mg/m^3)**	1.073	1.135	1.074
**Crystal size (mm^3)**	0.23 × 0.14 × 0.12	0.09 × 0.07 × 0.04	0.12 × 0.09 × 0.08
**θ range (°)**	2.623 to 67.072	2.734 to 75.938	2.750 to 74.504
**Total reflections**	113737	100468	300587
**Unique reflections**	13460	13731	13589
**Parameters**	1032	780	760
**Completeness**	99.9	100.0	100.0
**R_{int}**	0.0244	0.0460	0.0389
**R_1 (I > 2σ)**	0.0623	0.0543	0.0398

**R_1 (all data)**	0.0671	0.0658	0.0448
**wR_2 (I > 2σ)**	0.1567	0.1504	0.0956
**wR_2 (all data)**	0.1597	0.1585	0.0985
**Goodness of fit, *S***	1.073	1.033	1.022
**Deposition Number (CCDC)**	2501235	2501236	2501237

Table S2. Crystallographic details for **5** •$(toluene)_{2.5}$, **6**•$(Et_2O)_2$, and **7**•$(Et_2O)_2$.

**Compound**	**5**• $(toluene)_{2.5}$	**6**• $(Et_2O)_2$	**7**• $(Et_2O)_2$
**Empirical formula**	$C_{89.50}H_{110}KP$	$C_{83}H_{119}O_2PSi$	$C_{80}H_{110}Cl_2O_2P_2$
**Formula Weight**	1255.84	1207.83	1236.51
**Temperature (K)**	150.00(10)	149.97(13)	100.00(10)
**Wavelength (Å)**	1.54184	1.54184	1.54184
**Crystal system**	Orthorhombic	Tetragonal	Tetragonal
**Space group**	*Pbca*	$P\bar{4}2_1m$	$P\bar{4}2_1m$
***a* (Å)**	22.66718(11)	17.70963(15)	17.68289(7)
***b* (Å)**	25.26153(15)		
***c* (Å)**	25.92072(16)	11.8110(2)	11.48603(9)
**α (°)**			
**β (°)**			
**γ (°)**			
**Volume ($Å^3$)**	14842.40(15)	3704.30(9)	3591.51(4)
Z	8	2	2
**$ρ_{calc}$ (Mg/m^3)**	1.124	1.083	1.143
**Crystal size (mm^3)**	0.23 × 0.18 × 0.06	0.216 × 0.17 × 0.103	0.164 × 0.128 × 0.102
**θ range (°)**	3.125 to 76.179	3.529 to 76.094	3.535 to 76.009

**Total reflections**	52114	62152	142958
**Unique reflections**	15356	4054	3922
**Parameters**	1018	304	364
**Completeness**	99.9	99.9	100.0
**R_{int}**	0.0305	0.0452	0.0398
**R_1 (I > 2σ)**	0.0616	0.0358	0.0480
**R_1 (all data)**	0.0704	0.0380	0.0509
**wR_2 (I > 2σ)**	0.1804	0.1027	0.1337
**wR_2 (all data)**	0.1887	0.1045	0.1368
**Goodness of fit, *S***	1.070	1.075	1.056
**Deposition Number (CCDC)**	2501238	2501239	2501240

Table S3. Crystallographic details for **8**•$(Et_2O)_2$, **9**•$(Et_2O)_2$, and **10**•$(Et_2O)_2$.

**Compound**	**8**• $(Et_2O)_2$	**9**• $(Et_2O)_2$	**10**• $(Et_2O)_2$
**Empirical formula**	$C_{80}H_{109}ClO_2P_2$	$C_{80}H_{109}BrO_2P_2$	$C_{80}H_{109}IO_2P_2$
**Formula Weight**	1200.06	1244.52	1291.51
**Temperature (K)**	100.00(10)	100.00(10)	99.9(4)
**Wavelength (Å)**	1.54184	1.54184	1.54184
**Crystal system**	Tetragonal	Tetragonal	Tetragonal
**Space group**	$P\bar{4}2_1m$	$P\bar{4}2_1m$	$P\bar{4}2_1m$
***a* (Å)**	17.64558(10)	17.64070(10)	17.63400(10)
***b* (Å)**			
***c* (Å)**	11.44934(11)	11.47390(10)	11.51880(10)
**α (°)**			
**β (°)**			
**γ (°)**			

**Volume (Å^3)**	3564.94(5)	3570.61(5)	3581.86(5)
Z	2	2	2
**ρ_{calc} (Mg/m^3)**	1.118	1.158	1.197
**Crystal size (mm^3)**	0.145 × 0.143 × 0.096	0.15 × 0.09 × 0.05	0.091 × 0.075 × 0.047
**θ range (°)**	3.542 to 74.504	3.543 to 74.474	3.545 to 75.941
**Total reflections**	77827	80005	73101
**Unique reflections**	3802	3831	3923
**Parameters**	340	351	326
**Completeness**	99.9	100.0	99.9
**R_{int}**	0.0413	0.0435	0.0486
**R_1 (I > 2σ)**	0.0462	0.0453	0.0568
**R_1 (all data)**	0.0490	0.0462	0.0580
**wR_2 (I > 2σ)**	0.1311	0.1278	0.1636
**wR_2 (all data)**	0.1344	0.1286	0.1648
**Goodness of fit, *S***	1.038	1.043	1.063
**Deposition Number (CCDC)**	2501241	2512823	2512824

Table S4. Crystallographic details for **11**.

**Compound**	**11**
**Empirical formula**	$C_{73}H_{89}AgClF_3O_3P_2S$
**Formula Weight**	1308.76
**Temperature (K)**	100.00(10)
**Wavelength (Å)**	1.54184
**Crystal system**	Monoclinic
**Space group**	$P2_1/n$
***a* (Å)**	15.8885(2)

b **(Å)**	27.6598(3)
c **(Å)**	18.0802(2)
**α (°)**	
**β (°)**	105.3380(10)
**γ (°)**	
**Volume ($Å^3$)**	7662.74(16)
Z	4
**ρ_{calc} (Mg/m^3)**	1.134
**Crystal size (mm^3)**	0.14 × 0.14 × 0.08
**θ range (°)**	2.996 to 75.967
**Total reflections**	90526
**Unique reflections**	15794
**Parameters**	917
**Completeness**	99.9
**R_{int}**	0.0417
**R_1 ($I > 2\sigma$)**	0.0541
**R_1 (all data)**	0.0680
**wR_2 ($I > 2\sigma$)**	0.1355
**wR_2 (all data)**	0.1425
**Goodness of fit, *S***	1.044
**Deposition Number (CCDC)**	2523752

4. Computational Data

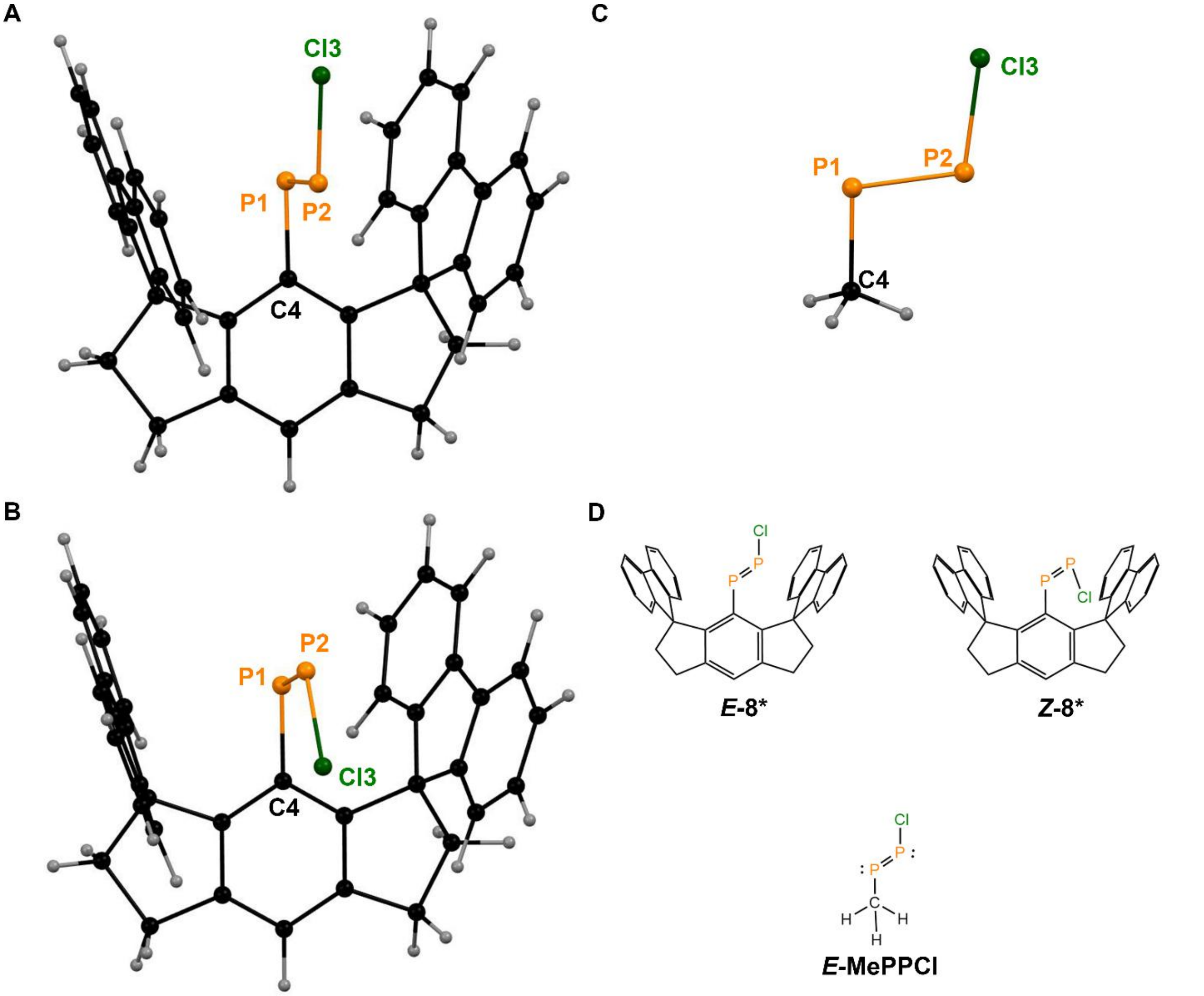

Figure S73. Ball-and-stick representation of geometry-optimized atomic coordinates (PBE0-D3/def2-TZVPP) of (A) ***E*-8***, (B) ***Z*-8***, and (C) ***E*-MePPCl**. (D) Diagrams of ***E*-8***, ***Z*-8***, and ***E*-MePPCl**. Color code: P orange, C black, Cl dark green, H grey.

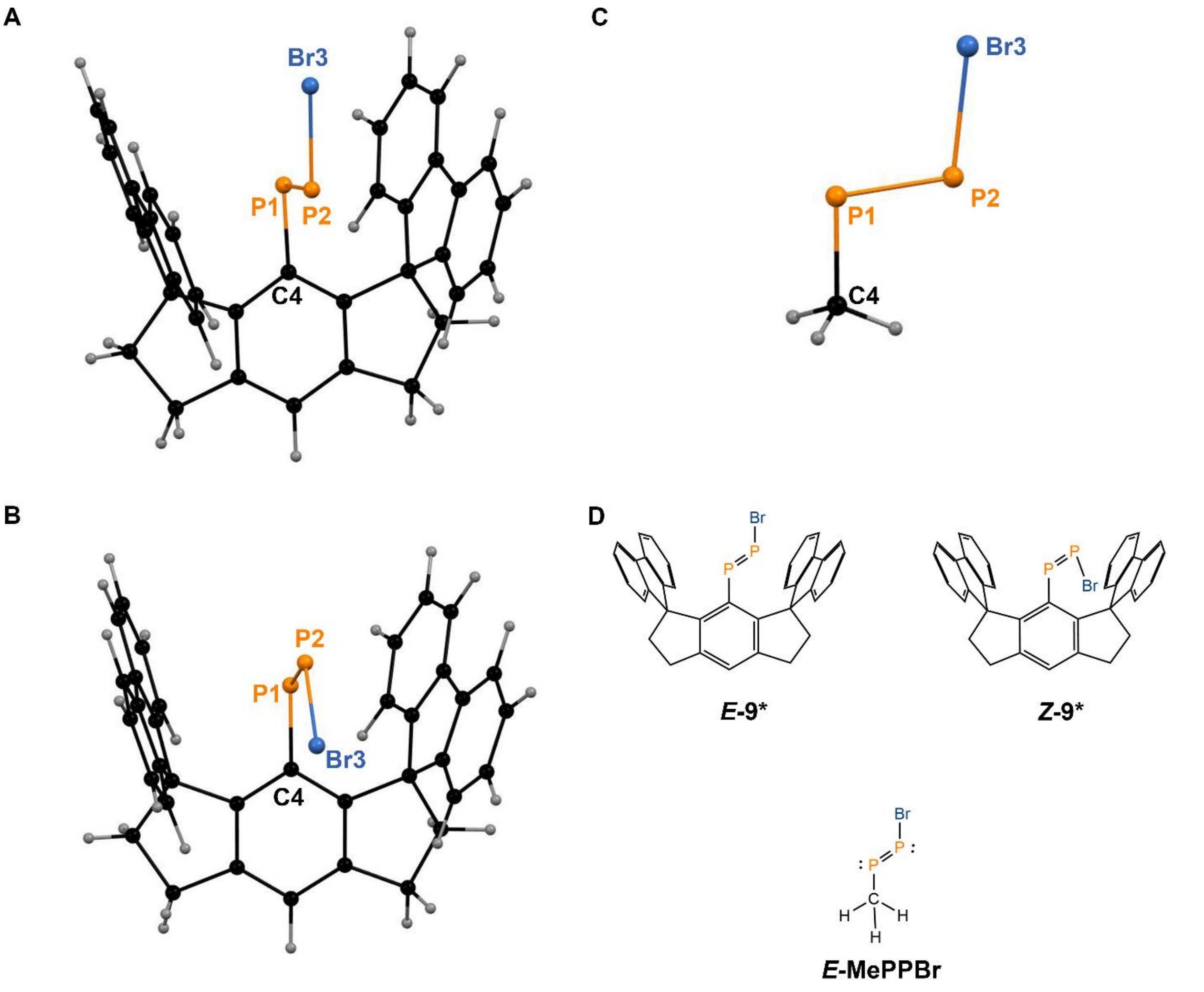

Figure S74. Ball-and-stick representation of geometry-optimized atomic coordinates (PBE0-D3/def2-TZVPP) of (A) ***E*-9***, (B) ***Z*-9***, and (C) ***E*-MePPBr**. (D) Diagrams of ***E*-9***, ***Z*-9***, and ***E*-MePPBr**. Color code: P orange, C black, Br blue, H grey.

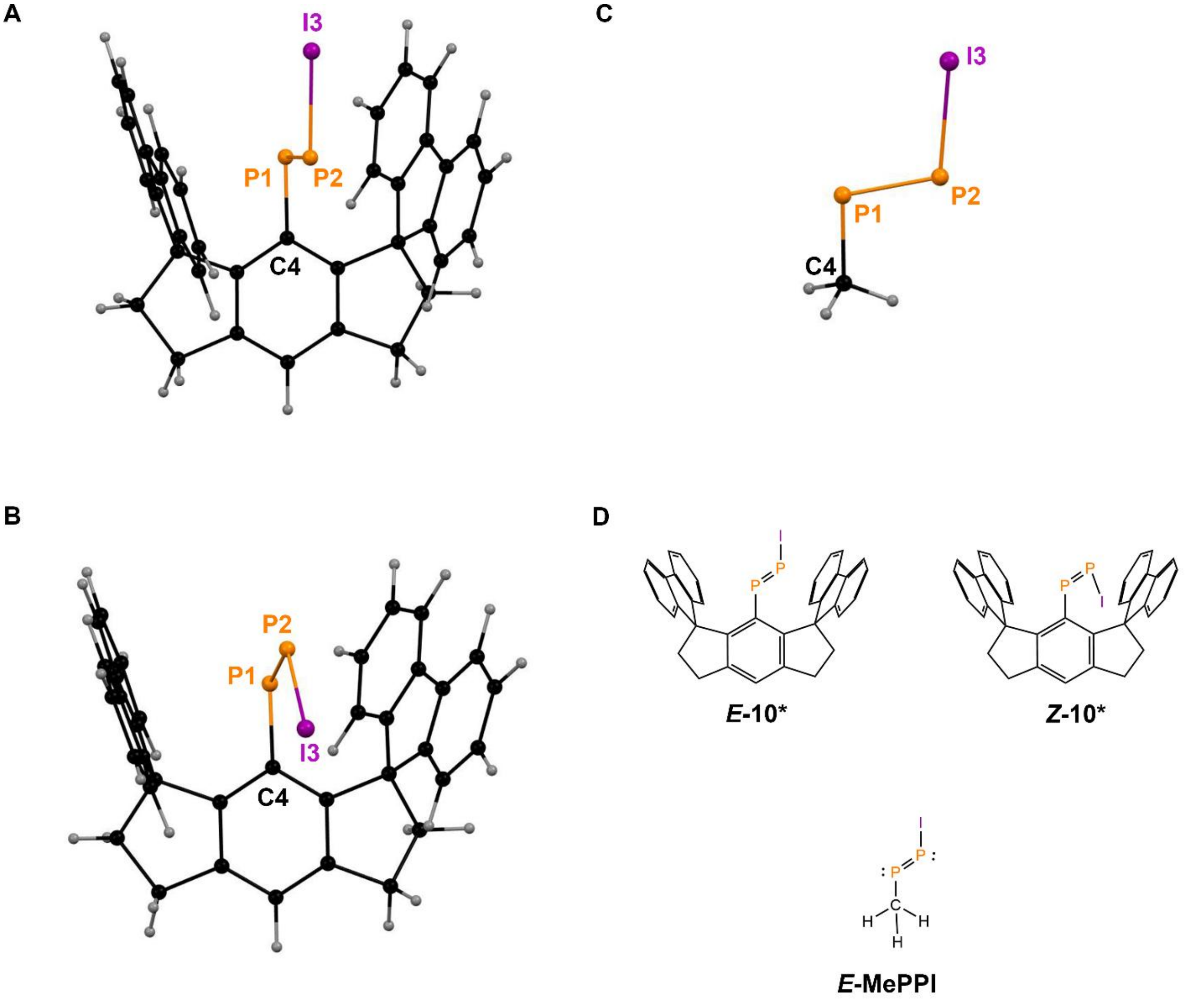

Figure S75. Ball-and-stick representation of geometry-optimized atomic coordinates (PBE0-D3/def2-TZVPP) of (A) ***E*-10***, (B) ***Z*-10***, and (C) ***E*-MePPI**. (D) Diagrams of ***E*-10***, ***Z*-10***, and ***E*-MePPI**. Color code: P orange, C black, I purple, H grey.

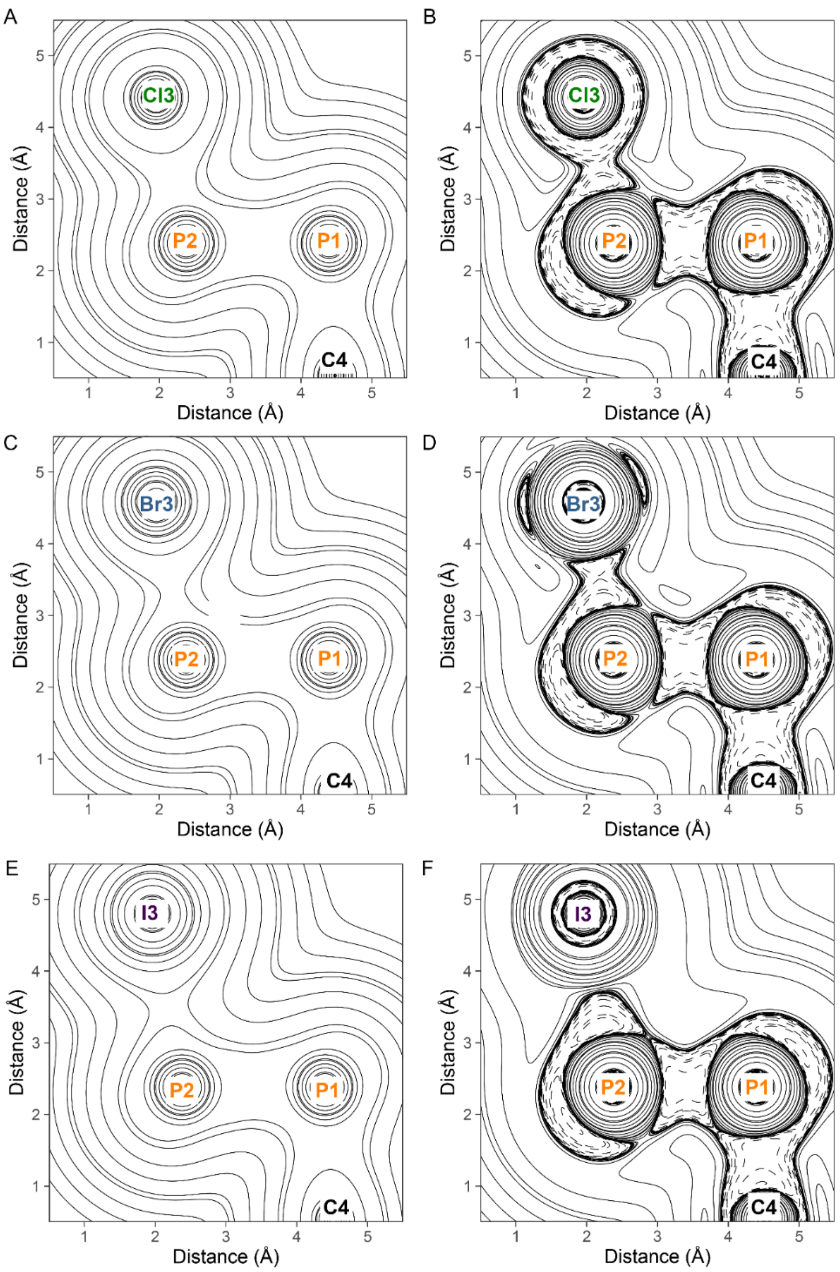

Figure S76. Two-dimensional plot of the (A) ρ and (B) $\nabla^2\rho$ of ***E*-8*** in the P1–P2–Cl3 plane. Two-dimensional plot of the (C) ρ and (D) $\nabla^2\rho$ of ***E*-9*** in the P1–P2–Br3 plane. Two-dimensional plot of the (E) ρ and (F) $\nabla^2\rho$ of ***E*-10*** in the P1–P2–Br3 plane. Positive contour lines (displayed as solid lines) set at 0.001, 0.002, 0.004, 0.008, 0.01, 0.02, 0.04, 0.08, 0.1, 0.2, 0.4, 0.8, 1, 2, 4, 8, 10, 20, 40, 80, 100, 200, 400, 800, 1000, 2000, 4000, 8000, 10000, 20000, 40000, 80000. Negative contour lines (displayed as dashed lines) set at -0.001, -0.002, -0.004, -0.008, -0.01, -0.02, -0.04, -0.08, -0.1, -0.2, -0.4, -0.8, -1, -2, -4, -8, -10, -20, -40, -80, -100, -200, -400, -800, -1000, -2000, -4000, -8000, -10000, -20000, -40000, -80000. Calculations performed at the DKH-PBE0/old-DKH-def2-TZVPP//PBE0-D3/def2-TZVPP level of theory.

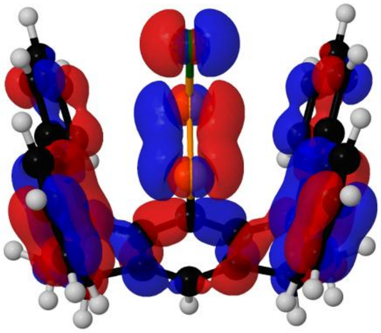

HOMO

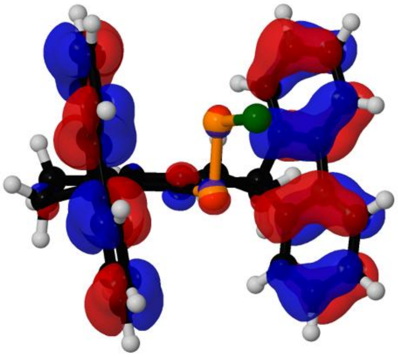

HOMO–1

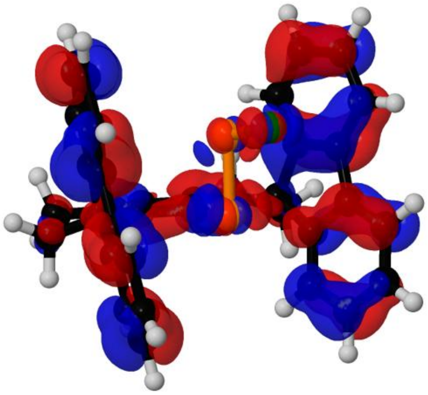

HOMO–2

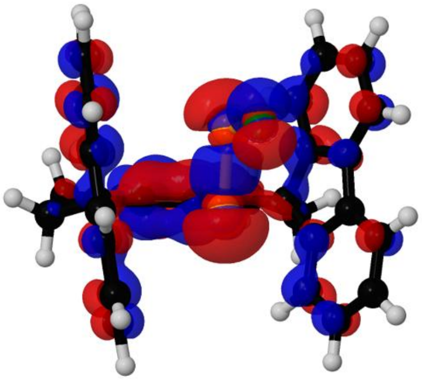

HOMO–3

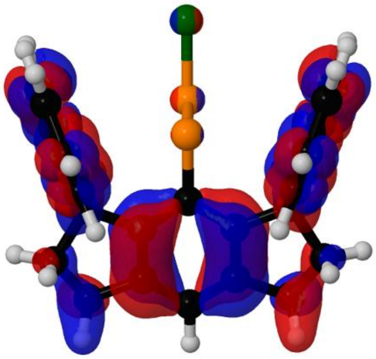

HOMO–4

Figure S77. Canonical molecular orbital diagrams of ***E*-8*** depicting the HOMO, HOMO–1, HOMO–2, HOMO–3, and HOMO–4 (isovalue = 0.015).

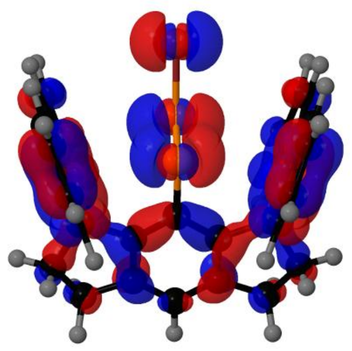

HOMO

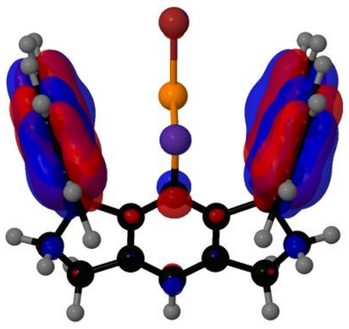

HOMO–1

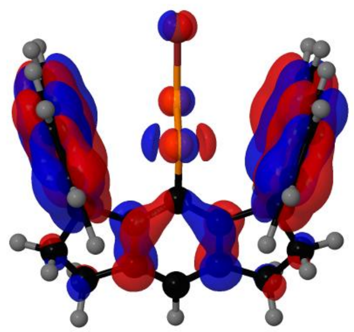

HOMO–2

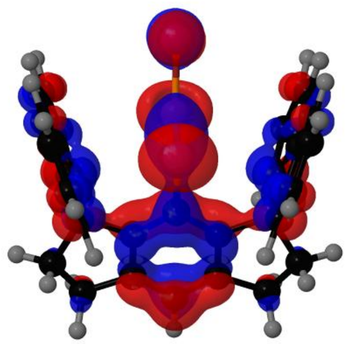

HOMO–3

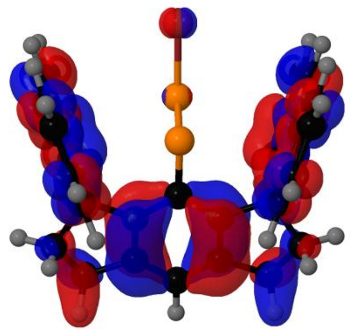

HOMO–4

Figure S78. Canonical molecular orbital diagrams of ***E*-9*** depicting the HOMO, HOMO–1, HOMO–2, HOMO–3, and HOMO–4 (isovalue = 0.015).

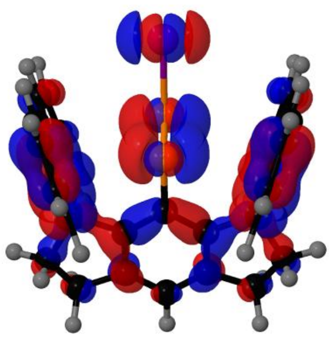

HOMO

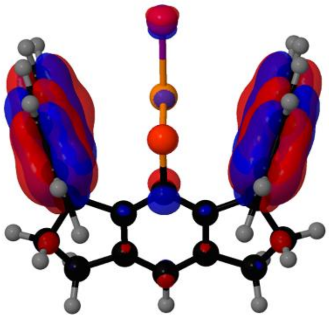

HOMO–1

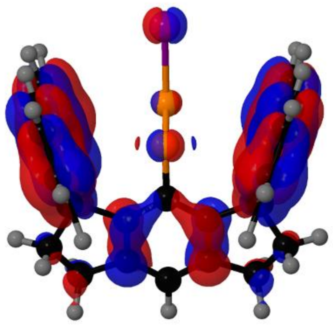

HOMO–2

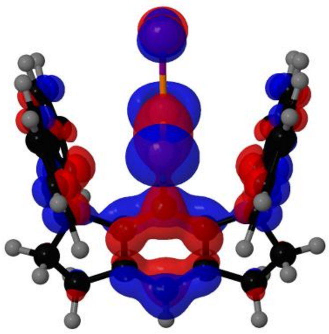

HOMO–3

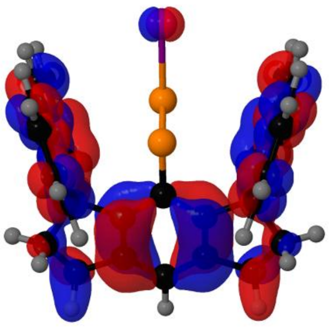

HOMO–4

Figure S79. Canonical molecular orbital diagrams of ***E*-10*** depicting the HOMO, HOMO–1, HOMO–2, HOMO–3, and HOMO–4 (isovalue = 0.015).

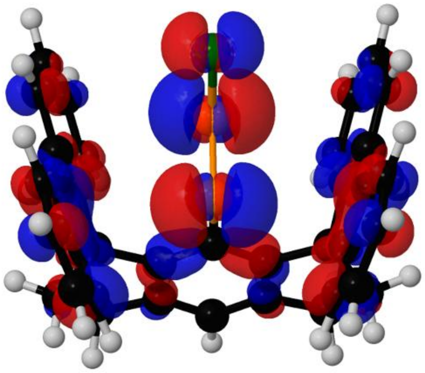

LUMO

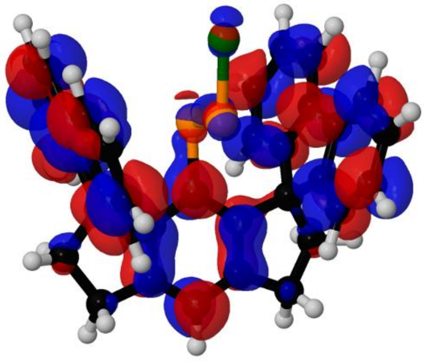

LUMO+1

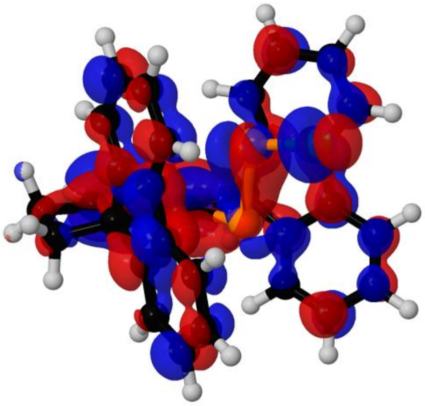

LUMO+2

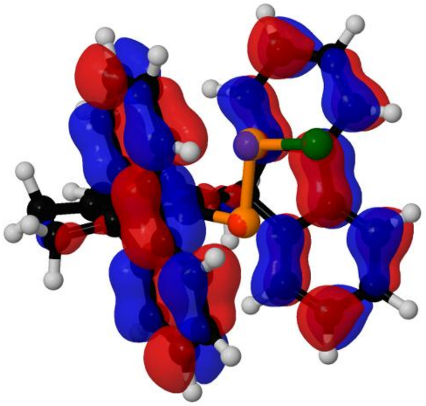

LUMO+3

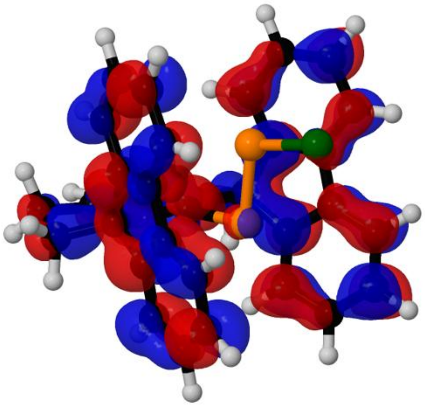

LUMO+4

Figure S80. Canonical molecular orbital diagrams of ***E*-8*** depicting the LUMO, LUMO–1, LUMO–2, LUMO–3, and LUMO–4 (isovalue = 0.015).

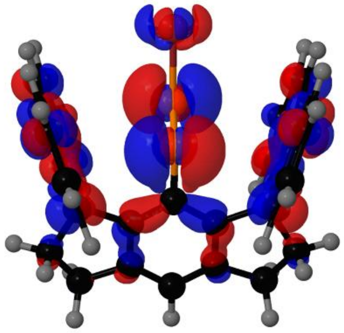

LUMO

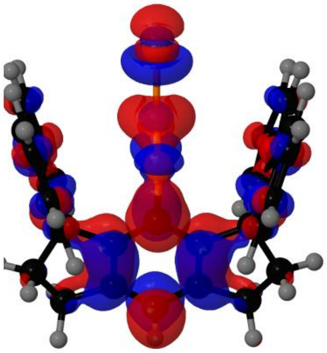

LUMO+1

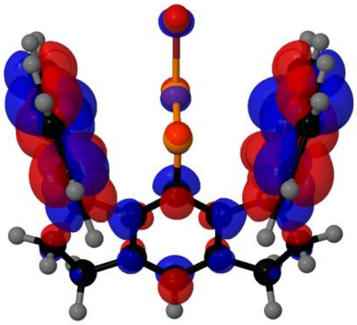

LUMO+2

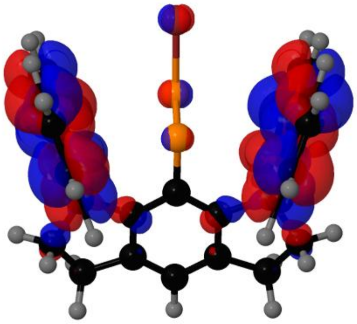

LUMO+3

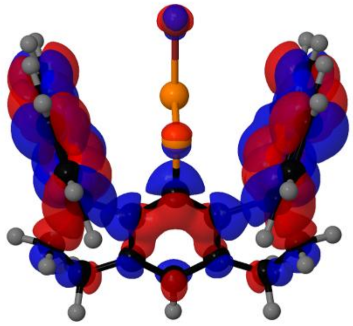

LUMO+4

Figure S81. Canonical molecular orbital diagrams of ***E*-9*** depicting the LUMO, LUMO–1, LUMO–2, LUMO–3, and LUMO–4 (isovalue = 0.015).

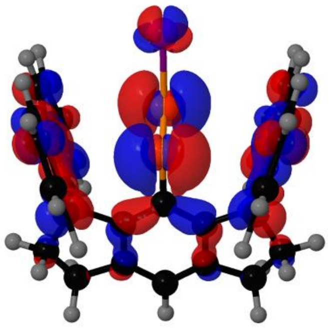

LUMO

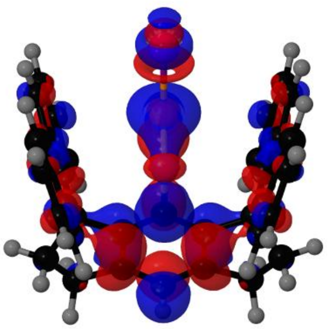

LUMO+1

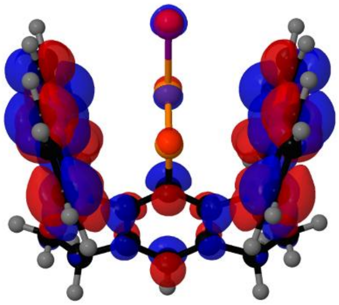

LUMO+2

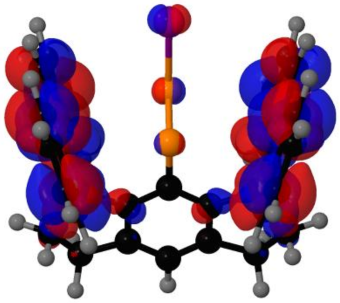

LUMO+3

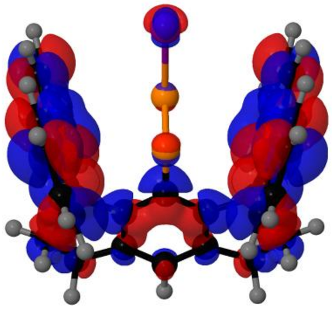

LUMO+4

Figure S82. Canonical molecular orbital diagrams of ***E*-10*** depicting the LUMO, LUMO–1, LUMO–2, LUMO–3, and LUMO–4 (isovalue = 0.015).

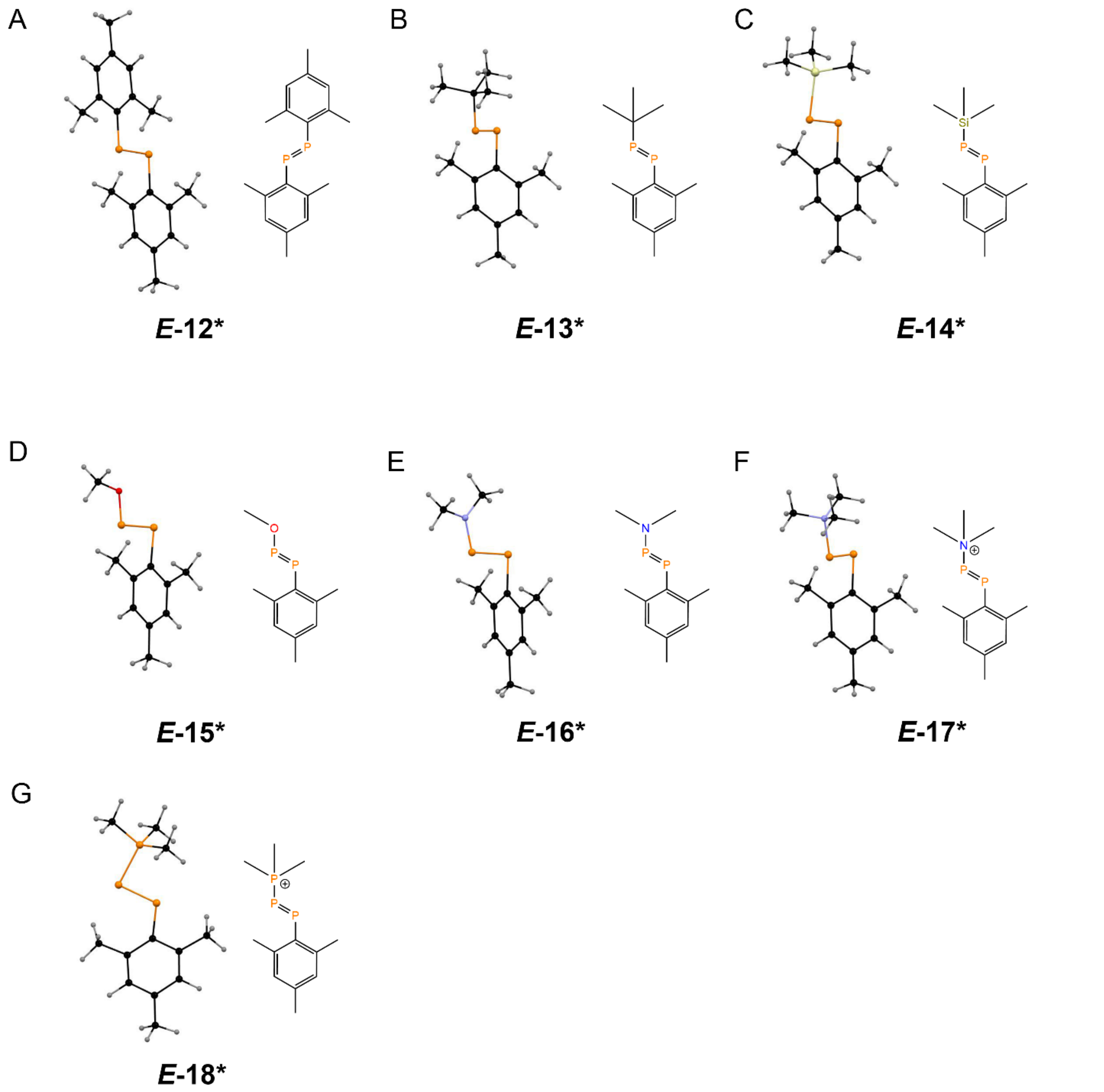

Figure S83. Ball-and-stick representation of geometry-optimized atomic coordinates (PBE0-D3/def2-TZVPP) and diagrams of (A) ***E*-12***, (B) ***E*-13***, (C) ***E*-14***, (D) ***E*-15***, (E) ***E*-16***, (F) ***E*-17***, and (G) ***E*-18***. Color code: P orange, C black, Si dark yellow, N blue, O red, H grey.

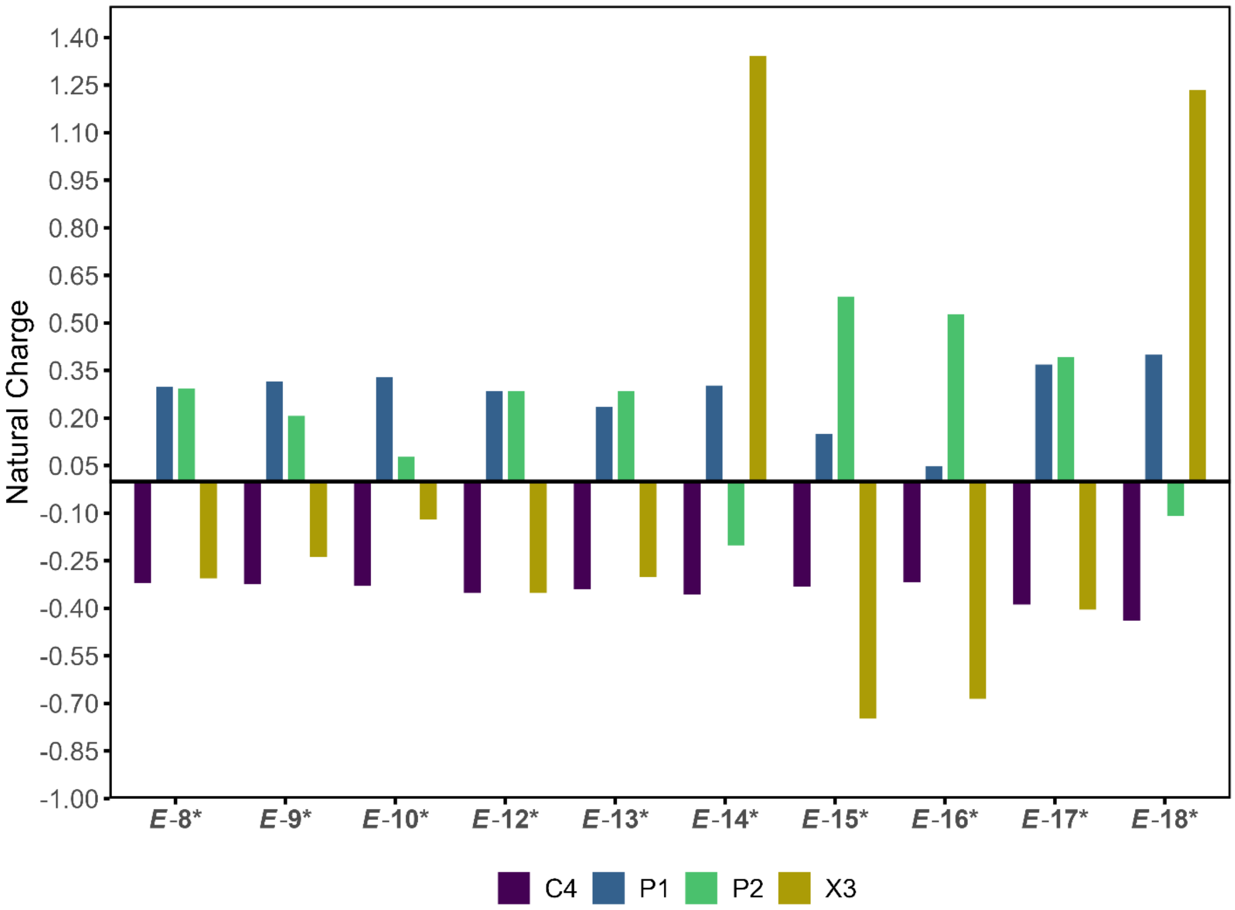

Figure S84. Natural Population Analysis (e⁻), calculated at the DKH-PBE0/old-DKH-TZVPP//PBE0-D3/def2-TZVPP level of theory. P1 refers to the C-bound P atom. P2 refers to the X-bound C-atom. C4 refers to the C atom that is bound to P1. Data are tabulated in Supplementary Table S10.

Table S5. Select bond lengths (Å).[a]

**Bond**	***E*-8***	***E*-9***	***E*-10***
P–P	2.0165	2.0161	2.0177
P–X	2.0767	2.2464	2.4553
P–C	1.8447	1.8436	1.8422

[a] From theoretical coordinates (PBE0-D3/def2-TZVPP).

Table S6. Select bond angles (°).[a]

**Angle**	***E*-8***	***E*-9***	***E*-10***

C–P–P	92.59	92.88	93.47
P–P–X	101.85	100.83	99.87

[a] From theoretical coordinates (PBE0-D3/def2-TZVPP).

Table S7. Approximate stretching force constants (mdyne/Å).[a]

Bond	***E*-8***	***E*-9***	***E*-10***
P–P	3.6969	3.6803	3.6286
P–X	2.1014	1.7316	1.4465
P–C	2.0545	2.0635	2.0751

[a] Calculated at the PBE0-D3/def2-TZVPP level of theory. Force constants were obtained by diagonalization of the Hessian matrix.

Table S8. Stretching Frequencies (cm^{-1}). Intensities (km/mol) are shown in parentheses.[a]

Bond	***E*-8***	***E*-9***	***E*-10***
P–P	656 (0.001)	653 (0.000)	649 (0.000)
P–X	481 (0.012)	392 (0.010)	357 (0.006)
P–C	882 (0.000)	882 (0.000)	882 (0.000)

[a] Calculated at the PBE0-D3/def2-TZVPP level of theory.

Table S9. Enthalpy of formation.[a]

Compound	ΔH_f (Eh)	Relative ΔH_f (Eh)	Relative ΔH_f (kcal/mol)
***E*-8**	-2527.91734085	-0.00471371	-2.957853025
***Z*-8**	-2527.91262714	0	0
***E*-9**	-4641.71629942	-0.005553219999	-3.48464555
***Z*-9**	-4641.71074620	0	0
***E*-10**	-2365.58118604	-0.00610824	-3.8329206
***Z*-10**	-2365.57507780	0	0

[a] Calculated at the PBE0-D3/def2-TZVPP level of theory.

Table S10. Natural Population Analysis (e^-).[a]

Compound	**P1** [b]	**P2** [c]	**X3**	**C4** [d]
E*-8 (X3 = Cl)	0.30264	0.29626	-0.30931	-0.32369
E*-9 (X3 = Br)	0.31863	0.21029	-0.24217	-0.32800

E*-10 (X3 = I)	0.33261	0.08126	-0.12404	-0.33327
E*-12 (X3 = C_{aryl})	0.28826	0.28826	-0.35561	-0.35561
E*-13 (X3 = C_{alkyl})	0.23939	0.28877	-0.30409	-0.34408
E*-14 (X3 = Si)	0.30629	-0.20508	1.34590	-0.36066
E*-15 (X3 = O)	0.15322	0.58703	-0.75202	-0.33542
E*-16 (X3 = N)	0.05103	0.53136	-0.68919	-0.32191
E*-17 (X3 = N^+)	0.37239	0.39601	-0.40795	-0.39169
E*-18 (X3 = P^+)	0.40320	-0.11205	1.23759	-0.44235

[a] Calculated at the DKH-PBE0/old-DKH-TZVPP//PBE0-D3/def2-TZVPP level of theory. [b] P1 refers to the C-bound P atom. [c] P2 refers to the X-bound C-atom. [d] C4 refers to the C atom that is bound to P1.

Table S11. Canonical molecular orbital energies (eV).[a]

Orbital	***E*-8***	***E*-9***	***E*-10***	***E*-12***
LUMO+4	-0.6468	-0.6559	-0.6602	-0.0028
LUMO+3	-0.9869	-0.9902	-0.9931	-0.0277
LUMO+2	-1.0147	-1.0342	-1.0388	-0.2446
LUMO+1	-1.0320	-1.1587	-1.3177	-0.5340
LUMO	-1.9364	-1.9753	-2.0151	-2.3534
HOMO	-6.1958	-6.1597	-6.0753	-6.1474
HOMO–1	-6.2600	-6.2658	-6.268	-6.8006
HOMO–2	-6.3222	-6.3267	-6.3299	-6.8400
HOMO–3	-6.4195	-6.4194	-6.3691	-6.8555
HOMO–4	-6.6880	-6.6932	-6.6894	-7.0320

[a] Calculated at the DKH-PBE0/old-DKH-TZVPP//PBE0-D3/def2-TZVPP level of theory.

Table S12. Select results from TD-DFT calculation of ***E*-8***.[a]

State	Energy (eV)	Wavelength (nm)	Excitation	Weight of excitation
1	3.268	379.4	HOMO–3 to LUMO	0.94
			HOMO–1 to LUMO	0.03
2	3.466	357.8	HOMO–3 to LUMO	0.03
			HOMO–1 to LUMO	0.96
3	3.536	350.7	HOMO–2 to LUMO	0.73
			HOMO to LUMO	0.26

[a] Calculated at the DKH-PBE0/old-DKH-TZVPP//PBE0-D3/def2-TZVPP level of theory. Simulated UV-Vis spectrum is provided in Supplementary Figure S35.

Table S13. Select results from TD-DFT calculation of ***E*-9***.[a]

State	Energy (eV)	Wavelength (nm)	Excitation	Weight of excitation
1	3.237	383.0	HOMO–3 to LUMO	0.96
2	3.432	361.2	HOMO–1 to LUMO	0.97
3	3.506	353.6	HOMO–2 to LUMO	0.77
			HOMO to LUMO	0.20

[a] Calculated at the DKH-PBE0/old-DKH-TZVPP//PBE0-D3/def2-TZVPP level of theory. Simulated UV-Vis spectrum is provided in Supplementary Figure S45.

Table S14. Select results from TD-DFT calculation of ***E*-10***.[a]

State	Energy (eV)	Wavelength (nm)	Excitation	Weight of excitation
1	3.160	392.4	HOMO–3 to LUMO	0.96
			HOMO–1 to LUMO	0.02
2	3.395	365.2	HOMO–3 to LUMO	0.02
			HOMO–1 to LUMO	0.97
3	3.455	358.8	HOMO–2 to LUMO	0.65
			HOMO to LUMO	0.32
4	3.613	343.2	HOMO–2 to LUMO+1	0.02
			HOMO to LUMO+1	0.93
			HOMO to LUMO+6	0.02

[a] Calculated at the DKH-PBE0/old-DKH-TZVPP//PBE0-D3/def2-TZVPP level of theory. Simulated UV-Vis spectrum is provided in Supplementary Figure S54.

Table S15. Natural Localized Molecular Orbital analysis of ***E*-8*.** [a]

NLMO	% atom 1	% atom 2	%s character 1	%p character 1	%s character 2	%p character 2	WBI
P1–P2 σ	48.650	49.125	16.799	82.05	18.61	80.03	1.8095

P1–P2 π	47.784	49.853	0	98.58	0	98.77	N/A
P1–C4 σ	35.074	62.266	14.94	84.06	27.63	72.21	0.9195
P2–Cl3 σ	29.528	69.811	7.48	90.71	16.68	82.79	0.9018
LP P1	N/A	N/A	70.25	29.63	N/A	N/A	N/A
LP P2	N/A	N/A	76.19	23.74	N/A	N/A	N/A

[a] Calculated at the DKH-PBE0/old-DKH-TZVPP//PBE0-D3/def2-TZVPP level of theory.

Table S16. Natural Localized Molecular Orbital analysis of ***E*-9*.** [a]

NLMO	% atom 1	% atom 2	%s character 1	%p character 1	%s character 2	%p character 2	WBI
P1–P2 σ	48.731	49.043	17.51	81.45	18.25	80.41	1.8088
P1–P2 π	46.750	50.906	0	98.52	0	98.72	N/A
P1–C4 σ	34.861	62.424	14.83	84.13	27.64	72.20	0.9125
P2–Br3 σ	32.989	66.154	24.19	57.35	12.44	87.16	0.9333
LP P1	N/A	N/A	69.90	29.96	N/A	N/A	N/A
LP P2	N/A	N/A	77.28	22.65	N/A	N/A	N/A

[a] Calculated at the DKH-PBE0/old-DKH-TZVPP//PBE0-D3/def2-TZVPP level of theory.

Table S17. Natural Localized Molecular Orbital analysis of ***E*-10*.** [a]

NLMO	% atom 1	% atom 2	%s character 1	%p character 1	%s character 2	%p character 2	WBI
P1–P2 σ	48.765	49.033	17.94	80.96	18.12	80.55	1.8084
P1–P2 π	45.699	52.033	0	98.50	0	98.78	N/A
P1–C4 σ	34.680	62.678	14.80	84.14	27.68	72.16	0.9063
P2–I3 σ	38.651	60.180	5.40	92.91	10.40	89.48	0.9787

LP P1	N/A	N/A	69.52	30.34	N/A	N/A	N/A
LP P2	N/A	N/A	77.64	22.26	N/A	N/A	N/A

[a] Calculated at the DKH-PBE0/old-DKH-TZVPP//PBE0-D3/def2-TZVPP level of theory.

Table S18. Natural Resonance Theory analysis of ***E*-MePPCl*.**[a]

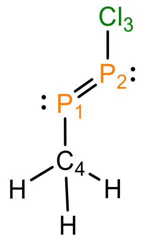

92.88 %

**Bond**	**Total** [b]	**Covalent** [c]	**Ionic** [d]
P1–P2	1.9025	1.8494	0.0531
P2–Cl3	1.0000	0.6211	0.3789
P1–Cl3	0.0350	0.0043	0.0307
P1–C4	0.9901	0.7580	0.2321
LP P1 [e]	1.0362	N/A	N/A
LP P2 [e]	1.0263	N/A	N/A
LP Cl3 [e]	2.9551	N/A	N/A
LP C4 [e]	0.0449	N/A	N/A

[a] Performed at the (DKH-PBE0/old-DKH-TZVPP//PBE0-D3/def2-TZVPP) level of theory. Diagrams show the three resonance structures found during the NRT calculation. The percentages show the relative weights of the resonance structures. [b] Total Natural bond orders found in the major resonance structure. [c] Covalent contribution to bond order. [d] Ionic contribution to bond order. [e] Lone pair character of atomic center.

Table S19. Natural Resonance Theory analysis of ***E*-MePPBr*.**[a]

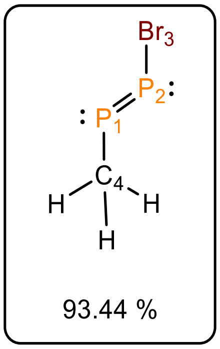

Bond	Total [b]	Covalent [c]	Ionic [d]
P1–P2	1.9344	1.8634	0.0710
P2–Br3	1.0000	0.6944	0.3056
P1–Br3	0.0379	0.0046	0.0332
P1–C4	0.9723	0.7430	0.2293
LP P1 [e]	1.0277	N/A	N/A
LP P2 [e]	1.0000	N/A	N/A
LP Br3 [e]	2.9344	N/A	N/A
LP C4 [e]	0.0656	N/A	N/A

[a] Performed at the (DKH-PBE0/old-DKH-TZVPP//PBE0-D3/def2-TZVPP) level of theory. Diagrams show the three resonance structures found during the NRT calculation. The percentages show the relative weights of the resonance structures. [b] Total Natural bond orders found in the major resonance structure. [c] Covalent contribution to bond order. [d] Ionic contribution to bond order. [e] Lone pair character of atomic center.

Table S20. Natural Resonance Theory analysis of ***E*-MePPI*.**[a]

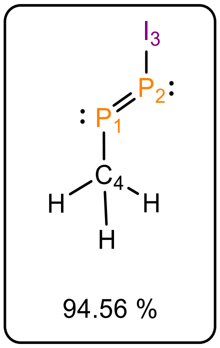

Bond	Total [b]	Covalent [c]	Ionic [d]
P1–P2	1.9400	1.8502	0.0899
P2–I3	1.0000	0.8170	0.1830
P1–I3	0.0252	0.0031	0.0220
P1–C4	0.9764	0.7447	0.2317
LP P1 [e]	1.0292	N/A	N/A

LP P2 [e]	1.0056	N/A	N/A
LP I3 [e]	2.9512	N/A	N/A
LP C4 [e]	0.0488	N/A	N/A

[a] Performed at the (DKH-PBE0/old-DKH-TZVPP//PBE0-D3/def2-TZVPP) level of theory. Diagrams show the three resonance structures found during the NRT calculation. The percentages show the relative weights of the resonance structures. [b] Total Natural bond orders found in the major resonance structure. [c] Covalent contribution to bond order. [d] Ionic contribution to bond order. [e] Lone pair character of atomic center.

Table S21. Calculated ^{31}P NMR data. [a]

**Compound**	**δ P1 [b] (ppm)**	**δ P2 [c] (ppm)**	J_{PP} **(Hz)**
E*-8	495.4	534.3	509.0
**Z-8***	425.9	473.6	330.6

[a] Performed at the (PBE0-D4/pcsseg-2//PBE0-D3/def2-TZVPP) level of theory. [b] P1 refers to the C-bound P atom. [c] P2 refers to the X-bound C-atom.

Table S22. Geometry optimized coordinates for ***E*-8*** (PBE0-D3/def2-TZVPP).

**Atom**	**x**	**y**	**z**
P	17.15015	8.322091	4.030578
P	18.50024	9.674395	3.386582
Cl	18.32559	9.502376	1.324363
C	17.51081	8.682244	5.803513
C	16.78585	9.638864	6.518736
C	13.10793	8.452431	4.159249
C	15.71918	10.58042	5.988796
C	14.73689	10.66206	3.802122
C	14.74323	9.963641	5.015876
C	13.9277	8.862817	5.202205
H	13.94383	8.315504	6.137996
C	13.10812	9.138671	2.948909
C	13.92006	10.24777	2.760118
H	13.92057	10.77115	1.811609
C	17.01331	9.867369	7.874095
C	15.04934	11.07481	7.297339
C	16.11698	10.95647	8.388458
C	17.97265	9.144446	8.565742
H	18.14492	9.316844	9.622848
C	18.46771	7.957869	6.518822

C	22.35136	8.900149	4.536804
C	19.40978	6.891736	5.988828
C	20.56861	6.889654	3.886447
C	20.52984	7.486307	5.153167
C	21.41677	8.492882	5.481972
H	21.37188	8.980921	6.448313
C	22.38471	8.315612	3.276161
C	21.49129	7.308499	2.939328
H	21.50773	6.869115	1.949185
C	18.69603	8.185419	7.874204
C	19.90428	6.221949	7.29738
C	19.7855	7.2895	8.388542
C	17.72628	13.52245	4.535914
C	15.71647	11.73871	3.886104
C	16.31315	11.70058	5.152844
C	17.31935	12.58803	5.481392
H	17.80741	12.5436	6.447739
C	17.14178	13.55512	3.27523
C	16.13503	12.66119	2.938665
H	15.69572	12.6771	1.948484
C	17.28338	4.279675	4.158608
C	19.49236	5.909651	3.802123
C	18.79361	5.915652	5.015683
C	17.69304	5.099704	5.201649
H	17.14541	5.115608	6.137256
C	17.97008	4.280027	2.948529
C	19.07886	5.09249	2.760062
H	19.60261	5.093103	1.811759
H	16.67784	11.88989	8.500472
H	15.68976	10.72694	9.366542
H	19.55611	6.862129	9.366596
H	20.71869	7.850733	8.500604
H	23.10765	8.658082	2.546027
H	23.04861	9.693077	4.777952
H	17.48403	14.27789	2.544831
H	18.51894	14.22009	4.77683
H	12.46926	8.798604	2.143016
H	16.41615	3.642938	4.282601
H	17.63065	3.640882	2.142602
H	12.47157	7.584961	4.283485
H	20.90928	5.816329	7.187089
H	19.23286	5.39255	7.52804
H	14.22008	10.40327	7.528095

H	14.6435	12.07973	7.186982

Table S23. Geometry optimized coordinates for ***Z*-8*** (PBE0-D3/def2-TZVPP).

Atom	x	y	z
P	9.246166	17.22948	7.509626
P	8.027929	18.45101	8.562048
Cl	6.877398	19.60045	7.30887
C	8.904299	17.5687	5.745481
C	9.330191	13.20185	7.346753
C	7.952734	16.83483	5.0324
C	7.003012	14.66088	7.69368
C	7.727606	14.75379	6.499572
C	7.063944	15.71168	5.539024
C	8.61504	13.11423	8.536698
C	8.887787	14.02365	6.31808
C	7.446669	13.84021	8.719942
C	7.718216	17.07074	3.678995
C	6.625902	15.03558	4.213016
C	6.666175	16.13776	3.152349
C	8.429538	18.03965	2.989912
C	9.063743	22.55775	6.94514
C	9.637754	18.51872	5.029896
C	10.91717	20.6161	7.600684
C	10.2752	20.58724	6.356544
C	10.76232	19.40749	5.533384
C	9.695261	22.58378	8.182603
C	9.345703	21.55318	6.026268
C	10.62443	21.61035	8.522372
C	9.399927	18.75136	3.676494
C	11.43666	19.84305	4.205658
C	10.33279	19.80196	3.146795
C	13.27383	17.14274	7.340619
C	11.81664	19.47128	7.686214
C	11.72136	18.74482	6.493434
C	13.36379	17.85975	8.529272
C	12.45057	17.58396	6.312595
C	12.63875	19.02881	8.71183
C	3.916953	17.41437	6.953065
C	5.858594	15.5609	7.608519

C	5.885814	16.20079	6.363284
C	3.892555	16.78491	8.191609
C	4.919868	17.13029	6.033039
C	4.866001	15.85572	8.531326
H	12.20244	19.10558	3.956664
H	11.92296	20.81328	4.300938
H	9.820399	20.7659	3.066642
H	10.71575	19.56455	2.152509
H	6.901811	15.75307	2.158313
H	5.702473	16.6507	3.07282
H	5.6555	14.55008	4.310505
H	7.362492	14.26893	3.964095
H	10.2431	12.63165	7.226814
H	8.978023	12.47367	9.331282
H	9.454145	14.10776	5.39729
H	6.899072	13.77023	9.652242
H	8.256842	18.21085	1.932772
H	8.330767	23.3169	6.701438
H	9.452783	23.3662	8.891253
H	8.819254	21.51753	5.080372
H	11.10767	21.62788	9.492056
H	13.84333	16.22933	7.221202
H	14.00546	17.49769	9.323383
H	12.36465	17.0162	5.392835
H	12.71059	19.57788	9.643123
H	3.15783	18.14737	6.709358
H	3.111423	17.029	8.901123
H	4.95424	17.65512	5.086194
H	4.849725	15.37405	9.501816

Table S24. Geometry optimized coordinates for ***E*-9*** (PBE0-D3/def2-TZVPP).

Atom	x	y	z
P	17.11763	8.289904	4.055662
P	18.46169	9.631353	3.378329
Br	18.22018	9.386831	1.15836
C	17.49359	8.665334	5.821064
C	16.77538	9.628252	6.533562
C	13.10103	8.467209	4.158133
C	15.71937	10.57829	5.99825
C	14.75992	10.65387	3.800815
C	14.74303	9.967427	5.021631

C	13.91148	8.878485	5.208022
H	13.90829	8.340224	6.149171
C	13.12656	9.139823	2.940472
C	13.95361	10.23758	2.751466
H	13.97496	10.7498	1.797174
C	17.00746	9.861564	7.887015
C	15.05332	11.08529	7.303463
C	16.11751	10.95782	8.397851
C	17.96757	9.138453	8.578071
H	18.14373	9.31432	9.633967
C	18.45598	7.946096	6.5333
C	22.31052	8.945712	4.539306
C	19.40509	6.889316	5.997741
C	20.54581	6.920858	3.885098
C	20.51322	7.502518	5.158981
C	21.39132	8.516217	5.489781
H	21.35117	8.992829	6.462014
C	22.33675	8.376777	3.27141
C	21.45216	7.362673	2.93253
H	21.46113	6.936866	1.93644
C	18.68966	8.177753	7.886766
C	19.91151	6.222561	7.302791
C	19.78508	7.286648	8.397401
C	17.7778	13.48212	4.539533
C	15.7513	11.71916	3.885704
C	16.33331	11.68594	5.159408
C	17.34787	12.56312	5.490001
H	17.82476	12.52242	6.462075
C	17.20845	13.50904	3.271837
C	16.19352	12.62532	2.933146
H	15.76737	12.63482	1.937209
C	17.29156	4.273359	4.157066
C	19.47969	5.930393	3.800032
C	18.79333	5.913761	5.020907
C	17.7037	5.08307	5.207184
H	17.16555	5.080066	6.148394
C	17.96402	4.298745	2.939311
C	19.06252	5.124844	2.750448
H	19.5746	5.146094	1.796078
H	16.68512	11.88683	8.512116
H	15.68532	10.73148	9.374516
H	19.55829	6.854447	9.373959
H	20.71464	7.85333	8.511831

H	23.04646	8.737455	2.537094
H	23.00076	9.744262	4.78211
H	17.56944	14.2186	2.537531
H	18.57698	14.1717	4.78218
H	12.496	8.797819	2.128913
H	16.43239	3.625883	4.281376
H	17.62131	3.668805	2.127567
H	12.45281	7.60861	4.282569
H	20.9209	5.82928	7.188006
H	19.25124	5.384451	7.534173
H	14.21463	10.42582	7.534988
H	14.66099	12.09507	7.188792

Table S25. Geometry optimized coordinates for ***Z*-9*** (PBE0-D3/def2-TZVPP).

Atom	x	y	z
P	9.278976	17.19502	7.501298
P	8.114782	18.36046	8.66631
Br	6.801346	19.67452	7.453334
C	8.89832	17.57568	5.753024
C	9.328282	13.20307	7.34011
C	7.944584	16.84433	5.038328
C	7.001951	14.66364	7.69195
C	7.725675	14.75722	6.497457
C	7.060412	15.7129	5.536903
C	8.615129	13.11601	8.531275
C	8.885233	14.02685	6.313555
C	7.447958	13.84345	8.717385
C	7.711151	17.08249	3.684995
C	6.632883	15.03779	4.207437
C	6.66833	16.14349	3.150972
C	8.422834	18.05136	2.997185
C	9.105306	22.5927	6.929693
C	9.630139	18.52906	5.038313
C	10.91978	20.62098	7.603812
C	10.28547	20.59931	6.355696
C	10.76209	19.41255	5.536816
C	9.726438	22.60983	8.172538
C	9.375495	21.58035	6.015915
C	10.63595	21.62151	8.521738

C	9.392034	18.76259	3.684997
C	11.43739	19.83964	4.207284
C	10.33154	19.80495	3.150945
C	13.27076	17.14319	7.339774
C	11.81172	19.47047	7.691698
C	11.71747	18.74672	6.497273
C	13.3585	17.85639	8.530856
C	12.44707	17.58668	6.313332
C	12.63183	19.02403	8.717013
C	3.881303	17.37179	6.929645
C	5.852004	15.55631	7.603997
C	5.873985	16.19037	6.355767
C	3.863862	16.75088	8.172601
C	4.893448	17.10084	6.015862
C	4.851675	15.84086	8.521917
H	12.19687	19.09588	3.957898
H	11.93128	20.80624	4.300093
H	9.827092	20.77279	3.068406
H	10.71052	19.56047	2.156819
H	6.912733	15.76423	2.156934
H	5.700789	16.64847	3.068214
H	5.665926	14.54459	4.300248
H	7.376094	14.27774	3.958141
H	10.2401	12.6317	7.217993
H	8.97857	12.47429	9.324699
H	9.44987	14.11201	5.391839
H	6.901808	13.77344	9.650505
H	8.254726	18.21957	1.938856
H	8.388631	23.36473	6.678033
H	9.491779	23.398	8.87737
H	8.85673	21.55284	5.06557
H	11.11308	21.63377	9.494484
H	13.84152	16.23099	7.21763
H	14.00015	17.49262	9.324183
H	12.36138	17.02202	5.391675
H	12.70237	19.57022	9.650069
H	3.109661	18.08885	6.677889
H	3.075854	16.98611	8.87742
H	4.921199	17.61943	5.065423
H	4.839175	15.3639	9.494739

Table S26. Geometry optimized coordinates for ***E*-10*** (PBE0-D3/def2-TZVPP).

Atom	x	y	z
P	17.11465	8.287076	4.068938
P	18.45378	9.624263	3.369108
I	18.13951	9.310219	0.954346
C	17.49205	8.663713	5.832339
C	16.77533	9.627558	6.544111
C	13.11924	8.45535	4.156552
C	15.72099	10.57838	6.006715
C	14.78078	10.63993	3.800096
C	14.75059	9.963415	5.02649
C	13.91682	8.875982	5.212537
H	13.90275	8.345503	6.157985
C	13.15996	9.116496	2.933031
C	13.98838	10.21319	2.74423
H	14.02239	10.71603	1.78537
C	17.00685	9.860443	7.897518
C	15.0539	11.08636	7.310515
C	16.11578	10.9564	8.407504
C	17.96658	9.136291	8.588523
H	18.14197	9.310969	9.644747
C	18.45546	7.945494	6.543277
C	22.30225	8.956387	4.544231
C	19.40463	6.8903	6.004887
C	20.53144	6.938834	3.884745
C	20.50904	7.508287	5.164432
C	21.39029	8.518321	5.497877
H	21.35824	8.985634	6.474907
C	22.31804	8.399856	3.270707
C	21.43018	7.389541	2.929063
H	21.43055	6.973889	1.928673
C	18.6894	8.17615	7.896642
C	19.91275	6.222107	7.30812
C	19.78469	7.283671	8.405612
C	17.78977	13.47484	4.547577
C	15.77036	11.7065	3.887265
C	16.34002	11.68263	5.166821
C	17.35094	12.5627	5.500694
H	17.81831	12.52968	6.477668
C	17.23307	13.49202	3.274145
C	16.22178	12.60542	2.932106
H	15.80595	12.60686	1.931793
C	17.27785	4.290893	4.155766
C	19.46387	5.950308	3.798123

C	18.78804	5.920722	5.024893
C	17.69986	5.08804	5.211525
H	17.16989	5.074537	6.157269
C	17.93834	4.331011	2.931875
C	19.03572	5.158387	2.742469
H	19.53804	5.191953	1.783316
H	16.68331	11.88507	8.52483
H	15.68093	10.72876	9.382694
H	19.55718	6.848677	9.380765
H	20.71405	7.85015	8.522582
H	23.0218	8.767478	2.53411
H	22.99504	9.752047	4.789303
H	17.60126	14.1959	2.537957
H	18.58613	14.1667	4.79301
H	12.54047	8.766041	2.116582
H	16.41958	3.642277	4.280381
H	17.58679	3.711931	2.115589
H	12.46976	7.597672	4.280724
H	20.92304	5.831382	7.192409
H	19.2546	5.38192	7.538001
H	14.21303	10.42906	7.540362
H	14.6643	12.09718	7.195661

Table S27. Geometry optimized coordinates for ***Z*-10*** (PBE0-D3/def2-TZVPP).

Atom	x	y	z
P	9.2244	17.2505	7.521867
P	8.063793	18.41224	8.694506
I	6.607501	19.86761	7.407409
C	8.858754	17.61425	5.767316
C	9.393289	13.2794	7.348913
C	7.913228	16.87334	5.050726
C	7.025494	14.67198	7.699878
C	7.745129	14.78409	6.504511
C	7.052753	15.71994	5.543287
C	8.684949	13.17557	8.541726
C	8.9252	14.08695	6.320804
C	7.497327	13.86889	8.72751
C	7.686645	17.10365	3.694977
C	6.650107	15.03726	4.209975

C	6.662551	16.14663	3.156675
C	8.398645	18.0714	3.005915
C	9.231011	22.67422	6.910915
C	9.599423	18.55846	5.048738
C	10.95497	20.62854	7.601432
C	10.32068	20.62513	6.352954
C	10.75402	19.41872	5.538859
C	9.849859	22.67189	8.154857
C	9.456214	21.64376	6.004942
C	10.71416	21.64661	8.512239
C	9.367473	18.78377	3.693031
C	11.43511	19.81955	4.204156
C	10.32418	19.80699	3.152538
C	13.19657	17.07897	7.342785
C	11.80602	19.44802	7.693458
C	11.69105	18.72684	6.49929
C	13.30331	17.7889	8.5344
C	12.38718	17.54615	6.315711
C	12.61104	18.97716	8.720022
C	3.799742	17.24725	6.916413
C	5.845413	15.52362	7.607769
C	5.847499	16.15534	6.357992
C	3.803477	16.63108	8.161681
C	4.828834	17.01957	6.009504
C	4.828752	15.76695	8.519484
H	12.17355	19.05461	3.955551
H	11.95452	20.77339	4.28928
H	9.839167	20.78477	3.071386
H	10.69344	19.55352	2.157066
H	6.915313	15.77571	2.161637
H	5.684957	16.63196	3.075292
H	5.69586	14.51887	4.296783
H	7.414089	14.2977	3.961753
H	10.32082	12.73388	7.226858
H	9.068386	12.54746	9.336609
H	9.485991	14.18579	5.398076
H	6.954733	13.78577	9.661596
H	8.23753	18.23134	1.945239
H	8.550727	23.47619	6.652144
H	9.650365	23.47501	8.853556
H	8.939968	21.63465	5.052978
H	11.19172	21.64556	9.484835
H	13.74135	16.151	7.220828

H	13.93293	17.40624	9.328467
H	12.28624	16.98425	5.393884
H	12.69651	19.52102	9.653158
H	2.997792	17.92745	6.657332
H	3.001465	16.83256	8.861085
H	4.83697	17.53385	5.056468
H	4.830869	15.29148	9.493099

Table S28. Geometry optimized coordinates for ***E*-MePPCl** (PBE0-D3/def2-TZVPP).

Atom	x	y	z
P	17.73997	8.083617	4.067172
P	18.02402	9.913313	3.275706
Cl	18.23382	9.527383	1.267835
C	17.58495	8.661332	5.821922
H	16.60957	8.35398	6.203151
H	17.68989	9.740505	5.948482
H	18.34989	8.160317	6.417915

Table S29. Geometry optimized coordinates for ***E*-MePPBr** (PBE0-D3/def2-TZVPP).

Atom	x	y	z
P	16.92584	8.918335	4.084692
P	18.76112	9.192667	3.302215
Br	18.34541	9.428852	1.13061
C	17.48549	8.753074	5.845055
H	17.16779	7.777914	6.218918
H	16.98253	9.518696	6.438927
H	18.56393	8.850909	5.981766

Table S30. Geometry optimized coordinates for ***E*-MePPI** (PBE0-D3/def2-TZVPP).

Atom	x	y	z
P	17.74543	8.130849	4.104884
P	18.0189	9.971632	3.330331
I	18.2737	9.507326	0.95434
C	17.57704	8.650627	5.876119
H	16.60009	8.325384	6.239587
H	17.67778	9.724955	6.037511
H	18.33918	8.129675	6.459411

Table S31. Geometry optimized coordinates for ***E*-12*** (PBE0-D3/def2-TZVPP).

Atom	x	y	z
P	0.217672	1.605449	5.205894
C	0.328304	1.583067	7.034753
C	0.474996	2.786692	7.74135
C	0.62992	2.745296	9.119439
C	0.646849	1.544531	9.820089
C	0.511418	0.366351	9.100702
C	0.354125	0.361317	7.719178
C	0.449148	4.106427	7.031911
C	0.815016	1.530423	11.30848
C	0.203675	-0.94235	6.995219
H	0.74213	3.679366	9.661335
H	0.530677	-0.58229	9.627732
P	-1.78549	1.604329	4.980171
C	-1.89609	1.582322	3.151306
C	-2.04268	2.786092	2.444949
C	-1.92201	0.360703	2.466634
C	-2.19758	2.74499	1.066849
C	-2.0167	4.105674	3.154667
C	-2.0793	0.366031	1.085111
C	-1.77173	-0.94313	3.19032
C	-2.21461	1.544369	0.365958
H	-2.3097	3.67918	0.525139
H	-2.09865	-0.5825	0.557891
C	-2.38277	1.530578	-1.12244
H	-2.01914	-1.78072	2.537995
H	-2.4197	-0.99136	4.070335
H	-0.74894	-1.08035	3.549624
H	-2.31941	4.913613	2.488551
H	-1.01623	4.330317	3.532914
H	-2.68527	4.107522	4.020818
H	-2.34261	0.515127	-1.51658
H	-1.59952	2.113807	-1.61221
H	-3.3405	1.967884	-1.41497
H	0.75192	4.914198	7.698204
H	-0.55129	4.33124	6.653692
H	1.117745	4.108402	6.165774
H	0.45103	-1.7801	7.64735
H	0.851598	-0.99045	6.115159
H	-0.81915	-1.07938	6.63594
H	0.774772	0.514896	11.70241
H	0.031817	2.113618	11.79838
H	1.772782	1.967591	11.60109

Table S32. Geometry optimized coordinates for ***E*-13*** (PBE0-D3/def2-TZVPP).

Atom	x	y	z
P	0.056115	1.767608	5.180212
C	0.310324	1.913283	7.040551
P	-1.94226	1.712671	4.938867
C	-2.00035	1.572405	3.111427
C	1.184264	0.72089	7.435098
C	-0.95321	1.929198	7.882832
C	1.094272	3.210286	7.251157
H	-0.68724	2.020249	8.940903
H	-1.53193	1.012093	7.759003
H	-1.60025	2.769592	7.625091
H	2.00378	3.235895	6.646515
H	1.386712	3.297043	8.30238
H	0.491994	4.084056	6.994539
H	2.099434	0.676227	6.840286
H	0.650518	-0.22295	7.306768
H	1.470661	0.807238	8.488026
C	-2.12853	2.726933	2.328234
C	-2.00865	0.307702	2.50305
C	-2.24764	2.597578	0.949351
C	-2.12175	4.090789	2.94836
C	-2.13093	0.225343	1.12318
C	-1.87463	-0.94601	3.312364
C	-2.24911	1.357872	0.326588
H	-2.34234	3.495086	0.346299
H	-2.13503	-0.755	0.656785
C	-2.39666	1.236503	-1.15904
H	-2.08602	-1.82597	2.70477
H	-2.55704	-0.94646	4.167166
H	-0.86623	-1.04489	3.721054
H	-2.41453	4.850422	2.223498
H	-1.13013	4.345022	3.330696
H	-2.80649	4.14676	3.79972
H	-3.39722	0.885546	-1.42602
H	-1.68291	0.520232	-1.57042
H	-2.23895	2.19554	-1.65296

Table S33. Geometry optimized coordinates for ***E*-14*** (PBE0-D3/def2-TZVPP).

Atom	x	y	z
P	0.326482	1.424161	4.955145
Si	0.421263	1.80837	7.204786

P	-1.62309	1.887707	4.73091
C	-1.88657	1.654455	2.934706
C	2.09422	1.180072	7.770753
C	-0.94303	0.892788	8.102946
C	0.288392	3.651898	7.511244
H	-0.8619	1.053192	9.18145
H	-0.88395	-0.18044	7.912685
H	-1.92508	1.239692	7.776737
H	1.051871	4.199943	6.955656
H	0.413805	3.877531	8.573645
H	-0.68868	4.021768	7.194169
H	2.904819	1.687918	7.243925
H	2.195761	0.108378	7.58767
H	2.226691	1.353238	8.841871
C	-1.86388	2.771081	2.088049
C	-2.20362	0.389215	2.420323
C	-2.14327	2.600704	0.738025
C	-1.52556	4.131702	2.616496
C	-2.47839	0.265148	1.064843
C	-2.24603	-0.82191	3.301963
C	-2.44798	1.355649	0.20474
H	-2.12773	3.469064	0.086754
H	-2.72976	-0.71465	0.67071
C	-2.7184	1.187875	-1.25881
H	-2.678	-1.67222	2.774212
H	-2.83854	-0.64167	4.203636
H	-1.2446	-1.09938	3.638627
H	-1.73379	4.903511	1.875423
H	-0.46918	4.19575	2.889533
H	-2.09641	4.364062	3.520866
H	-3.44553	0.395171	-1.44067
H	-1.8036	0.919763	-1.79518
H	-3.09871	2.109259	-1.70149

Table S34. Geometry optimized coordinates for ***E*-15*** (PBE0-D3/def2-TZVPP).

Atom	x	y	z
P	-0.08995	1.706843	5.165669
O	0.214979	2.004811	6.742521
P	-1.9674	2.385026	4.895422
C	-1.97883	1.915221	3.112961
C	1.485644	1.65463	7.263446
C	-1.61564	2.860542	2.138604
C	-2.45295	0.655349	2.719629
C	-1.72415	2.523112	0.7969
C	-1.11054	4.218809	2.517891

C	-2.54361	0.360693	1.363387
C	-2.85872	-0.37941	3.724102
C	-2.18418	1.277484	0.386886
H	-1.44263	3.258142	0.049277
H	-2.91007	-0.61619	1.064736
C	-2.30401	0.948186	-1.06933
H	-3.38213	-1.20445	3.240636
H	-3.51133	0.042727	4.492769
H	-1.9903	-0.78482	4.248624
H	-1.06362	4.872103	1.646456
H	-0.11227	4.161694	2.958153
H	-1.7511	4.687992	3.269391
H	-2.55297	-0.10237	-1.21969
H	-1.37198	1.155434	-1.59954
H	-3.08482	1.548271	-1.54372
H	1.968511	2.55341	7.649847
H	2.125473	1.206108	6.497214
H	1.349054	0.941163	8.077457

Table S35. Geometry optimized coordinates for ***E*-16*** (PBE0-D3/def2-TZVPP).

Atom	x	y	z
P	0.026534	1.822299	5.154719
N	0.382469	2.095465	6.764087
P	-1.83069	2.588413	4.837005
C	-1.91824	2.01413	3.088116
C	1.729989	1.86212	7.231862
C	-0.50915	2.734233	7.698612
H	-0.4752	3.827149	7.614833
H	-0.23704	2.45505	8.71872
H	-1.53787	2.418519	7.509357
H	2.240555	2.801394	7.473253
H	2.303575	1.351846	6.457744
H	1.721939	1.236139	8.129641
C	-1.50601	2.863973	2.047268
C	-2.48985	0.770598	2.782601
C	-1.66092	2.448808	0.731882
C	-0.89397	4.202578	2.326085
C	-2.62456	0.39464	1.449865
C	-2.95198	-0.16509	3.857583
C	-2.2149	1.215605	0.410348
H	-1.33852	3.110657	-0.06613
H	-3.06575	-0.57035	1.221109
C	-2.37096	0.796623	-1.01948
H	-3.53328	-0.98493	3.434727
H	-3.56554	0.352606	4.599078

H	-2.10571	-0.58958	4.403107
H	-0.79396	4.781343	1.407446
H	0.095889	4.098321	2.776403
H	-1.49477	4.776012	3.036468
H	-2.77222	-0.21413	-1.09503
H	-1.41266	0.820119	-1.54354
H	-3.04709	1.467568	-1.55521

Table S36. Geometry optimized coordinates for ***E*-17*** (PBE0-D3/def2-TZVPP).

Atom	x	y	z
P	-0.14899	1.744513	5.076897
N	0.269205	1.879419	6.951198
P	-2.14647	1.759086	5.005531
C	-2.0556	1.604128	3.181424
C	1.007759	0.639465	7.297694
C	-0.89056	2.038747	7.851992
C	1.171544	3.050436	7.084375
H	-0.53117	2.113104	8.879287
H	-1.54742	1.176843	7.757257
H	-1.4345	2.943507	7.589504
H	2.016214	2.932479	6.407722
H	1.52935	3.109334	8.112904
H	0.623318	3.955556	6.830762
H	1.850779	0.521117	6.618829
H	0.338634	-0.21324	7.202361
H	1.370065	0.714678	8.323685
C	-2.0891	2.767118	2.392187
C	-2.07605	0.327587	2.599538
C	-2.12613	2.622263	1.01576
C	-2.08826	4.131025	3.009211
C	-2.11171	0.240369	1.21505
C	-2.06866	-0.91733	3.431495
C	-2.13218	1.370015	0.406367
H	-2.15922	3.513395	0.398215
H	-2.13411	-0.74116	0.754796
C	-2.17853	1.253557	-1.08299
H	-2.38373	-1.77836	2.844074
H	-2.74307	-0.83475	4.290286
H	-1.07191	-1.12956	3.827558
H	-2.39129	4.886027	2.285492
H	-1.09638	4.402044	3.380751
H	-2.775	4.190238	3.8606
H	-2.12276	0.215176	-1.40594
H	-1.35237	1.800081	-1.54219
H	-3.10366	1.681867	-1.47534

Table S37. Geometry optimized coordinates for ***E*-18*** (PBE0-D3/def2-TZVPP).

Atom	x	y	z
P	0.483799	2.590013	5.061257
P	0.362897	2.040566	7.174805
P	-1.17207	1.461259	4.622837
C	-1.60182	1.526573	2.908398
C	1.706954	2.918914	7.985988
C	0.598274	0.282169	7.472487
C	-1.17444	2.533816	7.967905
H	0.609366	0.084669	8.545814
H	1.542057	-0.03731	7.030825
H	-0.21722	-0.27069	7.006094
H	-1.32293	3.60542	7.836002
H	-1.13465	2.296184	9.032393
H	-2.00317	1.99977	7.503157
H	1.57936	3.992542	7.845508
H	2.65815	2.616922	7.546904
H	1.711499	2.690827	9.052624
C	-0.9544	2.247303	1.8654
C	-2.75311	0.740088	2.602558
C	-1.46192	2.169668	0.584089
C	0.253801	3.085008	2.09719
C	-3.2115	0.705133	1.299654
C	-3.49695	-0.06113	3.632082
C	-2.58705	1.408892	0.274531
H	-0.96527	2.720703	-0.20655
H	-4.08587	0.107587	1.069928
C	-3.10181	1.35632	-1.12317
H	-4.33132	-0.58088	3.164147
H	-3.90114	0.567336	4.427589
H	-2.86022	-0.81197	4.103262
H	0.591927	3.539691	1.167504
H	1.083267	2.49461	2.498796
H	0.054528	3.8934	2.807962
H	-3.97628	0.713912	-1.20922
H	-2.33144	0.984977	-1.80303
H	-3.3733	2.356498	-1.46939

Table S38. Geometry optimized coordinates for H_3PO_4 (PBE0-D3/def2-TZVPP).

Atom	x	y	z
P	-0.08145	-0.03188	-0.05807
O	-0.52214	-0.95434	-1.26126

O	-1.00075	-0.58465	1.110332
O	-0.74148	1.393946	-0.34356
O	1.355506	0.000635	0.163731
H	-1.4631	-0.92855	-1.45296
H	-0.73898	-0.25708	1.974275
H	-0.08801	2.010218	-0.68479

5. References.

1 D. Wang, C. Zhai, Y. Chen, Y. He, X.-d. Chen, S. Wang, L. Zhao, G. Frenking, X. Wang, G. Tan, *Nat. Chem.* **2022**, *15*, 200–205.

2 P. J. Bailey, R. A. Coxall, C. M. Dick, S. Fabre, L. C. Henderson, C. Herber, S. T. Liddle, D. Loroño-González, A. Parkin, S. Parsons, *Chem.–Eur. J.* **2003**, *9*, 4820–4828.

3 Rigaku Oxford Diffraction, *CrysAlis[Pro]* **2020**.

4 O. V. Dolomanov, L. J. Bourhis, R. J. Gildea, J. A. K. Howard, H. Puschmann, *J. Appl. Crystallogr.* **2009**, *42*, 339–341.

5 G. M. Sheldrick, *Acta Crystallogr. Sect. A* **2015**, *71*, 3–8.

6 G. M. Sheldrick, *Acta Crystallogr. Sect. C* **2015**, *71*, 3–8.

7 P. Müller, *Crystallogr. Rev.* **2009**, *15*, 57–83.

8 F. Neese, *Wiley Interdiscip. Rev.-Comput. Mol. Sci* **2012**, *2*, 73–78.

9 M. Yoshifuji, I. Shima, N. Inamoto, K. Hirotsu, T. Higuchi, *J. Am. Chem. Soc.* **1981**, *103*, 4587–4589.

10 A. D. Becke, *Phys. Rev. A* **1988**, *38*, 3098–3100.

11 S. Grimme, J. Antony, S. Ehrlich, H. Krieg, *J. Chem. Phys.* **2010**, *132*.

12 J. P. Perdew, K. Burke, M. Ernzerhof, *Phys. Rev. Lett.* **1996**, *77*, 3865–3868.

13 J. P. Perdew, M. Ernzerhof, K. Burke, *J. Chem. Phys.* **1996**, *105*, 9982–9985.

14 F. Weigend, R. Ahlrichs, *Phys. Chem. Chem. Phys.* **2005**, *7*, 3297–3305.

15 F. Weigend, *Phys. Chem. Chem. Phys.* **2006**, *8*, 1057–1065.

16 F. Neese, F. Wennmohs, A. Hansen, U. Becker, *Chem. Phys.* **2009**, *356*, 98–109.

17 D. A. Pantazis, X.-Y. Chen, C. R. Landis, F. Neese, *J. Chem. Theory Comput.* **2008**, *4*, 908–919.

18 D. A. Pantazis, F. Neese, *J. Chem. Theory Comput.* **2009**, *5*, 2229–2238.

19 D. A. Pantazis, F. Neese, *J. Chem. Theory Comput.* **2011**, *7*, 677–684.

20 D. A. Pantazis, F. Neese, *Theor. Chem. Acc.* **2012**, *131*, 1292.

21 R. F. W. Bader, *Chem. Rev.* **1991**, *91*, 893–928.

22 E. D. Glendening, C. R. Landis, F. Weinhold, *J. Comput. Chem.* **2019**, *40*, 2234–2241.

23 16.2.1 ed., http://www.jmol.org/, **2024**.

24 F. Neese, *J. Comput. Chem.* **2003**, *24*, 1740–1747.

25 E. Caldeweyher, C. Bannwarth, S. Grimme, *J. Chem. Phys.* **2017**, *147*.

26 S. Grimme, C. Bannwarth, S. Dohm, A. Hansen, J. Pisarek, P. Pracht, J. Seibert, F. Neese, *Angew. Chem., Int. Ed.* **2017**, *56*, 14763–14769.

27 G. L. Stoychev, A. A. Auer, R. Izsák, F. Neese, *J. Chem. Theory Comput.* **2018**, *14*, 619–637.

28 E. Caldeweyher, S. Ehlert, A. Hansen, H. Neugebauer, S. Spicher, C. Bannwarth, S. Grimme, *J. Chem. Phys.* **2019**, *150*.

29 E. Caldeweyher, J.-M. Mewes, S. Ehlert, S. Grimme, *Phys. Chem. Chem. Phys.* **2020**, *22*, 8499–8512.

30 B. Helmich-Paris, B. de Souza, F. Neese, R. Izsák, *J. Chem. Phys.* **2021**, *155*.

31 F. Neese, *J. Comput. Chem.* **2022**, *44*, 381–396.

32 L. Wittmann, I. Gordiy, M. Friede, B. Helmich-Paris, S. Grimme, A. Hansen, M. Bursch, *Phys. Chem. Chem. Phys.* **2024**, *26*, 21379–21394.

33 J. A. Smith, K. D. Moeller, *Org. Lett.* **2013**, *15*, 5818–5821.

34 T. Matsuo, K. Suzuki, T. Fukawa, B. Li, M. Ito, Y. Shoji, T. Otani, L. Li, M. Kobayashi, M. Hachiya, Y. Tahara, D. Hashizume, T. Fukunaga, A. Fukazawa, Y. Li, H. Tsuji, K. Tamao, *Bull. Chem. Soc. Jpn.* **2011**, *84*, 1178–1191.

35 K. M. Carsch, I. M. DiMucci, D. A. Iovan, A. Li, S.-L. Zheng, C. J. Titus, S. J. Lee, K. D. Irwin, D. Nordlund, K. M. Lancaster, T. A. Betley, *Science* **2019**, *365*, 1138–1143.

36 L. J. Irwin, J. H. Reibenspies, S. A. Miller, *J. Am. Chem. Soc.* **2004**, *126*, 16716–16717.

37 D. Perales, R. Bhowmick, M. Zeller, P. Miro, B. Vlaisavljevich, S. C. Bart, *Chem. Commun.* **2022**, *58*, 9630–9633.

38 C. Hu, N. H. Rees, M. Pink, J. M. Goicoechea, *Nat. Chem.* **2024**, *16*, 1855–1860.

39 M. Wu, H. Li, W. Chen, D. Wang, Y. He, L. Xu, S. Ye, G. Tan, *Chem* **2023**, *9*, 2573–2584.

40 J. Bresien, C. Hering, A. Schulz, A. Villinger, *Chem.–Eur. J.* **2014**, *20*, 12607–12615.

41 M. Yoshifuji, K. Shibayama, N. Inamoto, T. Matsushita, K. Nishimoto, *J. Am. Chem. Soc.* **1983**, *105*, 2495–2497.

42 F. P. Gabbaï, P. J. Chirik, D. E. Fogg, K. Meyer, D. J. Mindiola, L. L. Schafer, S.-L. You, *Organometallics* **2016**, *35*, 3255–3256.

43 C. Brunet, R. Antoine, M. Broyer, P. Dugourd, A. Kulesza, J. Petersen, M. I. S. Röhr, R. Mitrić, V. Bonačić-Koutecký, R. A. J. O'Hair, *J. Phys. Chem. A.* **2011**, *115*, 9120–9127.
